# Cobaltoceniumselenolate Gold(I) Complexes: Synthesis, Spectroscopic, Structural and Anticancer Properties

**DOI:** 10.1002/ejic.202100379

**Published:** 2021-06-15

**Authors:** Daniel Menia, Holger Kopacka, Klaus Wurst, Thomas Müller, Petra Lippmann, Ingo Ott, Benno Bildstein

**Affiliations:** ^1^ Institute of General, Inorganic and Theoretical Chemistry University of Innsbruck Center for Chemistry and Biomedicine Innrain 80–82 6020 Innsbruck Austria; ^2^ Institute of Organic Chemistry University of Innsbruck Center for Chemistry and Biomedicine Innrain 80–82 6020 Innsbruck Austria; ^3^ Institute of Medicinal and Pharmaceutical Chemistry Technische Universität Braunschweig Beethovenstr. 55 38106 Braunschweig Germany

**Keywords:** Cobalt, Selenium, Gold, Sandwich complexes, Cytotoxicity

## Abstract

Cobaltoceniumselenolate is an unusual, highly air‐sensitive, mesoionic compound containing a very soft anionic selenium donor atom. Here we explore its coordination chemistry with Au(I) metal centers and show that its hetero‐ and homoleptic gold complexes are highly colored, air‐stable compounds, which were characterized by ^1^H/^13^C/^31^P/^77^Se NMR, IR, UV‐Vis, HR‐MS and single crystal XRD. Cytotoxicity of these polar, water‐soluble complexes was studied against various standard cancer cell lines (A549MDA‐MB‐231, HT‐29) revealing good anticancer activity of all three complexes.

## Graphical abstract

Air‐stable homo‐ and heteroleptic gold(I) complexes containing the unusual zwitterionic cobaltoceniumselenolate ligand were synthesized and fully characterized. First preliminary biological test against three standard human cancer cell lines showed good cytotoxicity properties, indicating that cobaltocenium selenolate is a promising ligand for future developments of redox‐active organometallic metallo‐drugs for anticancer therapy.

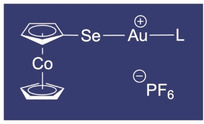

The rich coordination chemistry of organic selenium ligands with soft d^10^ coinage metal centers [Cu(I), Ag(I), Au(I)] is largely dominated by air‐sensitive anionic selenolates (R−Se^−^, R=alkyl or aryl)^[1]^ and air‐stable neutral cyclic selenoureas.^[2]^ The latter compounds are in great structural variety easily available by simple synthesis from their corresponding N‐heterocyclic carbenes (NHCs) or NHC‐precursors^[3]^ via direct selenation or one‐pot deprotonation‐selenation, respectively.[^2^, ^3^] The major interest in cyclic selenoureas is currently based on their widespread use as a convenient ^77^Se NMR probe to quantify the π‐acidity of their parent NHCs.^[4]^

Recently we reported on cobaltocenylidene (CcC), a mesoionic, very electron‐rich metalloceno carbene, stabilized in a gold(III) complex, and on its cobaltoceniumselenolate derivative CcSe (**1**) (Scheme 1),^[5]^ that was prepared to evaluate the σ‐donor character and π‐backbonding ability of this unusual redox‐responsive organometallic carbene by its ^77^Se NMR properties. Cobaltoceniumselenolate (**1**) is an extremely air‐sensitive, dark purple compound with a zwitterionic structure composed of an undistorted cationic cobaltocenium moiety with an anionic selenido substituent, as shown by single crystal structure analysis (Scheme 1).^[5]^ Compared to the air‐stable selenium derivatives of standard NHCs, cyclic selenoureas,^[2]^ which feature a selenium‐carbon double bond, **1** is electronically clearly distinctly different and represents the unusual case of a neutral selenolate ligand. In addition, contrarious steric properties are evident for **1** (axial shielding by the cobaltocenium moiety) and cyclic selenoureas (peripheral shielding by the wingtip substituents). Hence we became interested to investigate Au(I) complexes of **1** in comparison to their cyclic selenourea complexes and for potential applications as new metallodrugs, inspired by current studies in anticancer research on redox‐active metal complexes,^[6]^ gold anticancer metallodrugs,^[7]^ and organoselenium anticancer agents.^[8]^

**Scheme 1:**
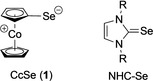
Pertinent Lewis valence structures of cobaltoceniumselenolate (**1**, left) and standard NHC selenium derivatives, cyclic selenoureas (NHC−Se, right).

**Synthesis**: Cobaltoceniumselenolate (**1**) is available from iodocobaltocenium hexafluoridophosphate^[9]^ by a nucleophilic aromatic substitution with sodium selenide under strictly inert conditions as recently published.^[5]^ In‐situ synthesis of **1** followed by oxidation on air led to its dicationic diselenide bis(hexafluoridophosphate) **2 a** [CcSeSeCc](PF_6_)_2_ in a satisfying yield of 75 % (Scheme 2). Because it proved quite difficult to obtain suitable single crystals for **2 a**, we synthesized also its tetraphenylborate analog **2 b** [CcSeSeCc][B(C_6_H_5_)_4_]_2_ in a similar manner using sodium tetraphenylborate in the work‐up procedure (see Supporting Information). As desired, good‐quality single crystals of **2 b** could be obtained for the XRD analysis discussed below. Reaction of the cobaltoceniumselenolate (**1**) with (triphenylphosphine)gold chloride afforded either hetero or homoleptic complexes, depending on stoichiometry (Scheme 2). In a 1 : 1 CcSe:Au ratio, heteroleptic [(CcSe)(PPh_3_)Au]PF_6_ (**3**) was obtained (61 % yield), whereas a 2 : 1 CcSe:Au ratio afforded homoleptic [(CcSe)_2_Au]PF_6_ (**4**) in 56 % yield.

**Scheme 2:**
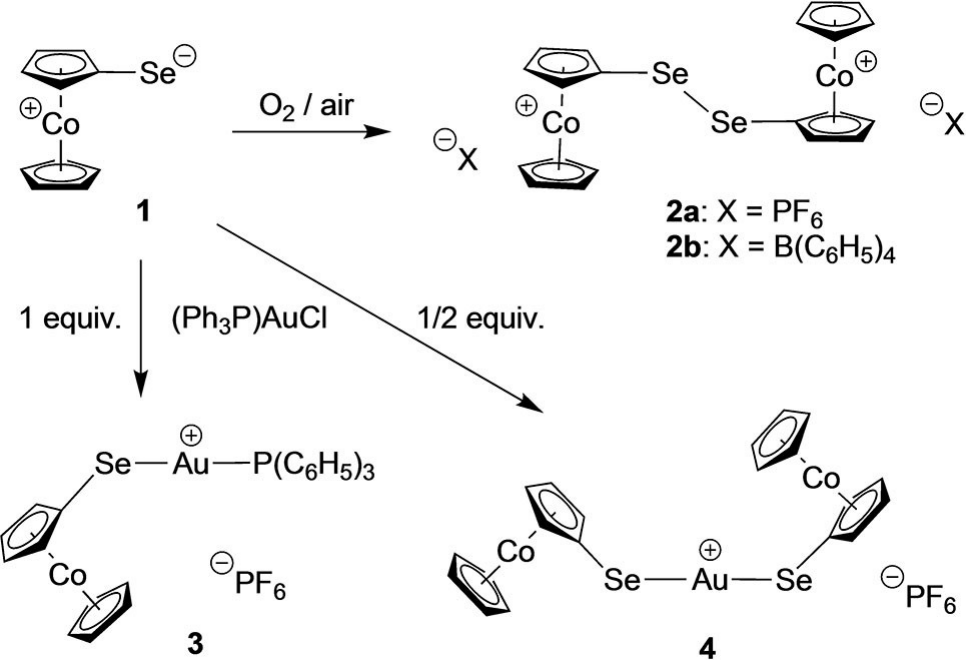
Synthesis of compounds **2 a**, **2 b**, **3** and **4**.

**Physical, spectroscopic and structural properties** (for details and spectra see Supporting Information): Complexes **2 ab**, **3** and **4** are air‐stable salts with melting points from 135–162 °C, soluble in polar solvents like acetonitrile, dimethylformamide, dimethylsulfoxide, acetone, nitromethane, methanol and to a lesser degree in dichloromethane and water. Whereas dicationic diselenides **2 ab** are yellow compounds, monocationic gold(I) selenolate complexes **3** and **4** are highly colored dark‐red materials, due to their strong selenium‐gold charge‐transfer absorptions (Figure 1). ^1^H NMR spectra of **2 ab**, **3** and **4** showed the typical pattern of monosubstituted metallocenes [s(5H), Cp and 2×pseudo‐t(2H), substituted Cp] in the usual spectral region of cobaltocenium salts (5.3–6.0 ppm), in addition to phenyl‐hydrogen signals in the aromatic region for tetraphenylborate salt **2 a** and triphenylphosphine complex **3**. ^13^C NMR spectra of **2 a**, **3** and **4** displayed their cobaltocenium signals at 85–89 ppm (Cp and C−H carbons of substituted Cp) and those of the substituted carbon resonances at 96.2 (**2 a**), 104 (**3**) and 88.6 (**4**), indicative of the difference in their structure [diselenide **2 a** versus heteroleptic **3** and homoleptic **4** CcSe Au(I) complexes). For **3** the ^31^P NMR signals were observed at 39.2 ppm [PPh_3_ coordinated to Au(I)] and −143.3 ppm [PF_6_^−^, septet, ^1^J(^19^F−^31^P)=706 Hz]. ^77^Se NMR chemical shifts of **2 a** and **3** were detected at 429 and 596 ppm versus dimethylselenide as reference. Unfortunately, no signal could be observed for complex **4**, even on very long data acquisition periods, probably due to poor relaxation properties. In comparison to the ^77^Se signal of the free CcSe ligand [δ(^77^Se)=258 ppm],^[5]^ the Au(I)‐coordinated CcSe ligand in complex **3** [δ(^77^Se)=596 ppm] is highly deshielded by 338 ppm. IR spectra of **2 a**, **3** and **4** are rather simple with the most prominent signals at approximately 810 and 550 cm^−1^ arising from the strong ν_P‐F_ absorptions of the hexafluoridophosphate counterions. The identity of compounds **2 a**, **3** and **4** is further corroborated by their high‐resolution mass spectra with excellent agreement of experimental with calculated values.

**Figure 1:**
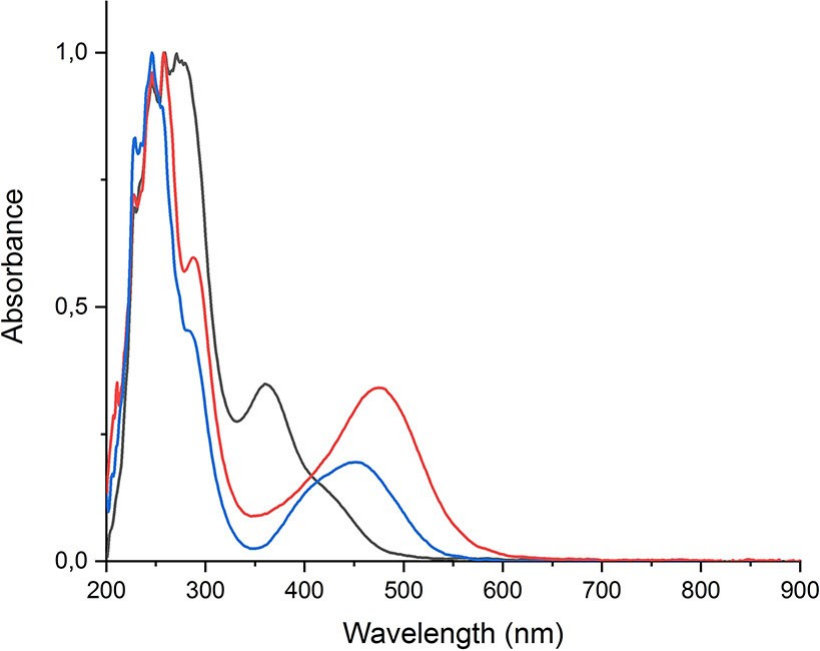
Overlay of UV‐vis spectra of **2 a** (black line), **3** (blue line) and **4** (red line).

Single crystal structure analyses are available for **2 b**, **3** and **4** (Figure 2 and Supporting Information) with good R_1_ values of 4.49, 4.61 and 4.86 %, respectively. The cobaltocenium substituents of all three compounds are undistorted and bond distances at the selenium atoms (**2 b**: Se−Se=2.310 Å, **3**: Se−Au=2.425 Å, **4**: Se−Au=2.398 Å) are comparable to those of non‐cobaltocenium dicationic diselenides^[10]^ or NHC−Se‐Au(I) complexes.^[2d]^ Bond angles at the tetrahedral selenium atoms of **2 b**, **3** and **4** (99–105°) are slightly compressed in comparison to the standard value of 109.5°, as predicted by VSEPR theory by greater repulsion between electron lone pairs than between bonding pairs. The coordination geometry at the gold(I) centers of complexes **3** and **4** is more or less linear (**3**: 170.3°, **4**: 177.8°), as anticipated and similar as in NHC−Se‐Au(I) complexes.^[2d]^ One might expect either aurophilic^[11]^ Au^…^Au or chalcogenophilic^[12]^ Se^…^Se intermolecular bonding for these di/mono‐cationic Se/Au species **2 b**, **3** and **4**, however, no such intermolecular secondary bonds were observed (compare Supporting Information), most likely due to the position of the tetraphenylborate or hexafluoridophosphate counterions in the crystal lattice. In comparison, NHC−Se gold(I) complexes containing bulky wingtip substituents display dimeric solid state structures with weak Se^…^Se interactions.^[10a]^

**Figure 2:**
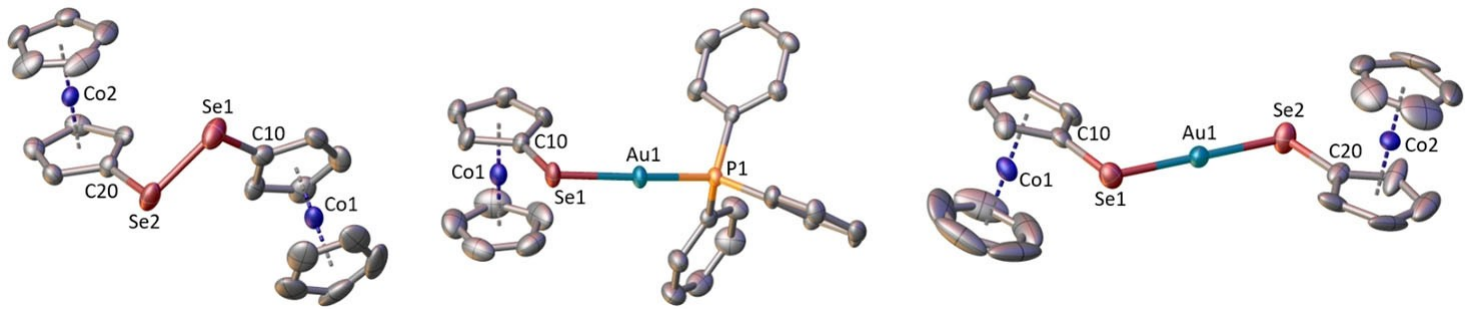
Molecular structures of **2 b** (left), **3** (middle) and **4** (right). Counteranions tetraphenylborate (for **2 b**) or hexafluoridophosphate (for **3** and **4**) omitted for clarity. Selected distances (Å) and angles (°) for **2 b**: Se(1)−Se(2)=2.3104(14), Se(1)−C(10)=1.903(7), Se(2)−C(20)=1.902(7); C(10)−Se(1)−Se(2)=101.0(2), C(20)−Se(2)−Se(1)=100.4(2). Selected distances (Å) and angles (°) for **3**: Au(1)−Se(1)=2.4249(6), Au(1)‐P(1)=2.2673(13), Se(1)−C(10)=1.891(5); C(10)−Se(1)−Au(1)=99.01(16), P(1)−Au(1)−Se(1)=170.37(4). Selected distances (Å) and angles (°) for **4**: Au(1)−Se(1)=2.3979(7), Au(1)−Se(2)=2.3912(8), Se(1)−C(10)=1.872(6), Se(2)−C(20)=1.870(6); Se(1)−Au(1)‐Se(2)=177.81(2), C(10)−Se(1)−Au(1)=102.85(18), C(20)−Se(2)−Au(1)=105.3(2).

**Cytotoxicity studies**: Three cancer cell lines (A549 lung carcinoma, HT‐29 colon adenocarcocinoma, and MDA‐MB‐231 breast carcinoma), which represent very relevant human cancers were chosen to study to cytotoxic effects of the complexes **2 a**, **3** and **4** (Table 1). Complexes **3** and **4** were more active than **2 a** in all three cell lines. This cleary confirms that the cobaltoceniumselenolate partial structure causes a good to moderate cytotoxic effect, which can be significantly increased by introduction of a gold(I) center. The most sensitive cell line in the studied panel was MDA‐MB‐231. The assay with this cell line requires a 96 h incubation period instead of 72 h as for HT‐29 and A549. The enhanced activity against MDA‐MB‐231 might therefore indicate that the biological activity of the complexes generally increases over time.

**Table 1:** Cytotoxicity activity against 3 cancer cell lines expressed as IC_50_ values. Values were obtained in three independent experiments and are presented as mean values±standard deviation.

	**A549**	**HT‐29**	**MDA‐MB‐231**
**2 a**	17.4±0.1 μM	51.6±2.2 μM	11.5±1.6 μM
**3**	8.4±0.9 μM	5.0±0.2 μM	3.6±0.3 μM
**4**	12.3±1.5 μM	4.9±0.3 μM	3.5±0.4 μM

**Summary**: Starting from the zwitterionic cobaltoceniumselenolate ligand, CcSe, its diselenide oxidation product [CcSeSeCc]^2+^ and monocationic hetero/homoleptic [(CcSe)(PPh_3_)Au]PF_6_/[(CcSe)_2_Au]PF_6_ gold(I) complexes were obtained. All three compounds are air‐stable materials that were fully characterized by spectroscopic techniques (multinuclear NMR, IR, HR‐MS, UV‐Vis, XRD). Cytotoxic effects were observed with all three complexes against three selected cancer cell lines. Introduction of the gold(I) center significantly increased the cytotoxic activity of the cobaltoceniumselenolates.

Supporting Information (see footnote on the first page of this article): Experimental details, spectra, X‐ray and crystallographic refinement details.

Deposition Numbers 2081773 (for **2 b**), 2081774 (for **3**) and 2081775 (for **4**) contain the supplementary crystallographic data for this paper. These data are provided free of charge by the joint Cambridge Crystallographic Data Centre and Fachinformationszentrum Karlsruhe Access Structures service www.ccdc.cam.ac.uk/structures.

## Conflict of interest

The authors declare no conflict of interest.

## Supporting information

As a service to our authors and readers, this journal provides supporting information supplied by the authors. Such materials are peer reviewed and may be re‐organized for online delivery, but are not copy‐edited or typeset. Technical support issues arising from supporting information (other than missing files) should be addressed to the authors.

Supplementary
