# Supplementary material for: Cobaltoceniumselenolate Gold(I) Complexes: Synthesis, Spectroscopic, Structural and Anticancer Properties
Source: Eur J Inorg Chem. 2021 Jun 15;2021(27):2784–6. doi: 10.1002/ejic.202100379 (PMC8362036; doi:10.1002/ejic.202100379)
Supplement: Supplementary file 1 — Supplementary [file EJIC-2021-2784-s001.pdf]

European
Journal of
Inorganic Chemistry

Supporting Information

Cobaltoceniumselenolate Gold(I) Complexes: Synthesis, Spectroscopic, Structural and Anticancer Properties

Daniel Menia, Holger Kopacka, Klaus Wurst, Thomas Müller, Petra Lippmann, Ingo Ott,* and Benno Bildstein*

Table of Contents

1 Dicobaltoceniumdiselenide Bis(hexafluoridophosphate) (**2a**) 2

1.1 Analytical Data 2

1.2 Spectra 3

2 Dicobaltoceniumdiselenide Bis(tetraphenylborate) (**2b**) 6

2.1 Analytical Data 6

2.2 Spectra 7

2.3 Crystallographic Data 8

3 (Cobaltoceniumselenolate)(triphenylphosphane)gold(I) Hexafluoridophosphate (**3**) 22

3.1 Analytical Data 22

3.2 Spectra 23

3.3 Crystallographic Data 27

4 Di(cobaltoceniumselenolate)gold(I) Hexafluoridophosphate (**4**) 37

4.1 Analytical Data 37

4.2 Spectra 38

4.3 Crystallographic Data 41

5 Cell culture and Cytotoxicity 54

6 References 55

7 List of Figures 55

8 List of Tables 55

1 Dicobaltoceniumdiselenide Bis(hexafluoridophosphate) (**2a**)

In a 100 ml Schlenk flask, 40 mg sodium selenide (2.4 eq., 0.320 mmol) and 122 mg iodocobaltocenium hexafluoridophosphate[1] (2.0 eq., 0.267 mmol) were dissolved in 20 ml of dry THF. This mixture was stirred at room temperature for 24 hours under an argon atmosphere. Then the mixture was charged with 40 µl of an aqueous hexafluoridophosphoric acid solution (60% w/w, 2.0 eq., 0.267 mmol) and the yellow product starts to precipitate. In order to complete the precipitation and to oxidize unreacted starting materials, the reaction was stirred under ambient conditions for 24 hours. Then 5 ml of toluene was added and THF was removed on a rotary evaporator. The dark brown precipitant was filtered off using a pleated filter and washed with diethyl ether. The product was dissolved in 50 ml warm acetonitrile (50°C) and 50 ml of toluene was added. Using a rotary evaporator, the acetonitrile and half of the toluene was removed, and the brownish/yellow product precipitated. The mixture was cooled to -20°C and cold filtered using a porcelain filter crucible and washed with diethyl ether to obtain the pure brownish/yellow product in 75% yield (0.099 mmol, 82 mg).

1.1 Analytical Data

^{1}H NMR (300 MHz, CD_3CN): δ 6.06 (pseudo-t,^3J = 2.0 Hz, 4H, Cp-Se), 5.73 (pseudo-t,^3J = 2.0 Hz, 4H, Cp-Se), 5.69 (s, 10H, Cp), 2.16 (H_2O), 1.94 (CD_3CN) ppm.

^{13}C NMR (75 MHz, CD_3OD): δ 96.22 (Cp-Se ipso), 88.41 (Cp-Se), 87.90 (Cp), 86.77 (Cp-Se), 49.14 (CD_3OD) ppm.

^{77}Se NMR (57 MHz, CD_3OD): δ -871.77 (vs. H_2SeO_3), δ 429.38 (vs. MeSeMe) ppm.

MS (ESI+): *m/z* calc. 267.9196 (M^{2+}), found 267.9189 (M^{2+}).

UV-Vis (CH_3CN): λ_{max} 259 nm (ε 20590 L mol^{-1} cm^{-1}), 360 nm (ε 7175 L mol^{-1} cm^{-1}).

IR (ATR): 3117 w, 2948 w / 2917 w / 2850 w ($\nu_{C–H}$), 1726 w, 1411 w, 1387 w ($\nu_{C–C}$), 1265 w, 1112 w / 1073 w (δ_{C-C}), 1030 w (δ_{C-H}), 865 w (CH_{oop}), 808 s ($\nu_{P–F}$), 554 s ($\nu_{P–F}$), 506 w, 461 s ($\nu_{Co–C}$) cm^{-1}.

Melting point: 150.3°C (dec.)

1.2 Spectra

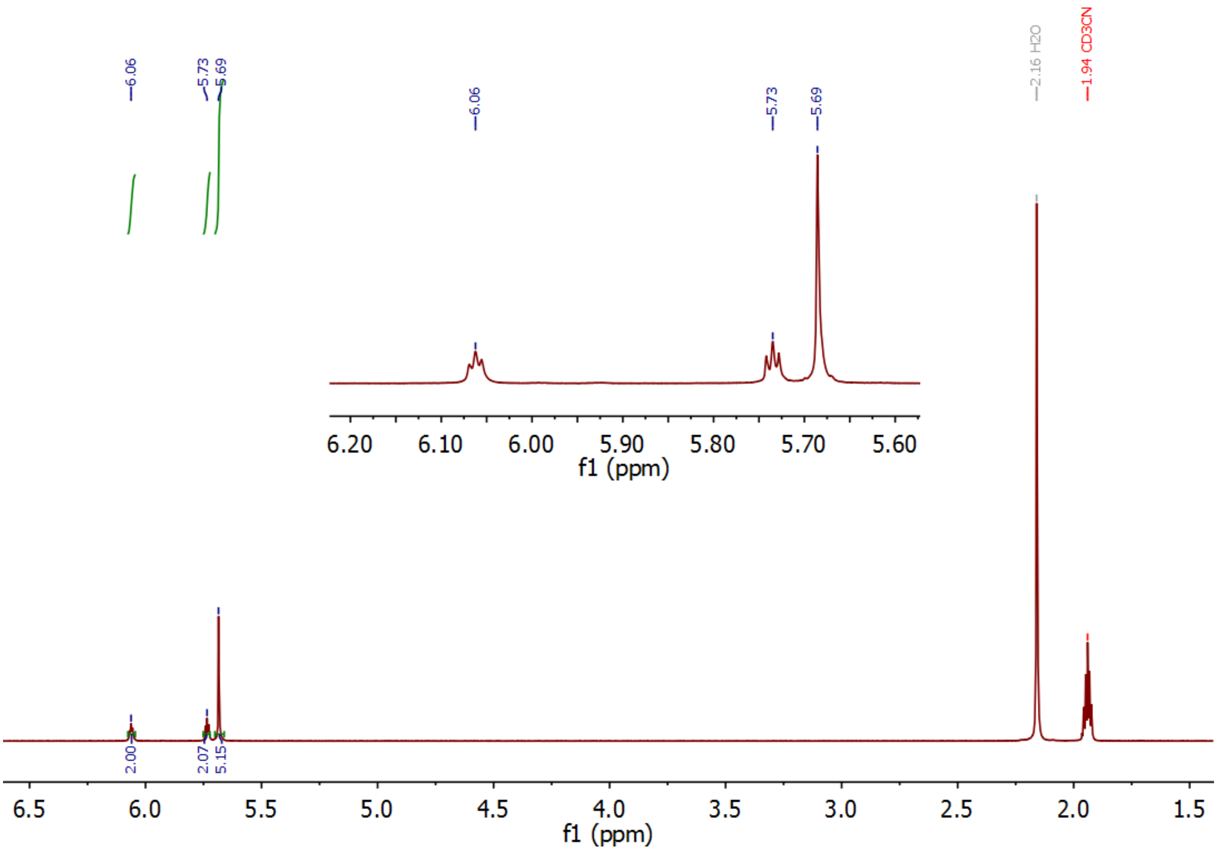

*Figure S1: ^{1}H NMR (300 MHz, CD_3CN) **2a**.*

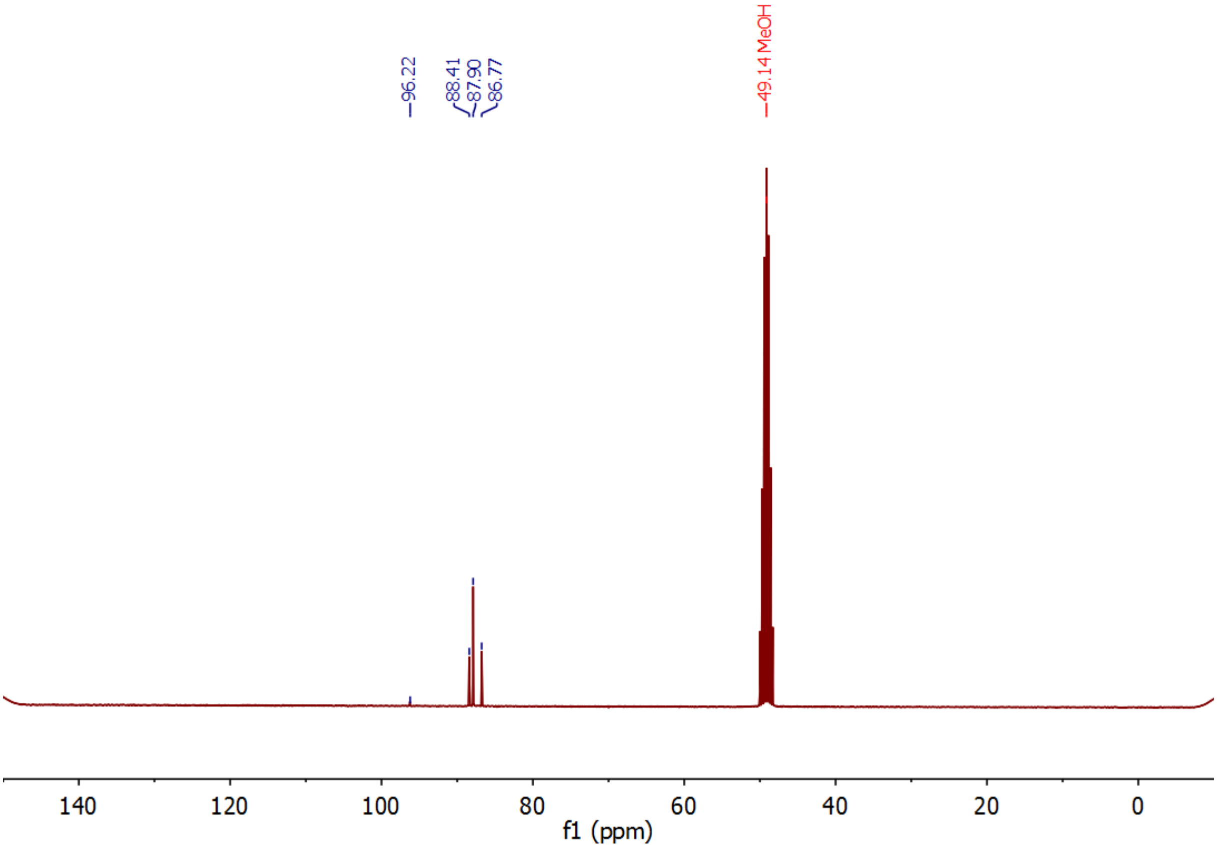

*Figure S2: ^{13}C NMR (75 MHz, CD_3OD) **2a**.*

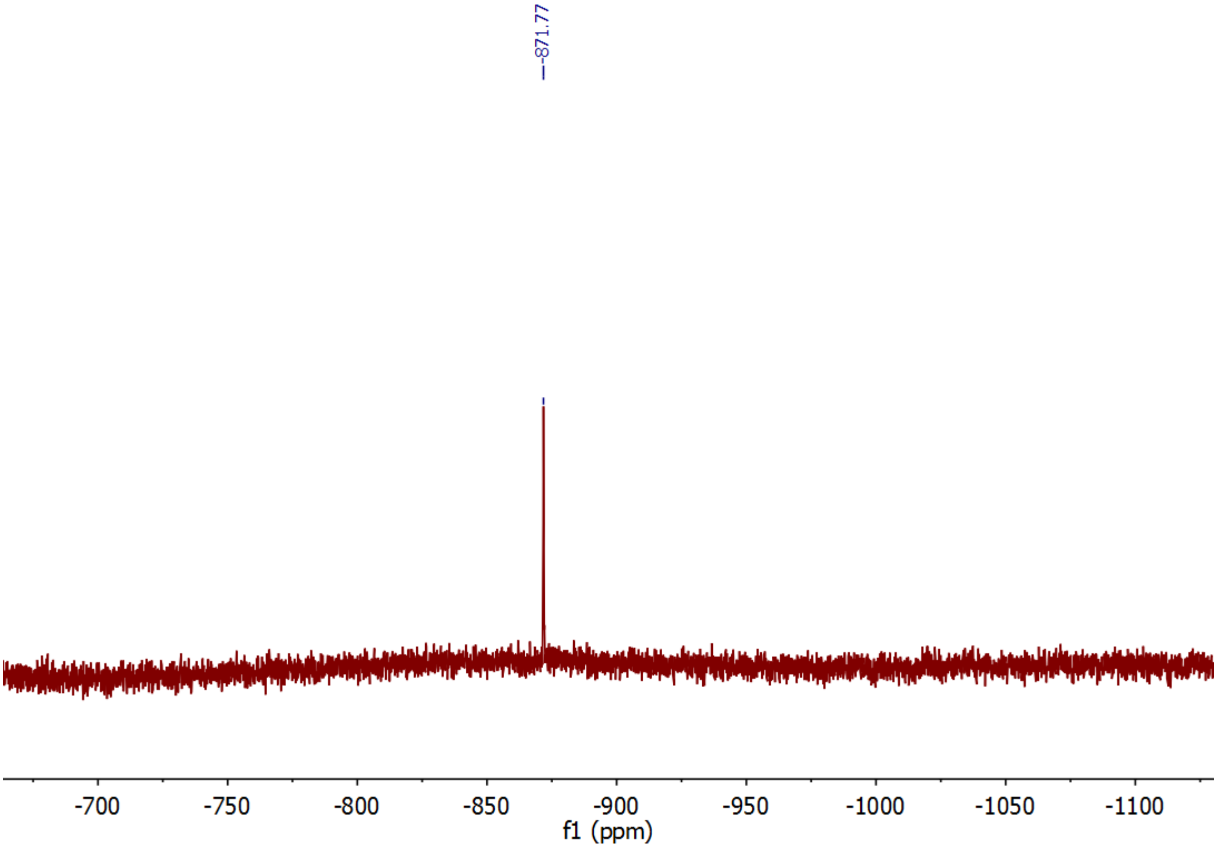

Figure S3: ^{77}*Se NMR (57 MHz,* CD_3OD*)* ***2a****.*

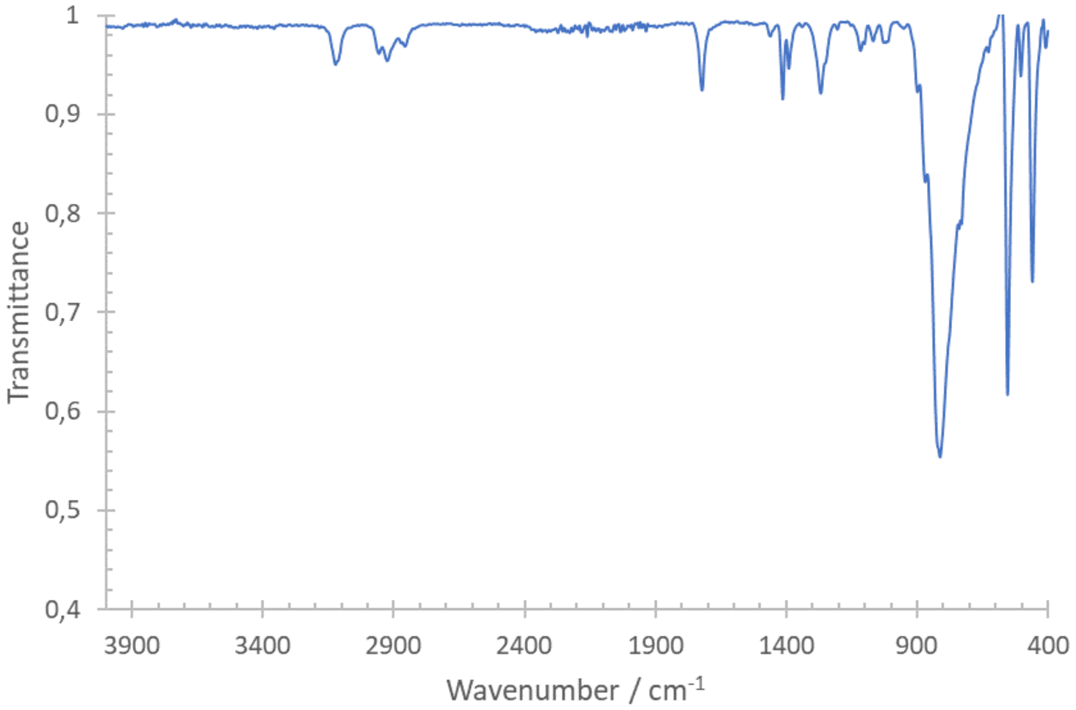

Figure S4: IR (ATR) ***2a****.*

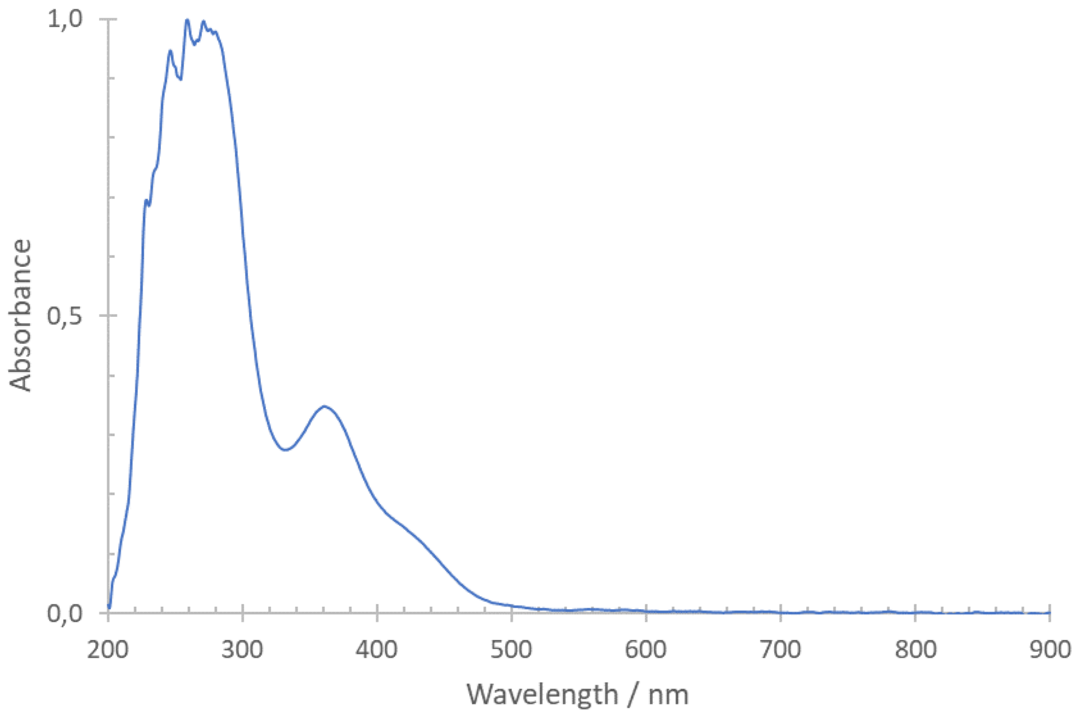

Figure S5: UV-Vis (CH_3CN) ***2a****.*

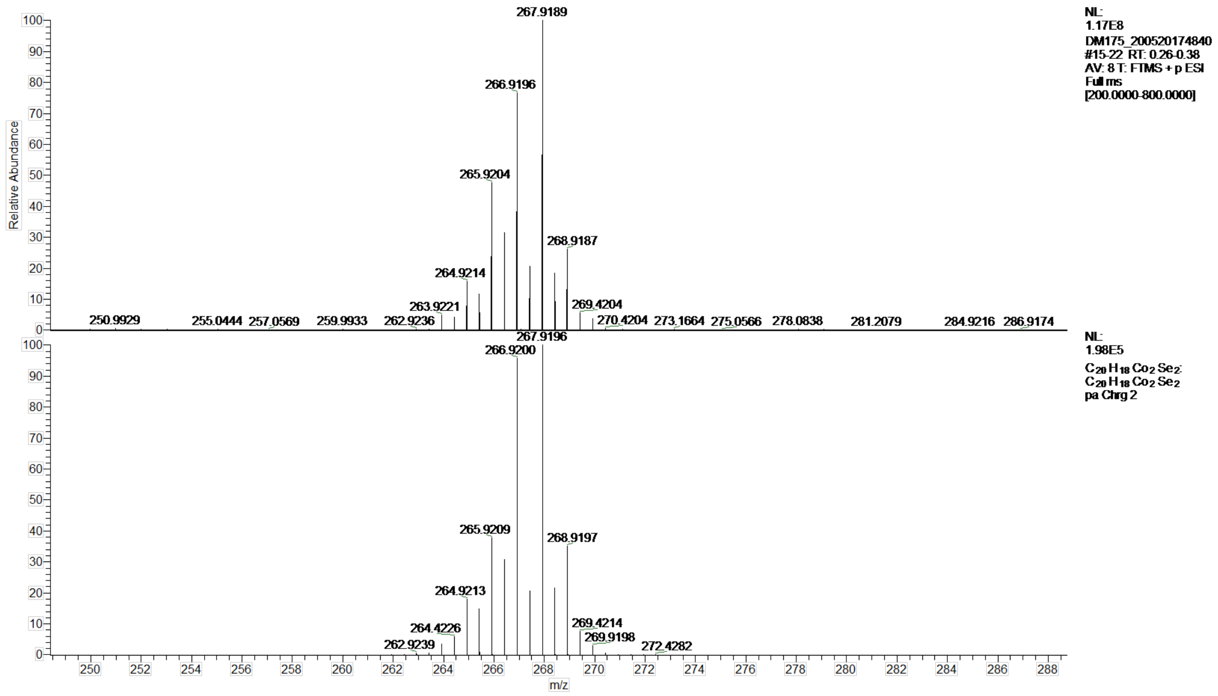

Figure S6: MS (ESI+) ***2a****.*

2 Dicobaltoceniumdiselenide Bis(tetraphenylborate) (**2b**)

In a 100 ml Schlenk flask, 14 mg sodiumselenide (2.4 eq., 0.112 mmol) and 42.9 mg iodocobaltocenium hexafluoridophosphate[1] (2.0 eq., 0.093 mmol) was dissolved in 10 ml dry acetonitrile. This mixture was stirred at room temperature for 24 hours under an argon atmosphere. Then the mixture was charged with 20 µl of concentrated hydrochloric acid (5.2 eq., 0.240 mmol). 1 ml of methanol was added, and the reaction was stirred under ambient conditions for 24 hours in order to fully oxidize the intermediate and unreacted starting materials. The dark yellow precipitant was filtered off, washed with diethyl ether and the yellow intermediate was dissolved with methanol. The solvent was removed on a rotary evaporator and the residue taken up in 10 ml acetone. To this yellow solution 63.6 mg sodium tetraphenylborate (4.0 eq., 0.186 mmol) was added and the mixture was stirred for 24 hours under ambient conditions. The precipitated bright yellow product was filtered off, washed with acetone and dissolved with acetonitrile. The solvent was removed on a rotary evaporator to obtain the product in 27% yield (0.013 mmol, 14.9 mg). Single crystals were obtained by diffusion−crystallization from acetonitrile/diethyl ether at 4 °C.

2.1 Analytical Data

^{1}H NMR (300 MHz, CD_3CN): δ 7.27 (m, 16H, BPh_4), 6.99 (m, 16H, BPh_4), 6.84 (m, 8H, BPh_4), 5.86 (pseudo-t,^3J = 2.0 Hz, 4H, Cp-Se), 5.70 (pseudo-t,^3J = 2.0 Hz, 4H, Cp-Se), 5.61 (s, 10H, CcSe), 3.43 (Et_2O), 2.14 (H_2O), 1.12 (Et_2O), 1.94 (CD_3CN) ppm.

2.2 Spectra

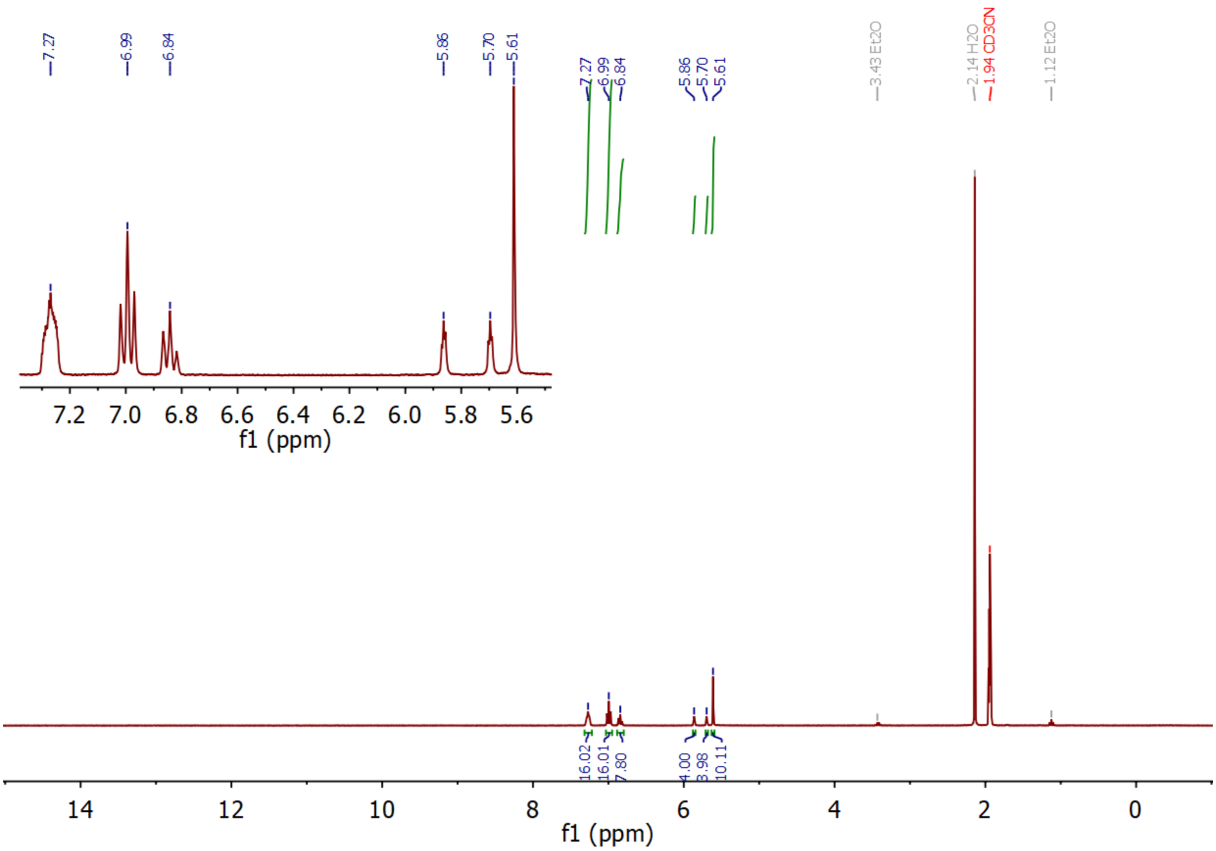

*Figure S7: ^{1}H NMR (300 MHz, CD_3CN) **2b**.*

2.3 Crystallographic Data

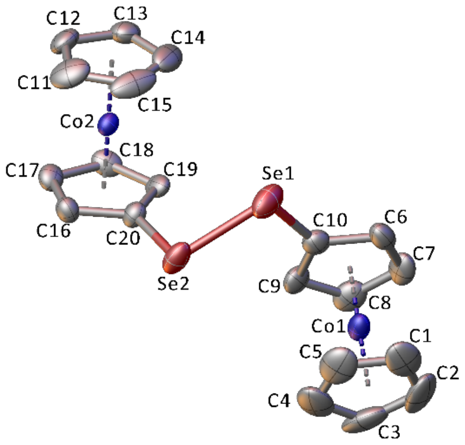

Counterions tetraphenylborate omitted for clarity.

Table S1. Crystal data and structure refinement for **2b**.		
Empirical formula	C_{68} H_{58} B_2 Co_2 Se_2	
Formula weight	1172.54	
Temperature	173(2) K	
Wavelength	0.71073 Å	
Crystal system	Monoclinic	
Space group	Pn (no. 7)	
Unit cell dimensions	a = 12.0221(12) Å	α = 90°
	b = 16.7575(16) Å	β = 91.716(3)°
	c = 13.2119(12) Å	γ = 90°
Volume	2660.5(4) $Å^3$	
Z	2	
Density (calculated)	1.464 Mg/m^3	
Absorption coefficient	2.035 mm^{-1}	
F(000)	1196	
Crystal size	0.180 x 0.140 x 0.030 mm^3	
Theta range for data collection	2.257 to 25.000°.	
Index ranges	-14 ≤ h ≤ 14, -19 ≤ k ≤ 19, -15 ≤ l ≤ 15	
Reflections collected	42616	
Independent reflections	9001 [R(int) = 0.0296]	
Completeness to theta = 25.000°	99.9 %	
Absorption correction	Semi-empirical from equivalents	
Max. and min. transmission	0.813 and 0.710	
Refinement method	Full-matrix least-squares on F^2	
Data / restraints / parameters	9001 / 2 / 668	
Goodness-of-fit on F^2	1.031	
Final R indices [I>2sigma(I)]	R1 = 0.0411, wR2 = 0.1046	
R indices (all data)	R1 = 0.0449, wR2 = 0.1073	
Absolute structure parameter	0.131(13)	
Extinction coefficient	n/a	
Largest diff. peak and hole	2.228 and -0.891 e.$Å^{-3}$	

Table S2. Atomic coordinates (x 10^4) and equivalent isotropic displacement parameters (Å^2 x 10^3) for **2b**. U(eq) is defined as one third of the trace of the orthogonalized U^{ij} tensor.

	x	y	z	U(eq)
Se(1)	7597(1)	7930(1)	4301(1)	58(1)
Se(2)	9411(1)	8199(1)	4819(1)	47(1)
Co(1)	8309(1)	6018(1)	3299(1)	33(1)
Co(2)	8306(1)	8872(1)	7128(1)	25(1)
B(1)	3499(5)	4972(4)	3566(5)	22(1)
B(2)	3203(6)	9950(4)	6246(5)	22(1)
C(1)	8268(9)	6194(8)	1791(6)	63(3)
C(2)	8539(11)	5386(8)	2036(8)	82(4)
C(3)	9525(10)	5390(6)	2674(9)	75(3)
C(4)	9845(9)	6205(8)	2788(9)	73(3)
C(5)	9090(9)	6677(7)	2263(7)	65(3)
C(6)	6775(6)	6320(6)	3773(6)	44(2)
C(7)	6995(7)	5515(5)	3995(6)	47(2)
C(8)	7957(8)	5471(5)	4630(6)	49(2)
C(9)	8340(7)	6254(5)	4827(5)	39(2)
C(10)	7604(6)	6794(4)	4301(6)	38(2)
C(11)	8046(8)	10056(5)	7016(7)	55(2)
C(12)	7644(7)	9782(4)	7936(6)	42(2)
C(13)	6833(6)	9194(5)	7709(6)	41(2)
C(14)	6727(7)	9123(6)	6657(7)	55(2)
C(15)	7478(9)	9653(6)	6223(6)	62(3)
C(16)	9959(6)	8673(4)	6908(5)	33(2)
C(17)	9685(6)	8493(4)	7915(6)	34(2)
C(18)	8848(6)	7883(4)	7884(6)	36(2)
C(19)	8600(6)	7692(4)	6862(6)	32(2)
C(20)	9273(6)	8179(4)	6249(5)	30(2)
C(21)	4566(5)	4410(4)	3988(5)	22(1)
C(22)	5076(5)	4488(4)	4951(5)	27(1)
C(23)	5997(6)	4028(4)	5262(5)	31(2)
C(24)	6456(6)	3492(4)	4593(5)	31(2)
C(25)	5958(6)	3388(4)	3657(5)	30(1)
C(26)	5021(6)	3832(4)	3370(5)	28(1)
C(27)	3877(5)	5548(3)	2635(5)	21(1)
C(28)	4758(6)	5397(4)	2008(5)	32(2)
C(29)	5018(6)	5870(4)	1185(5)	34(2)
C(30)	4382(6)	6544(4)	973(5)	32(2)
C(31)	3527(6)	6735(4)	1595(6)	33(2)
C(32)	3287(5)	6245(4)	2402(5)	28(1)
C(33)	3037(5)	5541(4)	4466(5)	23(1)
C(34)	3731(6)	6134(4)	4906(5)	26(1)
C(35)	3386(6)	6641(4)	5654(5)	33(2)
C(36)	2327(7)	6596(5)	6004(6)	44(2)
C(37)	1618(7)	6029(5)	5592(6)	45(2)

C(38)	1965(6)	5513(4)	4839(5)	34(2)
C(39)	2542(5)	4325(4)	3169(5)	24(1)
C(40)	2158(6)	3735(4)	3813(5)	32(2)
C(41)	1353(6)	3178(4)	3531(6)	38(2)
C(42)	907(6)	3196(5)	2553(6)	39(2)
C(43)	1259(6)	3776(4)	1882(5)	33(2)
C(44)	2065(5)	4325(4)	2195(5)	27(1)
C(45)	4188(5)	10604(3)	6479(5)	24(1)
C(46)	4848(5)	10623(4)	7358(5)	30(2)
C(47)	5691(6)	11188(4)	7514(6)	36(2)
C(48)	5874(6)	11758(4)	6792(7)	47(2)
C(49)	5220(7)	11766(4)	5899(7)	45(2)
C(50)	4402(7)	11207(4)	5760(6)	36(2)
C(51)	2055(5)	10448(4)	5952(5)	23(1)
C(52)	1848(6)	11173(4)	6454(5)	30(2)
C(53)	877(6)	11608(4)	6300(6)	37(2)
C(54)	72(6)	11347(4)	5615(5)	35(2)
C(55)	238(6)	10638(4)	5100(5)	32(2)
C(56)	1209(5)	10216(4)	5277(5)	26(1)
C(57)	3555(5)	9351(4)	5312(4)	23(1)
C(58)	4524(6)	9444(4)	4774(5)	30(1)
C(59)	4784(6)	8943(5)	3970(6)	38(2)
C(60)	4091(7)	8327(5)	3684(6)	40(2)
C(61)	3132(6)	8196(4)	4213(5)	34(2)
C(62)	2891(5)	8703(4)	5024(5)	26(1)
C(63)	2992(5)	9410(4)	7265(5)	23(1)
C(64)	3487(6)	8656(4)	7436(5)	28(1)
C(65)	3303(6)	8208(4)	8297(6)	34(2)
C(66)	2632(6)	8488(4)	9034(5)	31(2)
C(67)	2141(5)	9223(4)	8908(5)	31(2)
C(68)	2313(5)	9674(4)	8042(5)	25(1)

Table S3. Bond lengths [Å] and angles [°] for **2b**.

Se(1)-C(10)	1.903(7)
Se(1)-Se(2)	2.3104(14)
Se(2)-C(20)	1.902(7)
Co(1)-C(3)	2.000(9)
Co(1)-C(2)	2.003(9)
Co(1)-C(4)	2.010(9)
Co(1)-C(1)	2.014(9)
Co(1)-C(5)	2.014(8)
Co(1)-C(6)	2.029(8)
Co(1)-C(7)	2.033(7)
Co(1)-C(8)	2.039(8)
Co(1)-C(9)	2.056(7)
Co(1)-C(10)	2.057(7)
Co(2)-C(11)	2.013(8)
Co(2)-C(15)	2.015(8)
Co(2)-C(14)	2.024(8)
Co(2)-C(13)	2.025(7)
Co(2)-C(20)	2.031(7)
Co(2)-C(17)	2.032(7)
Co(2)-C(18)	2.032(7)
Co(2)-C(12)	2.037(7)
Co(2)-C(19)	2.042(6)
Co(2)-C(16)	2.044(7)
B(1)-C(33)	1.635(9)
B(1)-C(27)	1.639(9)
B(1)-C(39)	1.655(9)

B(1)-C(21)	1.672(9)	C(23)-C(24)	1.386(10)
B(2)-C(45)	1.636(9)	C(23)-H(23)	0.9500
B(2)-C(63)	1.649(9)	C(24)-C(25)	1.369(10)
B(2)-C(51)	1.649(9)	C(24)-H(24)	0.9500
B(2)-C(57)	1.655(9)	C(25)-C(26)	1.392(10)
C(1)-C(5)	1.408(15)	C(25)-H(25)	0.9500
C(1)-C(2)	1.428(17)	C(26)-H(26)	0.9500
C(1)-H(1)	0.9500	C(27)-C(28)	1.388(9)
C(2)-C(3)	1.434(18)	C(27)-C(32)	1.396(9)
C(2)-H(2)	0.9500	C(28)-C(29)	1.390(10)
C(3)-C(4)	1.426(16)	C(28)-H(28)	0.9500
C(3)-H(3)	0.9500	C(29)-C(30)	1.387(10)
C(4)-C(5)	1.376(16)	C(29)-H(29)	0.9500
C(4)-H(4)	0.9500	C(30)-C(31)	1.373(10)
C(5)-H(5)	0.9500	C(30)-H(30)	0.9500
C(6)-C(7)	1.403(13)	C(31)-C(32)	1.383(10)
C(6)-C(10)	1.439(11)	C(31)-H(31)	0.9500
C(6)-H(6)	0.9500	C(32)-H(32)	0.9500
C(7)-C(8)	1.410(12)	C(33)-C(38)	1.395(9)
C(7)-H(7)	0.9500	C(33)-C(34)	1.411(9)
C(8)-C(9)	1.413(11)	C(34)-C(35)	1.377(9)
C(8)-H(8)	0.9500	C(34)-H(34)	0.9500
C(9)-C(10)	1.431(11)	C(35)-C(36)	1.370(11)
C(9)-H(9)	0.9500	C(35)-H(35)	0.9500
C(11)-C(12)	1.399(12)	C(36)-C(37)	1.377(13)
C(11)-C(15)	1.405(14)	C(36)-H(36)	0.9500
C(11)-H(11)	0.9500	C(37)-C(38)	1.391(11)
C(12)-C(13)	1.412(12)	C(37)-H(37)	0.9500
C(12)-H(12)	0.9500	C(38)-H(38)	0.9500
C(13)-C(14)	1.397(12)	C(39)-C(40)	1.391(9)
C(13)-H(13)	0.9500	C(39)-C(44)	1.393(9)
C(14)-C(15)	1.402(15)	C(40)-C(41)	1.389(10)
C(14)-H(14)	0.9500	C(40)-H(40)	0.9500
C(15)-H(15)	0.9500	C(41)-C(42)	1.384(12)
C(16)-C(17)	1.412(10)	C(41)-H(41)	0.9500
C(16)-C(20)	1.442(10)	C(42)-C(43)	1.389(11)
C(16)-H(16)	0.9500	C(42)-H(42)	0.9500
C(17)-C(18)	1.434(10)	C(43)-C(44)	1.390(9)
C(17)-H(17)	0.9500	C(43)-H(43)	0.9500
C(18)-C(19)	1.411(11)	C(44)-H(44)	0.9500
C(18)-H(18)	0.9500	C(45)-C(46)	1.387(9)
C(19)-C(20)	1.419(10)	C(45)-C(50)	1.415(9)
C(19)-H(19)	0.9500	C(46)-C(47)	1.398(10)
C(21)-C(26)	1.390(9)	C(46)-H(46)	0.9500
C(21)-C(22)	1.401(9)	C(47)-C(48)	1.373(12)
C(22)-C(23)	1.401(9)	C(47)-H(47)	0.9500
C(22)-H(22)	0.9500	C(48)-C(49)	1.398(13)

C(48)-H(48)	0.9500	C(2)-Co(1)-C(5)	69.2(5)
C(49)-C(50)	1.367(11)	C(4)-Co(1)-C(5)	40.0(5)
C(49)-H(49)	0.9500	C(1)-Co(1)-C(5)	40.9(4)
C(50)-H(50)	0.9500	C(3)-Co(1)-C(6)	160.2(4)
C(51)-C(56)	1.388(9)	C(2)-Co(1)-C(6)	122.6(4)
C(51)-C(52)	1.410(9)	C(4)-Co(1)-C(6)	156.5(5)
C(52)-C(53)	1.386(10)	C(1)-Co(1)-C(6)	105.9(4)
C(52)-H(52)	0.9500	C(5)-Co(1)-C(6)	121.3(4)
C(53)-C(54)	1.377(11)	C(3)-Co(1)-C(7)	123.8(4)
C(53)-H(53)	0.9500	C(2)-Co(1)-C(7)	106.7(4)
C(54)-C(55)	1.386(10)	C(4)-Co(1)-C(7)	161.9(5)
C(54)-H(54)	0.9500	C(1)-Co(1)-C(7)	120.8(4)
C(55)-C(56)	1.378(10)	C(5)-Co(1)-C(7)	156.8(4)
C(55)-H(55)	0.9500	C(6)-Co(1)-C(7)	40.4(4)
C(56)-H(56)	0.9500	C(3)-Co(1)-C(8)	107.0(4)
C(57)-C(58)	1.391(9)	C(2)-Co(1)-C(8)	121.3(5)
C(57)-C(62)	1.394(9)	C(4)-Co(1)-C(8)	125.2(5)
C(58)-C(59)	1.396(10)	C(1)-Co(1)-C(8)	157.0(4)
C(58)-H(58)	0.9500	C(5)-Co(1)-C(8)	161.1(4)
C(59)-C(60)	1.373(11)	C(6)-Co(1)-C(8)	68.4(4)
C(59)-H(59)	0.9500	C(7)-Co(1)-C(8)	40.5(4)
C(60)-C(61)	1.383(11)	C(3)-Co(1)-C(9)	121.0(5)
C(60)-H(60)	0.9500	C(2)-Co(1)-C(9)	157.1(5)
C(61)-C(62)	1.404(10)	C(4)-Co(1)-C(9)	108.0(4)
C(61)-H(61)	0.9500	C(1)-Co(1)-C(9)	160.4(4)
C(62)-H(62)	0.9500	C(5)-Co(1)-C(9)	124.5(4)
C(63)-C(68)	1.403(9)	C(6)-Co(1)-C(9)	68.8(3)
C(63)-C(64)	1.411(9)	C(7)-Co(1)-C(9)	68.1(3)
C(64)-C(65)	1.387(10)	C(8)-Co(1)-C(9)	40.4(3)
C(64)-H(64)	0.9500	C(3)-Co(1)-C(10)	156.8(5)
C(65)-C(66)	1.367(11)	C(2)-Co(1)-C(10)	160.2(5)
C(65)-H(65)	0.9500	C(4)-Co(1)-C(10)	121.2(4)
C(66)-C(67)	1.374(10)	C(1)-Co(1)-C(10)	123.1(4)
C(66)-H(66)	0.9500	C(5)-Co(1)-C(10)	107.4(4)
C(67)-C(68)	1.392(9)	C(6)-Co(1)-C(10)	41.2(3)
C(67)-H(67)	0.9500	C(7)-Co(1)-C(10)	68.3(3)
C(68)-H(68)	0.9500	C(8)-Co(1)-C(10)	68.2(3)
		C(9)-Co(1)-C(10)	40.7(3)
C(10)-Se(1)-Se(2)	101.0(2)	C(11)-Co(2)-C(15)	40.8(4)
C(20)-Se(2)-Se(1)	100.4(2)	C(11)-Co(2)-C(14)	68.3(4)
C(3)-Co(1)-C(2)	42.0(5)	C(15)-Co(2)-C(14)	40.6(4)
C(3)-Co(1)-C(4)	41.7(5)	C(11)-Co(2)-C(13)	68.2(4)
C(2)-Co(1)-C(4)	69.6(5)	C(15)-Co(2)-C(13)	68.3(4)
C(3)-Co(1)-C(1)	70.4(5)	C(14)-Co(2)-C(13)	40.4(3)
C(2)-Co(1)-C(1)	41.7(5)	C(11)-Co(2)-C(20)	127.7(3)
C(4)-Co(1)-C(1)	68.9(5)	C(15)-Co(2)-C(20)	108.3(3)
C(3)-Co(1)-C(5)	69.3(5)	C(14)-Co(2)-C(20)	119.4(3)

C(13)-Co(2)-C(20)	153.0(3)
C(11)-Co(2)-C(17)	117.9(4)
C(15)-Co(2)-C(17)	152.5(4)
C(14)-Co(2)-C(17)	164.9(4)
C(13)-Co(2)-C(17)	126.8(3)
C(20)-Co(2)-C(17)	69.0(3)
C(11)-Co(2)-C(18)	152.5(4)
C(15)-Co(2)-C(18)	165.2(4)
C(14)-Co(2)-C(18)	127.2(4)
C(13)-Co(2)-C(18)	107.6(3)
C(20)-Co(2)-C(18)	68.5(3)
C(17)-Co(2)-C(18)	41.3(3)
C(11)-Co(2)-C(12)	40.4(3)
C(15)-Co(2)-C(12)	68.3(3)
C(14)-Co(2)-C(12)	68.2(3)
C(13)-Co(2)-C(12)	40.7(3)
C(20)-Co(2)-C(12)	165.1(3)
C(17)-Co(2)-C(12)	106.9(3)
C(18)-Co(2)-C(12)	118.5(3)
C(11)-Co(2)-C(19)	165.8(3)
C(15)-Co(2)-C(19)	127.7(4)
C(14)-Co(2)-C(19)	108.3(3)
C(13)-Co(2)-C(19)	118.8(3)
C(20)-Co(2)-C(19)	40.8(3)
C(17)-Co(2)-C(19)	69.1(3)
C(18)-Co(2)-C(19)	40.5(3)
C(12)-Co(2)-C(19)	152.6(3)
C(11)-Co(2)-C(16)	107.5(4)
C(15)-Co(2)-C(16)	119.1(4)
C(14)-Co(2)-C(16)	153.8(3)
C(13)-Co(2)-C(16)	164.2(3)
C(20)-Co(2)-C(16)	41.4(3)
C(17)-Co(2)-C(16)	40.5(3)
C(18)-Co(2)-C(16)	68.8(3)
C(12)-Co(2)-C(16)	126.4(3)
C(19)-Co(2)-C(16)	69.1(3)
C(33)-B(1)-C(27)	108.0(5)
C(33)-B(1)-C(39)	111.4(5)
C(27)-B(1)-C(39)	110.7(5)
C(33)-B(1)-C(21)	111.1(5)
C(27)-B(1)-C(21)	110.8(5)
C(39)-B(1)-C(21)	104.9(5)
C(45)-B(2)-C(63)	110.0(5)
C(45)-B(2)-C(51)	107.5(5)
C(63)-B(2)-C(51)	108.8(5)
C(45)-B(2)-C(57)	110.3(5)
C(63)-B(2)-C(57)	108.9(5)

C(51)-B(2)-C(57)	111.2(5)
C(5)-C(1)-C(2)	107.0(10)
C(5)-C(1)-Co(1)	69.5(5)
C(2)-C(1)-Co(1)	68.8(5)
C(5)-C(1)-H(1)	126.5
C(2)-C(1)-H(1)	126.5
Co(1)-C(1)-H(1)	126.7
C(1)-C(2)-C(3)	107.9(9)
C(1)-C(2)-Co(1)	69.6(5)
C(3)-C(2)-Co(1)	68.9(5)
C(1)-C(2)-H(2)	126.0
C(3)-C(2)-H(2)	126.0
Co(1)-C(2)-H(2)	127.1
C(4)-C(3)-C(2)	106.4(11)
C(4)-C(3)-Co(1)	69.5(5)
C(2)-C(3)-Co(1)	69.1(6)
C(4)-C(3)-H(3)	126.8
C(2)-C(3)-H(3)	126.8
Co(1)-C(3)-H(3)	126.1
C(5)-C(4)-C(3)	109.0(11)
C(5)-C(4)-Co(1)	70.1(5)
C(3)-C(4)-Co(1)	68.8(6)
C(5)-C(4)-H(4)	125.5
C(3)-C(4)-H(4)	125.5
Co(1)-C(4)-H(4)	127.1
C(4)-C(5)-C(1)	109.7(10)
C(4)-C(5)-Co(1)	69.9(6)
C(1)-C(5)-Co(1)	69.5(5)
C(4)-C(5)-H(5)	125.2
C(1)-C(5)-H(5)	125.2
Co(1)-C(5)-H(5)	127.1
C(7)-C(6)-C(10)	107.8(7)
C(7)-C(6)-Co(1)	69.9(5)
C(10)-C(6)-Co(1)	70.4(4)
C(7)-C(6)-H(6)	126.1
C(10)-C(6)-H(6)	126.1
Co(1)-C(6)-H(6)	125.2
C(6)-C(7)-C(8)	108.7(7)
C(6)-C(7)-Co(1)	69.6(4)
C(8)-C(7)-Co(1)	70.0(4)
C(6)-C(7)-H(7)	125.6
C(8)-C(7)-H(7)	125.6
Co(1)-C(7)-H(7)	126.4
C(7)-C(8)-C(9)	108.5(8)
C(7)-C(8)-Co(1)	69.5(5)
C(9)-C(8)-Co(1)	70.5(4)
C(7)-C(8)-H(8)	125.8

C(9)-C(8)-H(8)	125.8
Co(1)-C(8)-H(8)	125.8
C(8)-C(9)-C(10)	107.8(7)
C(8)-C(9)-Co(1)	69.2(4)
C(10)-C(9)-Co(1)	69.6(4)
C(8)-C(9)-H(9)	126.1
C(10)-C(9)-H(9)	126.1
Co(1)-C(9)-H(9)	126.6
C(9)-C(10)-C(6)	107.2(7)
C(9)-C(10)-Se(1)	129.4(6)
C(6)-C(10)-Se(1)	123.3(6)
C(9)-C(10)-Co(1)	69.6(4)
C(6)-C(10)-Co(1)	68.4(4)
Se(1)-C(10)-Co(1)	129.4(4)
C(12)-C(11)-C(15)	108.5(8)
C(12)-C(11)-Co(2)	70.7(4)
C(15)-C(11)-Co(2)	69.7(5)
C(12)-C(11)-H(11)	125.7
C(15)-C(11)-H(11)	125.7
Co(2)-C(11)-H(11)	125.5
C(11)-C(12)-C(13)	107.4(8)
C(11)-C(12)-Co(2)	68.9(4)
C(13)-C(12)-Co(2)	69.2(4)
C(11)-C(12)-H(12)	126.3
C(13)-C(12)-H(12)	126.3
Co(2)-C(12)-H(12)	127.1
C(14)-C(13)-C(12)	108.2(8)
C(14)-C(13)-Co(2)	69.8(5)
C(12)-C(13)-Co(2)	70.1(4)
C(14)-C(13)-H(13)	125.9
C(12)-C(13)-H(13)	125.9
Co(2)-C(13)-H(13)	125.8
C(13)-C(14)-C(15)	108.2(8)
C(13)-C(14)-Co(2)	69.8(4)
C(15)-C(14)-Co(2)	69.4(5)
C(13)-C(14)-H(14)	125.9
C(15)-C(14)-H(14)	125.9
Co(2)-C(14)-H(14)	126.5
C(14)-C(15)-C(11)	107.7(8)
C(14)-C(15)-Co(2)	70.0(5)
C(11)-C(15)-Co(2)	69.5(5)
C(14)-C(15)-H(15)	126.2
C(11)-C(15)-H(15)	126.2
Co(2)-C(15)-H(15)	125.9
C(17)-C(16)-C(20)	107.5(6)
C(17)-C(16)-Co(2)	69.3(4)
C(20)-C(16)-Co(2)	68.8(4)
C(17)-C(16)-H(16)	126.2
C(20)-C(16)-H(16)	126.2
Co(2)-C(16)-H(16)	127.3
C(16)-C(17)-C(18)	107.9(6)
C(16)-C(17)-Co(2)	70.2(4)
C(18)-C(17)-Co(2)	69.3(4)
C(16)-C(17)-H(17)	126.0
C(18)-C(17)-H(17)	126.0
Co(2)-C(17)-H(17)	126.0
C(19)-C(18)-C(17)	108.5(6)
C(19)-C(18)-Co(2)	70.1(4)
C(17)-C(18)-Co(2)	69.3(4)
C(19)-C(18)-H(18)	125.7
C(17)-C(18)-H(18)	125.7
Co(2)-C(18)-H(18)	126.4
C(18)-C(19)-C(20)	107.9(6)
C(18)-C(19)-Co(2)	69.4(4)
C(20)-C(19)-Co(2)	69.2(4)
C(18)-C(19)-H(19)	126.1
C(20)-C(19)-H(19)	126.1
Co(2)-C(19)-H(19)	126.9
C(19)-C(20)-C(16)	108.1(6)
C(19)-C(20)-Se(2)	130.1(5)
C(16)-C(20)-Se(2)	121.6(5)
C(19)-C(20)-Co(2)	70.0(4)
C(16)-C(20)-Co(2)	69.8(4)
Se(2)-C(20)-Co(2)	128.8(4)
C(26)-C(21)-C(22)	115.4(6)
C(26)-C(21)-B(1)	120.5(6)
C(22)-C(21)-B(1)	124.1(5)
C(21)-C(22)-C(23)	122.4(6)
C(21)-C(22)-H(22)	118.8
C(23)-C(22)-H(22)	118.8
C(24)-C(23)-C(22)	119.7(6)
C(24)-C(23)-H(23)	120.2
C(22)-C(23)-H(23)	120.2
C(25)-C(24)-C(23)	119.1(6)
C(25)-C(24)-H(24)	120.4
C(23)-C(24)-H(24)	120.4
C(24)-C(25)-C(26)	120.4(6)
C(24)-C(25)-H(25)	119.8
C(26)-C(25)-H(25)	119.8
C(21)-C(26)-C(25)	122.8(6)
C(21)-C(26)-H(26)	118.6
C(25)-C(26)-H(26)	118.6
C(28)-C(27)-C(32)	114.4(6)
C(28)-C(27)-B(1)	125.0(5)

C(32)-C(27)-B(1)	120.6(5)
C(27)-C(28)-C(29)	123.8(6)
C(27)-C(28)-H(28)	118.1
C(29)-C(28)-H(28)	118.1
C(30)-C(29)-C(28)	119.2(6)
C(30)-C(29)-H(29)	120.4
C(28)-C(29)-H(29)	120.4
C(31)-C(30)-C(29)	119.1(6)
C(31)-C(30)-H(30)	120.4
C(29)-C(30)-H(30)	120.4
C(30)-C(31)-C(32)	120.0(6)
C(30)-C(31)-H(31)	120.0
C(32)-C(31)-H(31)	120.0
C(31)-C(32)-C(27)	123.4(6)
C(31)-C(32)-H(32)	118.3
C(27)-C(32)-H(32)	118.3
C(38)-C(33)-C(34)	114.8(6)
C(38)-C(33)-B(1)	125.1(6)
C(34)-C(33)-B(1)	120.1(5)
C(35)-C(34)-C(33)	122.9(6)
C(35)-C(34)-H(34)	118.5
C(33)-C(34)-H(34)	118.5
C(36)-C(35)-C(34)	120.7(7)
C(36)-C(35)-H(35)	119.7
C(34)-C(35)-H(35)	119.7
C(35)-C(36)-C(37)	118.4(7)
C(35)-C(36)-H(36)	120.8
C(37)-C(36)-H(36)	120.8
C(36)-C(37)-C(38)	121.1(7)
C(36)-C(37)-H(37)	119.5
C(38)-C(37)-H(37)	119.5
C(37)-C(38)-C(33)	122.1(7)
C(37)-C(38)-H(38)	118.9
C(33)-C(38)-H(38)	118.9
C(40)-C(39)-C(44)	115.5(6)
C(40)-C(39)-B(1)	120.7(6)
C(44)-C(39)-B(1)	123.8(6)
C(41)-C(40)-C(39)	123.8(7)
C(41)-C(40)-H(40)	118.1
C(39)-C(40)-H(40)	118.1
C(42)-C(41)-C(40)	118.8(7)
C(42)-C(41)-H(41)	120.6
C(40)-C(41)-H(41)	120.6
C(41)-C(42)-C(43)	119.6(6)
C(41)-C(42)-H(42)	120.2
C(43)-C(42)-H(42)	120.2
C(42)-C(43)-C(44)	119.8(7)
C(42)-C(43)-H(43)	120.1
C(44)-C(43)-H(43)	120.1
C(43)-C(44)-C(39)	122.5(6)
C(43)-C(44)-H(44)	118.7
C(39)-C(44)-H(44)	118.7
C(46)-C(45)-C(50)	115.8(6)
C(46)-C(45)-B(2)	124.4(6)
C(50)-C(45)-B(2)	119.8(6)
C(45)-C(46)-C(47)	122.2(7)
C(45)-C(46)-H(46)	118.9
C(47)-C(46)-H(46)	118.9
C(48)-C(47)-C(46)	120.0(7)
C(48)-C(47)-H(47)	120.0
C(46)-C(47)-H(47)	120.0
C(47)-C(48)-C(49)	119.7(7)
C(47)-C(48)-H(48)	120.2
C(49)-C(48)-H(48)	120.2
C(50)-C(49)-C(48)	119.4(7)
C(50)-C(49)-H(49)	120.3
C(48)-C(49)-H(49)	120.3
C(49)-C(50)-C(45)	122.9(8)
C(49)-C(50)-H(50)	118.5
C(45)-C(50)-H(50)	118.5
C(56)-C(51)-C(52)	114.0(6)
C(56)-C(51)-B(2)	127.0(6)
C(52)-C(51)-B(2)	118.9(6)
C(53)-C(52)-C(51)	122.9(6)
C(53)-C(52)-H(52)	118.6
C(51)-C(52)-H(52)	118.6
C(54)-C(53)-C(52)	120.2(6)
C(54)-C(53)-H(53)	119.9
C(52)-C(53)-H(53)	119.9
C(53)-C(54)-C(55)	119.1(6)
C(53)-C(54)-H(54)	120.5
C(55)-C(54)-H(54)	120.5
C(56)-C(55)-C(54)	119.3(6)
C(56)-C(55)-H(55)	120.3
C(54)-C(55)-H(55)	120.3
C(55)-C(56)-C(51)	124.5(6)
C(55)-C(56)-H(56)	117.7
C(51)-C(56)-H(56)	117.7
C(58)-C(57)-C(62)	115.4(6)
C(58)-C(57)-B(2)	123.4(6)
C(62)-C(57)-B(2)	121.3(5)
C(57)-C(58)-C(59)	122.1(7)
C(57)-C(58)-H(58)	118.9
C(59)-C(58)-H(58)	118.9

C(60)-C(59)-C(58)	120.9(7)
C(60)-C(59)-H(59)	119.6
C(58)-C(59)-H(59)	119.6
C(59)-C(60)-C(61)	119.2(7)
C(59)-C(60)-H(60)	120.4
C(61)-C(60)-H(60)	120.4
C(60)-C(61)-C(62)	119.0(7)
C(60)-C(61)-H(61)	120.5
C(62)-C(61)-H(61)	120.5
C(57)-C(62)-C(61)	123.3(6)
C(57)-C(62)-H(62)	118.3
C(61)-C(62)-H(62)	118.3
C(68)-C(63)-C(64)	114.7(6)
C(68)-C(63)-B(2)	122.1(5)
C(64)-C(63)-B(2)	123.2(6)
C(65)-C(64)-C(63)	122.6(6)
C(65)-C(64)-H(64)	118.7
C(63)-C(64)-H(64)	118.7
C(66)-C(65)-C(64)	120.7(6)
C(66)-C(65)-H(65)	119.6
C(64)-C(65)-H(65)	119.6
C(65)-C(66)-C(67)	118.8(6)
C(65)-C(66)-H(66)	120.6
C(67)-C(66)-H(66)	120.6
C(66)-C(67)-C(68)	120.8(6)
C(66)-C(67)-H(67)	119.6
C(68)-C(67)-H(67)	119.6
C(67)-C(68)-C(63)	122.3(6)
C(67)-C(68)-H(68)	118.8
C(63)-C(68)-H(68)	118.8

Symmetry transformations used to generate equivalent atoms:

Table S4. Anisotropic displacement parameters (Å^2 x 10^3) for **2b**. The anisotropic displacement factor exponent takes the form: $-2\pi^2 [h^2 a^{*2} U^{11} + ... + 2 h k a^* b^* U^{12}]$

	U^{11}	U^{22}	U^{33}	U^{23}	U^{13}	U^{12}
Se(1)	78(1)	54(1)	42(1)	-14(1)	-17(1)	18(1)
Se(2)	64(1)	46(1)	33(1)	-12(1)	15(1)	-15(1)
Co(1)	34(1)	36(1)	28(1)	-4(1)	7(1)	-13(1)
Co(2)	30(1)	23(1)	21(1)	-1(1)	-1(1)	0(1)
B(1)	17(3)	27(3)	21(3)	0(3)	7(3)	0(3)
B(2)	21(3)	23(3)	22(3)	-1(3)	2(3)	-1(3)
C(1)	60(6)	109(9)	22(4)	0(5)	15(4)	-9(6)
C(2)	101(9)	101(9)	47(6)	-47(6)	31(6)	-36(7)
C(3)	98(9)	55(6)	74(7)	-15(5)	54(7)	11(6)
C(4)	39(5)	125(10)	58(6)	-16(6)	27(5)	-12(6)
C(5)	77(7)	69(6)	51(6)	5(5)	34(5)	-25(6)
C(6)	28(4)	70(6)	35(4)	-8(4)	2(3)	-5(4)
C(7)	47(5)	53(5)	43(5)	-5(4)	15(4)	-25(4)
C(8)	61(5)	49(5)	37(4)	4(4)	18(4)	-7(4)
C(9)	47(4)	47(4)	22(3)	-8(3)	1(3)	0(3)
C(10)	37(4)	47(4)	29(4)	-4(3)	7(3)	1(4)
C(11)	78(6)	25(4)	64(6)	6(4)	20(5)	10(4)
C(12)	51(5)	36(4)	39(4)	-15(3)	2(4)	10(3)
C(13)	33(4)	47(4)	45(4)	-6(4)	4(3)	12(3)
C(14)	47(5)	64(6)	52(5)	-17(5)	-22(4)	28(4)
C(15)	96(7)	59(6)	30(4)	13(4)	5(5)	48(5)
C(16)	28(4)	38(4)	32(4)	0(3)	0(3)	-5(3)
C(17)	34(4)	33(4)	34(4)	2(3)	-9(3)	5(3)
C(18)	35(4)	38(4)	35(4)	16(3)	2(3)	4(3)
C(19)	32(4)	19(3)	45(4)	-6(3)	5(3)	-1(3)
C(20)	37(4)	26(3)	28(4)	-2(3)	3(3)	-4(3)

C(21)	20(3)	21(3)	24(3)	4(2)	6(2)	-5(2)
C(22)	29(3)	27(3)	26(3)	-3(3)	4(3)	-1(3)
C(23)	33(4)	32(4)	29(4)	7(3)	-4(3)	2(3)
C(24)	32(4)	21(3)	41(4)	5(3)	1(3)	1(3)
C(25)	37(4)	19(3)	33(4)	3(3)	9(3)	0(3)
C(26)	33(3)	21(3)	32(4)	-3(3)	1(3)	-4(3)
C(27)	19(3)	21(3)	23(3)	-1(2)	0(2)	-4(2)
C(28)	38(4)	28(3)	32(4)	6(3)	15(3)	5(3)
C(29)	31(4)	39(4)	34(4)	2(3)	16(3)	4(3)
C(30)	39(4)	31(4)	28(3)	1(3)	6(3)	-11(3)
C(31)	36(4)	25(3)	39(4)	4(3)	3(3)	2(3)
C(32)	26(3)	35(3)	24(3)	-2(3)	8(3)	2(3)
C(33)	28(3)	20(3)	22(3)	3(2)	1(3)	1(2)
C(34)	30(3)	22(3)	28(3)	-1(3)	4(3)	3(3)
C(35)	43(4)	25(3)	32(4)	-4(3)	-5(3)	4(3)
C(36)	67(5)	36(4)	28(4)	1(3)	11(4)	18(4)
C(37)	40(4)	51(5)	44(4)	3(4)	19(3)	10(4)
C(38)	29(4)	40(4)	32(4)	0(3)	11(3)	-2(3)
C(39)	20(3)	30(3)	24(3)	-3(3)	7(2)	1(3)
C(40)	34(4)	34(4)	27(4)	-1(3)	4(3)	-6(3)
C(41)	34(4)	34(4)	47(4)	0(3)	12(3)	-11(3)
C(42)	27(3)	43(4)	48(5)	-15(3)	5(3)	-11(3)
C(43)	27(3)	40(4)	31(4)	-10(3)	-5(3)	2(3)
C(44)	24(3)	30(3)	27(3)	-3(3)	7(3)	2(3)
C(45)	29(3)	19(3)	25(3)	-2(2)	8(3)	-4(2)
C(46)	24(3)	31(3)	36(4)	-1(3)	2(3)	3(3)
C(47)	24(3)	38(4)	46(4)	-17(3)	-1(3)	-3(3)
C(48)	32(4)	34(4)	75(6)	-18(4)	21(4)	-15(3)
C(49)	57(5)	28(4)	51(5)	-3(3)	29(4)	-13(3)
C(50)	51(4)	27(3)	33(4)	3(3)	10(3)	-7(3)
C(51)	30(3)	19(3)	21(3)	3(2)	4(3)	0(2)
C(52)	37(4)	27(3)	28(3)	-4(3)	-6(3)	2(3)
C(53)	47(4)	27(3)	36(4)	-6(3)	0(3)	11(3)
C(54)	34(4)	39(4)	32(4)	2(3)	1(3)	11(3)
C(55)	31(4)	35(4)	30(4)	0(3)	-3(3)	-3(3)
C(56)	26(3)	29(3)	24(3)	-1(3)	5(3)	0(3)
C(57)	28(3)	26(3)	16(3)	-1(2)	-4(2)	6(3)
C(58)	29(3)	31(3)	29(3)	-4(3)	5(3)	3(3)
C(59)	34(4)	50(4)	30(4)	-4(3)	10(3)	6(3)
C(60)	55(5)	37(4)	29(4)	-5(3)	5(3)	12(3)
C(61)	44(4)	28(3)	29(4)	-2(3)	-5(3)	3(3)
C(62)	31(3)	22(3)	26(3)	3(3)	4(3)	2(3)
C(63)	23(3)	24(3)	22(3)	-1(2)	-2(2)	-6(2)
C(64)	36(4)	21(3)	29(4)	2(3)	5(3)	3(3)
C(65)	45(4)	23(3)	33(4)	7(3)	-2(3)	-1(3)
C(66)	35(4)	36(4)	21(3)	6(3)	-1(3)	-15(3)
C(67)	27(3)	45(4)	22(3)	-4(3)	2(3)	-7(3)

C(68)	26(3)	23(3)	25(3)	-4(3)	2(3)	-1(2)

Table S5. Hydrogen coordinates (x 10^4) and isotropic displacement parameters (Å^2 x 10^3) for **2b**.

	x	y	z	U(eq)
H(1)	7650	6373	1387	76
H(2)	8138	4927	1814	99
H(3)	9893	4939	2964	90
H(4)	10477	6392	3166	88
H(5)	9119	7243	2224	78
H(6)	6183	6516	3350	53
H(7)	6568	5073	3756	57
H(8)	8291	4994	4884	59
H(9)	8972	6397	5235	46
H(11)	8607	10449	6939	66
H(12)	7875	9959	8592	50
H(13)	6429	8899	8189	50
H(14)	6232	8775	6298	66
H(15)	7584	9727	5519	74
H(16)	10497	9050	6702	39
H(17)	9999	8734	8508	41
H(18)	8517	7648	8455	44
H(19)	8076	7305	6625	38
H(22)	4787	4867	5408	33
H(23)	6306	4083	5929	38
H(24)	7107	3200	4781	38
H(25)	6254	3012	3200	36
H(26)	4678	3735	2724	34
H(28)	5210	4943	2150	39
H(29)	5623	5734	772	41
H(30)	4537	6870	406	39
H(31)	3100	7204	1471	40
H(32)	2689	6390	2818	34
H(34)	4468	6186	4675	32
H(35)	3889	7027	5930	40
H(36)	2087	6946	6519	52
H(37)	880	5990	5825	53
H(38)	1455	5129	4571	40
H(40)	2466	3713	4483	38
H(41)	1111	2790	4002	46
H(42)	364	2815	2342	47
H(43)	950	3796	1213	39
H(44)	2300	4716	1726	33
H(46)	4723	10239	7871	36
H(47)	6138	11177	8120	43
H(48)	6443	12145	6898	56
H(49)	5343	12156	5393	54

H(50)	3957	11223	5154	44
H(52)	2399	11372	6919	37
H(53)	767	12087	6667	44
H(54)	-589	11648	5498	42
H(55)	-311	10445	4629	39
H(56)	1305	9734	4912	31
H(58)	5024	9861	4959	36
H(59)	5450	9029	3617	46
H(60)	4267	7994	3129	48
H(61)	2645	7770	4031	40
H(62)	2242	8599	5395	32
H(64)	3966	8448	6941	34
H(65)	3647	7700	8375	40
H(66)	2507	8180	9624	37
H(67)	1678	9426	9419	37
H(68)	1958	10179	7975	30

Table S6. Torsion angles [°] for **2b**.

C(5)-C(1)-C(2)-C(3)	0.9(10)
Co(1)-C(1)-C(2)-C(3)	-58.4(6)
C(5)-C(1)-C(2)-Co(1)	59.3(6)
C(1)-C(2)-C(3)-C(4)	-1.1(10)
Co(1)-C(2)-C(3)-C(4)	-59.9(6)
C(1)-C(2)-C(3)-Co(1)	58.8(6)
C(2)-C(3)-C(4)-C(5)	0.8(11)
Co(1)-C(3)-C(4)-C(5)	-58.7(7)
C(2)-C(3)-C(4)-Co(1)	59.6(6)
C(3)-C(4)-C(5)-C(1)	-0.3(11)
Co(1)-C(4)-C(5)-C(1)	-58.2(6)
C(3)-C(4)-C(5)-Co(1)	57.9(7)
C(2)-C(1)-C(5)-C(4)	-0.4(10)
Co(1)-C(1)-C(5)-C(4)	58.4(6)
C(2)-C(1)-C(5)-Co(1)	-58.8(6)
C(10)-C(6)-C(7)-C(8)	-1.3(9)
Co(1)-C(6)-C(7)-C(8)	59.2(5)
C(10)-C(6)-C(7)-Co(1)	-60.5(5)
C(6)-C(7)-C(8)-C(9)	0.9(9)
Co(1)-C(7)-C(8)-C(9)	59.9(5)
C(6)-C(7)-C(8)-Co(1)	-59.0(5)
C(7)-C(8)-C(9)-C(10)	-0.2(8)
Co(1)-C(8)-C(9)-C(10)	59.1(5)
C(7)-C(8)-C(9)-Co(1)	-59.3(5)
C(8)-C(9)-C(10)-C(6)	-0.6(8)
Co(1)-C(9)-C(10)-C(6)	58.2(5)
C(8)-C(9)-C(10)-Se(1)	176.4(6)
Co(1)-C(9)-C(10)-Se(1)	-124.8(6)
C(8)-C(9)-C(10)-Co(1)	-58.8(5)
C(7)-C(6)-C(10)-C(9)	1.2(9)
Co(1)-C(6)-C(10)-C(9)	-59.0(5)
C(7)-C(6)-C(10)-Se(1)	-176.0(5)
Co(1)-C(6)-C(10)-Se(1)	123.8(6)
C(7)-C(6)-C(10)-Co(1)	60.2(5)
C(15)-C(11)-C(12)-C(13)	0.9(9)
Co(2)-C(11)-C(12)-C(13)	-58.7(5)
C(15)-C(11)-C(12)-Co(2)	59.6(6)
C(11)-C(12)-C(13)-C(14)	-1.1(9)
Co(2)-C(12)-C(13)-C(14)	-59.6(5)
C(11)-C(12)-C(13)-Co(2)	58.5(6)
C(12)-C(13)-C(14)-C(15)	0.9(9)
Co(2)-C(13)-C(14)-C(15)	-59.0(6)
C(12)-C(13)-C(14)-Co(2)	59.8(5)
C(13)-C(14)-C(15)-C(11)	-0.3(9)
Co(2)-C(14)-C(15)-C(11)	-59.5(6)
C(13)-C(14)-C(15)-Co(2)	59.3(5)
C(12)-C(11)-C(15)-C(14)	-0.4(9)
Co(2)-C(11)-C(15)-C(14)	59.9(6)
C(12)-C(11)-C(15)-Co(2)	-60.3(6)
C(20)-C(16)-C(17)-C(18)	1.0(8)
Co(2)-C(16)-C(17)-C(18)	59.3(5)
C(20)-C(16)-C(17)-Co(2)	-58.3(5)
C(16)-C(17)-C(18)-C(19)	-0.5(8)
Co(2)-C(17)-C(18)-C(19)	59.3(5)
C(16)-C(17)-C(18)-Co(2)	-59.8(5)
C(17)-C(18)-C(19)-C(20)	-0.2(8)
Co(2)-C(18)-C(19)-C(20)	58.7(5)

C(17)-C(18)-C(19)-Co(2)	-58.9(5)		C(33)-C(34)-C(35)-C(36)	-0.7(10)
C(18)-C(19)-C(20)-C(16)	0.8(8)		C(34)-C(35)-C(36)-C(37)	0.2(11)
Co(2)-C(19)-C(20)-C(16)	59.6(5)		C(35)-C(36)-C(37)-C(38)	0.1(12)
C(18)-C(19)-C(20)-Se(2)	176.8(5)		C(36)-C(37)-C(38)-C(33)	0.0(12)
Co(2)-C(19)-C(20)-Se(2)	-124.4(6)		C(34)-C(33)-C(38)-C(37)	-0.4(10)
C(18)-C(19)-C(20)-Co(2)	-58.8(5)		B(1)-C(33)-C(38)-C(37)	-178.5(7)
C(17)-C(16)-C(20)-C(19)	-1.1(8)		C(33)-B(1)-C(39)-C(40)	-64.7(7)
Co(2)-C(16)-C(20)-C(19)	-59.7(5)		C(27)-B(1)-C(39)-C(40)	175.1(6)
C(17)-C(16)-C(20)-Se(2)	-177.6(5)		C(21)-B(1)-C(39)-C(40)	55.5(7)
Co(2)-C(16)-C(20)-Se(2)	123.8(5)		C(33)-B(1)-C(39)-C(44)	115.2(6)
C(17)-C(16)-C(20)-Co(2)	58.6(5)		C(27)-B(1)-C(39)-C(44)	-5.0(8)
C(33)-B(1)-C(21)-C(26)	174.4(5)		C(21)-B(1)-C(39)-C(44)	-124.5(6)
C(27)-B(1)-C(21)-C(26)	-65.5(7)		C(44)-C(39)-C(40)-C(41)	-0.4(10)
C(39)-B(1)-C(21)-C(26)	53.9(7)		B(1)-C(39)-C(40)-C(41)	179.6(6)
C(33)-B(1)-C(21)-C(22)	-7.1(8)		C(39)-C(40)-C(41)-C(42)	0.8(11)
C(27)-B(1)-C(21)-C(22)	113.0(6)		C(40)-C(41)-C(42)-C(43)	-1.0(11)
C(39)-B(1)-C(21)-C(22)	-127.5(6)		C(41)-C(42)-C(43)-C(44)	0.8(10)
C(26)-C(21)-C(22)-C(23)	1.3(9)		C(42)-C(43)-C(44)-C(39)	-0.4(10)
B(1)-C(21)-C(22)-C(23)	-177.3(6)		C(40)-C(39)-C(44)-C(43)	0.1(9)
C(21)-C(22)-C(23)-C(24)	2.3(10)		B(1)-C(39)-C(44)-C(43)	-179.8(6)
C(22)-C(23)-C(24)-C(25)	-3.8(10)		C(63)-B(2)-C(45)-C(46)	8.3(8)
C(23)-C(24)-C(25)-C(26)	1.8(10)		C(51)-B(2)-C(45)-C(46)	126.6(6)
C(22)-C(21)-C(26)-C(25)	-3.5(9)		C(57)-B(2)-C(45)-C(46)	-111.9(7)
B(1)-C(21)-C(26)-C(25)	175.2(6)		C(63)-B(2)-C(45)-C(50)	-171.1(6)
C(24)-C(25)-C(26)-C(21)	2.0(10)		C(51)-B(2)-C(45)-C(50)	-52.8(7)
C(33)-B(1)-C(27)-C(28)	146.0(6)		C(57)-B(2)-C(45)-C(50)	68.7(7)
C(39)-B(1)-C(27)-C(28)	-91.8(7)		C(50)-C(45)-C(46)-C(47)	-1.6(9)
C(21)-B(1)-C(27)-C(28)	24.1(8)		B(2)-C(45)-C(46)-C(47)	179.0(6)
C(33)-B(1)-C(27)-C(32)	-35.5(7)		C(45)-C(46)-C(47)-C(48)	1.2(10)
C(39)-B(1)-C(27)-C(32)	86.7(7)		C(46)-C(47)-C(48)-C(49)	-0.5(11)
C(21)-B(1)-C(27)-C(32)	-157.4(6)		C(47)-C(48)-C(49)-C(50)	0.3(11)
C(32)-C(27)-C(28)-C(29)	-2.7(10)		C(48)-C(49)-C(50)-C(45)	-0.8(12)
B(1)-C(27)-C(28)-C(29)	175.9(6)		C(46)-C(45)-C(50)-C(49)	1.4(10)
C(27)-C(28)-C(29)-C(30)	1.2(11)		B(2)-C(45)-C(50)-C(49)	-179.1(7)
C(28)-C(29)-C(30)-C(31)	1.3(11)		C(45)-B(2)-C(51)-C(56)	147.1(6)
C(29)-C(30)-C(31)-C(32)	-2.0(11)		C(63)-B(2)-C(51)-C(56)	-93.8(7)
C(30)-C(31)-C(32)-C(27)	0.2(11)		C(57)-B(2)-C(51)-C(56)	26.2(8)
C(28)-C(27)-C(32)-C(31)	2.0(10)		C(45)-B(2)-C(51)-C(52)	-36.1(8)
B(1)-C(27)-C(32)-C(31)	-176.6(6)		C(63)-B(2)-C(51)-C(52)	83.1(7)
C(27)-B(1)-C(33)-C(38)	119.8(7)		C(57)-B(2)-C(51)-C(52)	-157.0(6)
C(39)-B(1)-C(33)-C(38)	-1.9(9)		C(56)-C(51)-C(52)-C(53)	1.0(10)
C(21)-B(1)-C(33)-C(38)	-118.4(7)		B(2)-C(51)-C(52)-C(53)	-176.2(6)
C(27)-B(1)-C(33)-C(34)	-58.1(7)		C(51)-C(52)-C(53)-C(54)	-1.4(11)
C(39)-B(1)-C(33)-C(34)	-179.9(5)		C(52)-C(53)-C(54)-C(55)	1.1(11)
C(21)-B(1)-C(33)-C(34)	63.7(7)		C(53)-C(54)-C(55)-C(56)	-0.6(11)
C(38)-C(33)-C(34)-C(35)	0.8(9)		C(54)-C(55)-C(56)-C(51)	0.3(10)
B(1)-C(33)-C(34)-C(35)	178.9(6)		C(52)-C(51)-C(56)-C(55)	-0.5(9)

B(2)-C(51)-C(56)-C(55)	176.5(6)
C(45)-B(2)-C(57)-C(58)	-4.1(8)
C(63)-B(2)-C(57)-C(58)	-124.9(6)
C(51)-B(2)-C(57)-C(58)	115.2(7)
C(45)-B(2)-C(57)-C(62)	175.0(6)
C(63)-B(2)-C(57)-C(62)	54.1(7)
C(51)-B(2)-C(57)-C(62)	-65.7(7)
C(62)-C(57)-C(58)-C(59)	2.8(10)
B(2)-C(57)-C(58)-C(59)	-178.1(6)
C(57)-C(58)-C(59)-C(60)	-0.6(11)
C(58)-C(59)-C(60)-C(61)	-1.1(11)
C(59)-C(60)-C(61)-C(62)	0.6(11)
C(58)-C(57)-C(62)-C(61)	-3.4(9)
B(2)-C(57)-C(62)-C(61)	177.5(6)
C(60)-C(61)-C(62)-C(57)	1.8(10)
C(45)-B(2)-C(63)-C(68)	82.9(7)
C(51)-B(2)-C(63)-C(68)	-34.6(8)
C(57)-B(2)-C(63)-C(68)	-156.0(5)
C(45)-B(2)-C(63)-C(64)	-96.0(7)
C(51)-B(2)-C(63)-C(64)	146.4(6)
C(57)-B(2)-C(63)-C(64)	25.0(8)
C(68)-C(63)-C(64)-C(65)	0.9(10)
B(2)-C(63)-C(64)-C(65)	180.0(6)
C(63)-C(64)-C(65)-C(66)	-0.8(11)
C(64)-C(65)-C(66)-C(67)	0.0(10)
C(65)-C(66)-C(67)-C(68)	0.5(10)
C(66)-C(67)-C(68)-C(63)	-0.4(10)
C(64)-C(63)-C(68)-C(67)	-0.3(9)
B(2)-C(63)-C(68)-C(67)	-179.4(6)

Symmetry transformations used to generate equivalent atoms:

3 (Cobaltoceniumselenolate)(triphenylphosphane)gold(I) Hexafluoridophosphate (**3**)

A 100 ml Schlenk flask was charged under an atmosphere of argon with 44.5 mg chloro[tris(triphenylphosphine)]gold(I) (1.0 eq., 0.090 mmol), 24 mg (1.0 eq., 0.090 mmol) of freshly prepared cobaltocenium selenolate[2] (assumed yield of 80%) and 10 ml dry acetonitrile. The solution quickly turns red and was stirred for six days at room temperature and under argon atmosphere. In this time a black residue was formed, which was filtered off at ambient conditions and washed with acetonitrile. The solvent was removed from the dark red solution using a rotary evaporator, redissolved in 2 ml acetonitrile and crystallized by diffusion−crystallization with diethylether at 4°C. The red needle−shaped crystals were filtered off and washed with diethylether to yield 61% (48 mg, 0.055 mmol) of pure product.

3.1 Analytical Data

^{1}H NMR (300 MHz, CD_3CN): δ 7.56 (m, 15H, PPh_3), 5.67 (cobaltocenium), 5.64 (pseudo-t,^{3}J = 1.9 Hz 2H, Cp-Se), 5.48 (pseudo-t,^{3}J = 1.9 Hz, 2H, Cp-Se), 5.29 (s, 5H, Cp), 2.15 (H_2O), 2.09 (CH_3CN), 1.94 (CD_3CN) ppm.

^{13}C NMR (75 MHz, CD_3CN): δ 135.46 (PPh_3), 135.28 (PPh_3), 133.59 (PPh_3), 133.57 (PPh_3), 131.01 (PPh_3), 130.86 (PPh_3), 130.68 (PPh_3), 129.93 (PPh_3), 118.68 (CD_3CN), 104.16 (Cp-Se ipso), 88.56 (Cp-Se), 87.65 (Cp), 84.59 (Cp-Se), 1.67 (CD_3CN) ppm.

^{31}P NMR (121 MHz, CD_3CN): δ 39.22, -143.28 ppm.

^{77}Se NMR (57 MHz, CD_3CN): δ -1209.57 (vs. H_2SeO_3), 595.76 (vs. MeSeMe) ppm.

MS (ESI+): *m/z* calc. 726.9773 (M^+), found 726.9762 (M^+)

UV-Vis (CH_3CN): λ_{max} 246 nm (ε = 23211 L mol^{-1} cm^{-1}), 279 nm (ε = 9167 L mol^{-1} cm^{-1}), 452 nm (ε = 3906 L mol^{-1} cm^{-1}).

IR (ATR): 3116 w, 2957 m / 2926 m / 2857 m (ν_{C-H}), 1726 m, 1437 m/ 1409 m (ν_{C-C}), 1272 m, 1121 m / 1101 m (δ_{C-C}), 1071 m (δ_{C-H}), 852 m (CH_{oop}), 826 s (ν_{P-F}), 747 s, 693 s, 556 s (ν_{P-F}), 534 s, 508 s, 497 s (ν_{Co-C}), 454 s (δ_{Co-C}) cm^{-1}.

Melting point: 162.2°C

3.2 Spectra

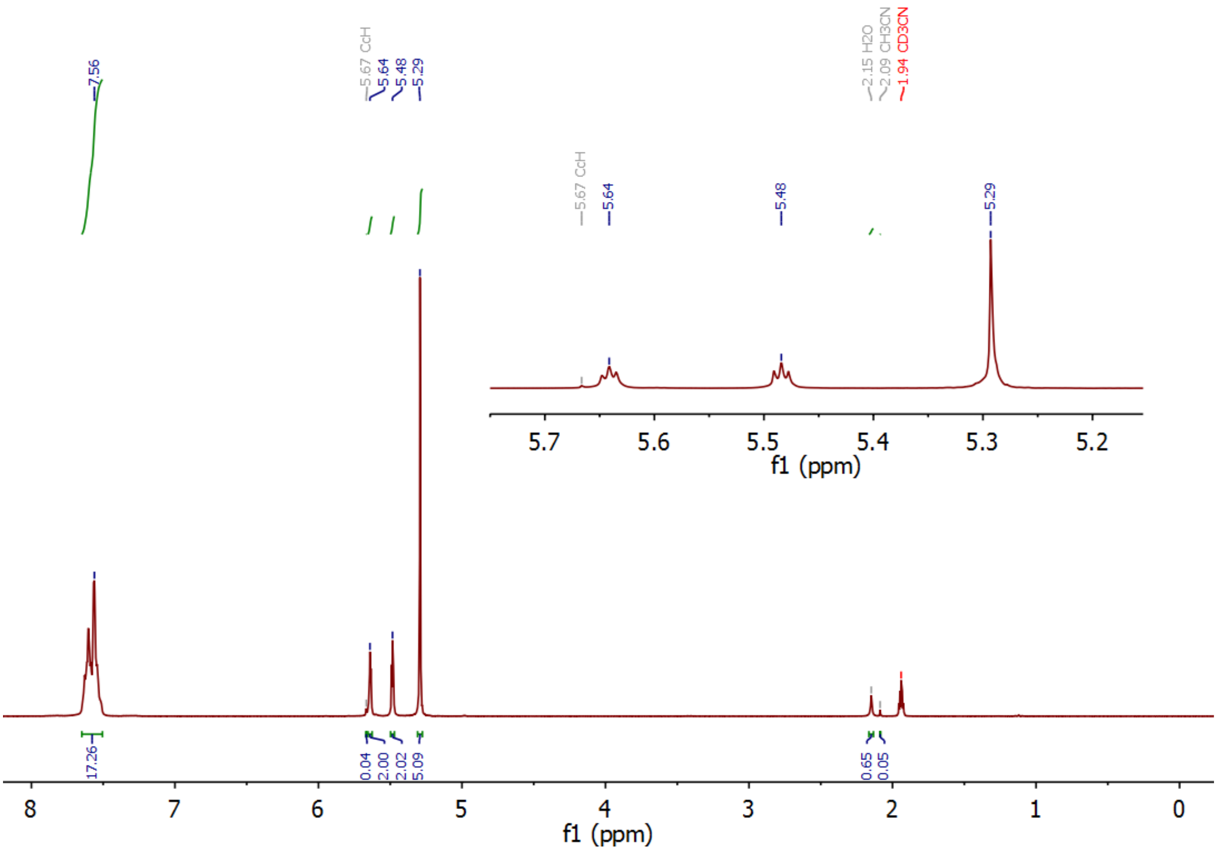

*Figure S8: ^{1}H NMR (300 MHz, CD_3CN) **3**.*

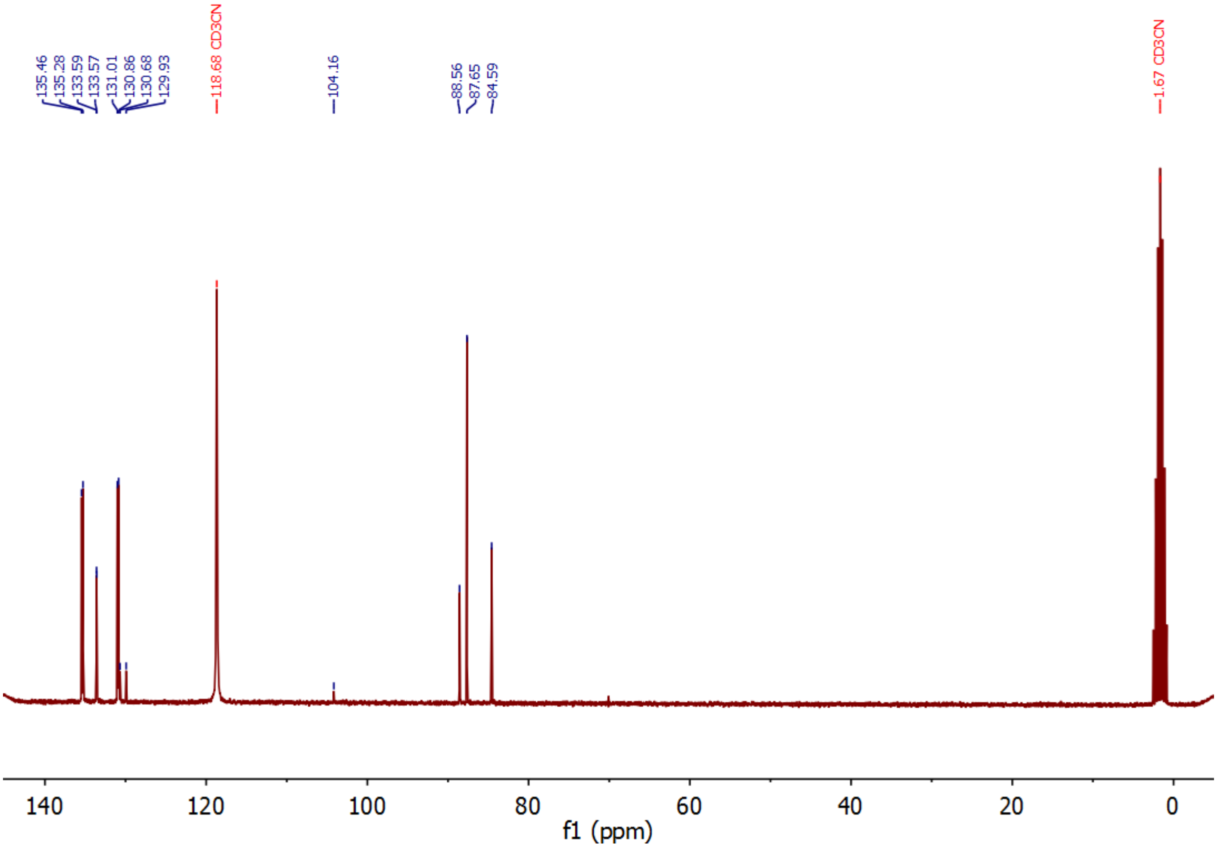

*Figure S9: ^{13}C NMR (75 MHz, CD_3CN) **3**.*

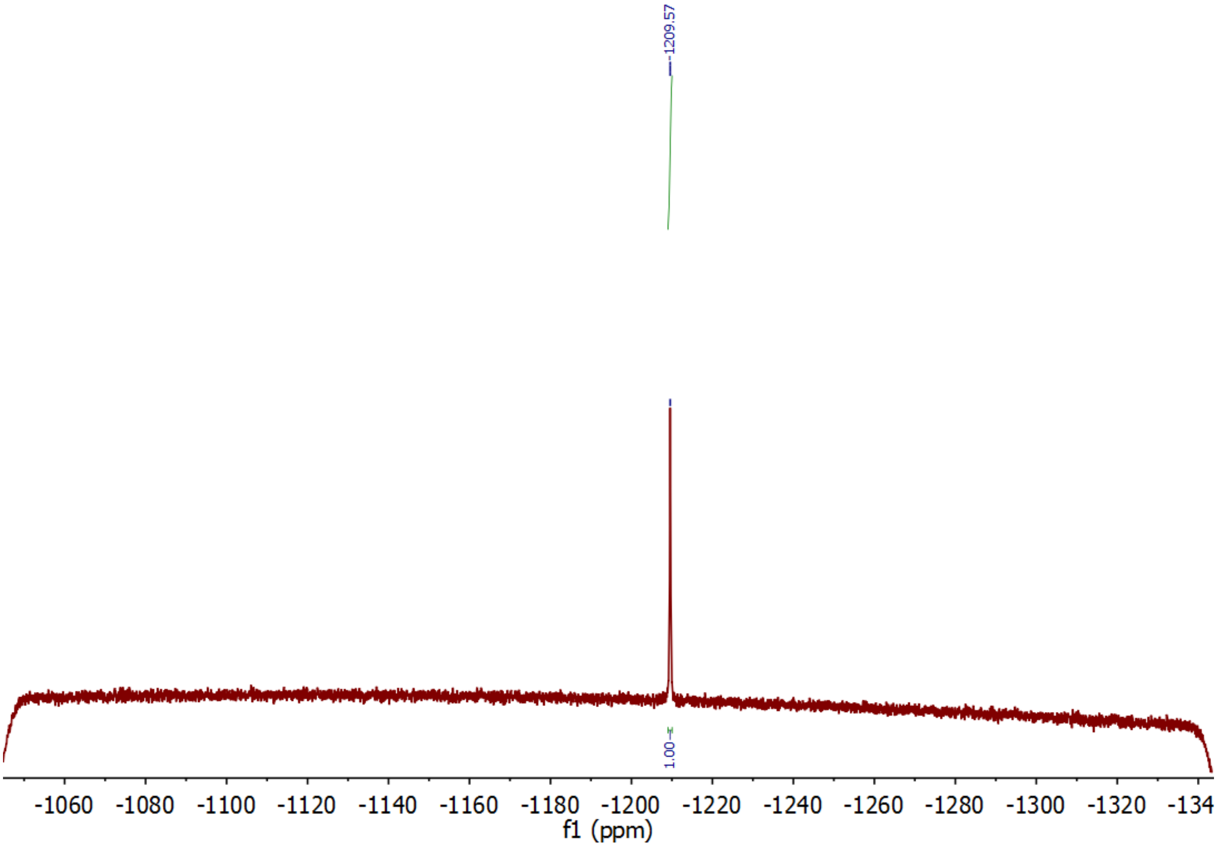

*Figure S10: ^{77}Se NMR (57 MHz, CD_3CN) **3**.*

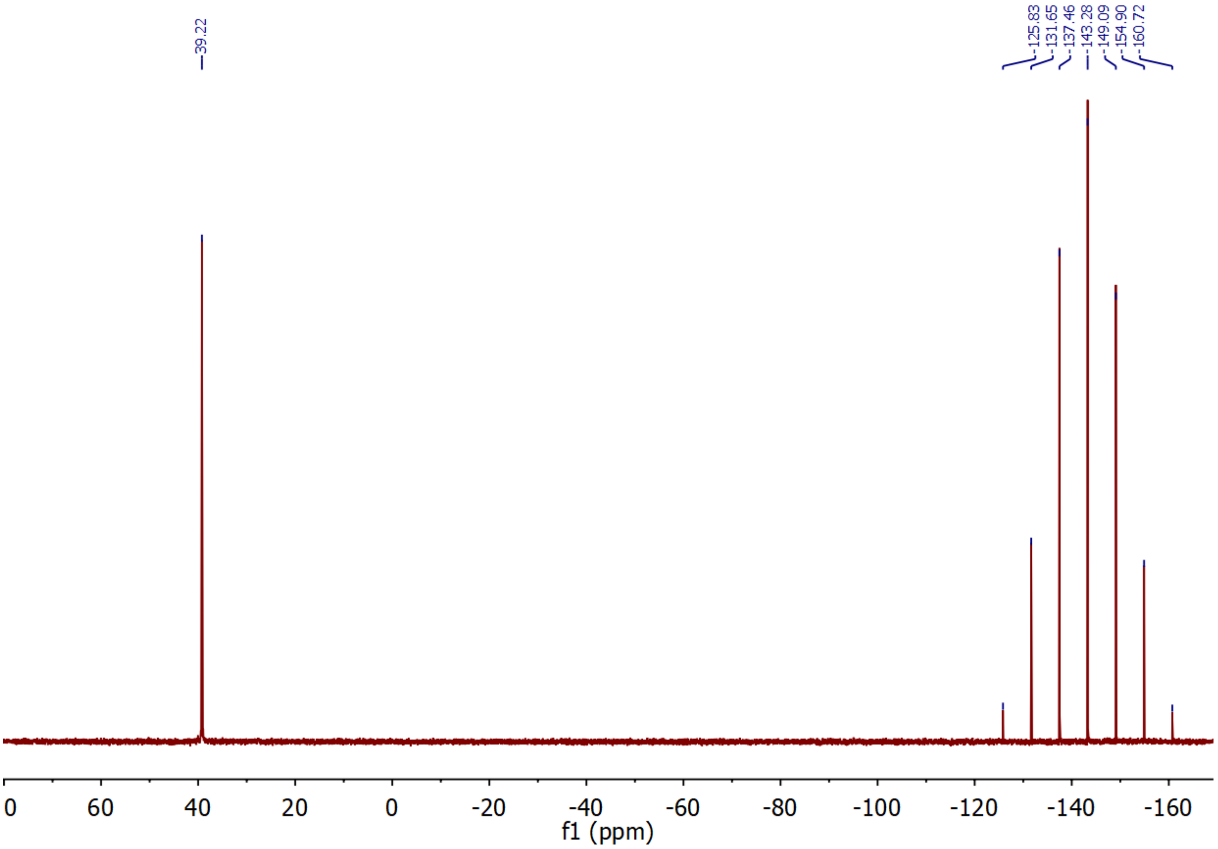

*Figure S11: ^{31}P NMR (121 MHz, CD_3CN) **3**.*

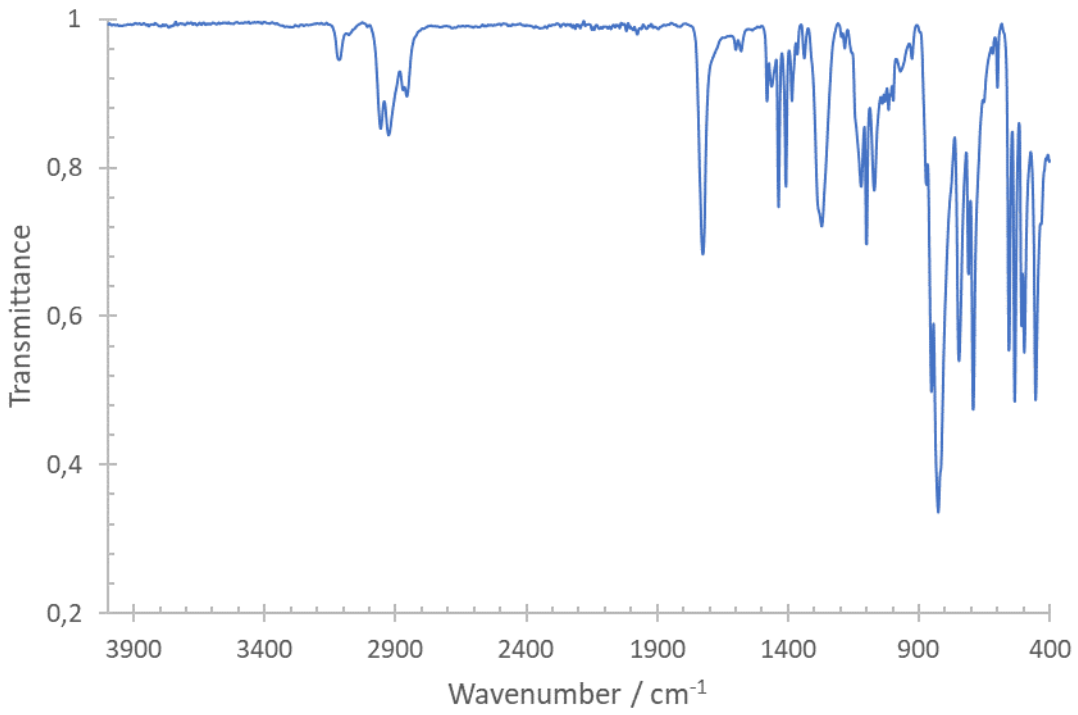

Figure S12: IR (ATR) ***3****.*

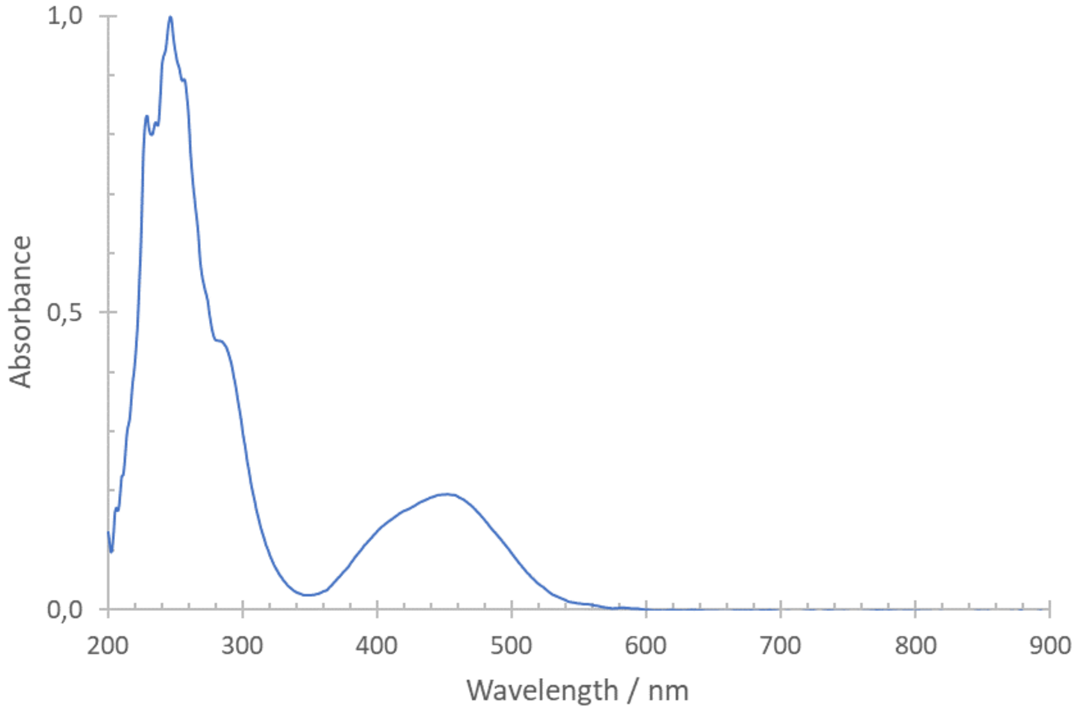

Figure S13: UV-Vis (CH_3CN) ***3****.*

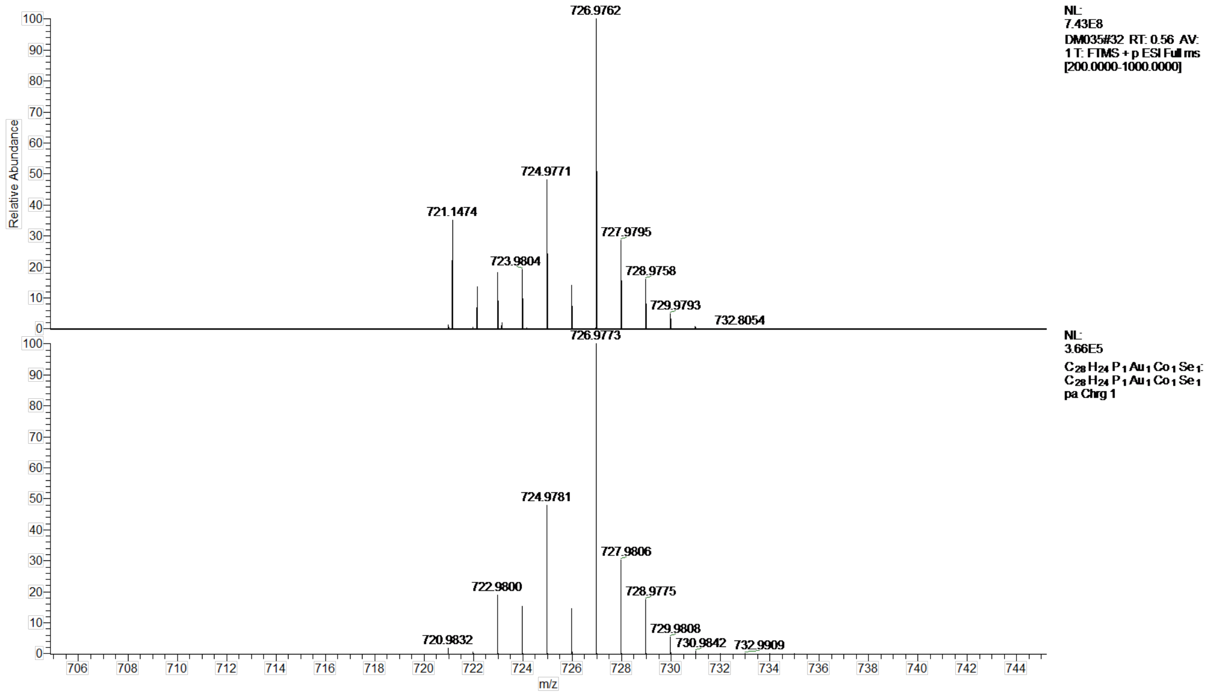

Figure S14: MS (ESI+) ***3****.*

3.3 Crystallographic Data

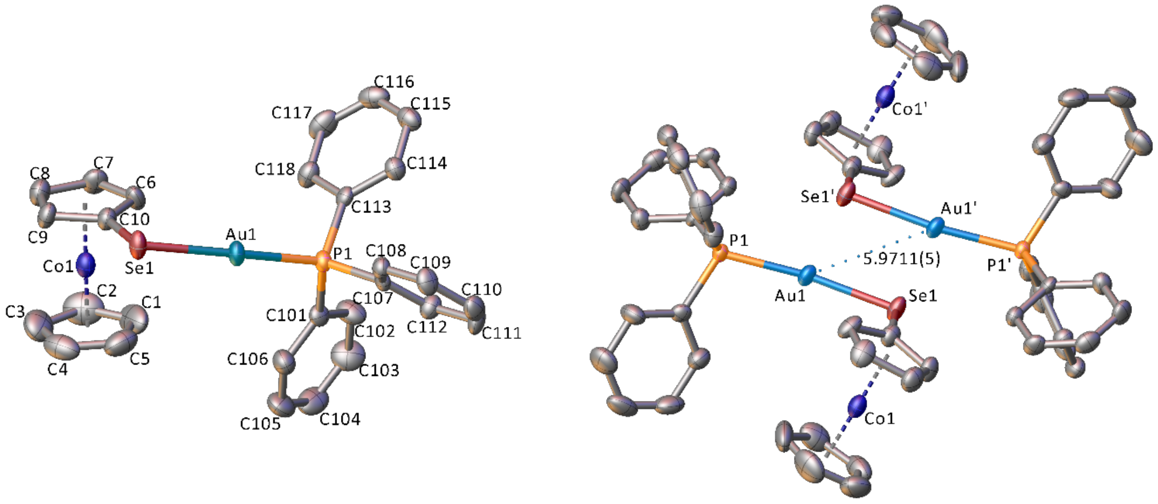

Counterion hexafluoridophosphate omitted for clarity.

Table S7. Crystal data and structure refinement for **3**.		
Empirical formula	C_{28} H_{24} Au Co F_6 P_2 Se	
Formula weight	871.27	
Temperature	193(2) K	
Wavelength	0.71073 Å	
Crystal system	Monoclinic	
Space group	C2/c (no. 15)	
Unit cell dimensions	a = 16.1774(12) Å	α = 90°
	b = 24.4186(18) Å	β = 111.440(2)°
	c = 15.3560(18) Å	γ = 90°
Volume	5646.3(9) $Å^3$	
Z	8	
Density (calculated)	2.050 Mg/m^3	
Absorption coefficient	7.238 mm^{-1}	
F(000)	3328	
Crystal size	0.120 x 0.050 x 0.030 mm_3	
Theta range for data collection	2.194 to 24.997°.	
Index ranges	$-19 \le h \le 18$, $-28 \le k \le 28$, $-16 \le l \le 18$	
Reflections collected	41942	
Independent reflections	4984 [R(int) = 0.0490]	
Completeness to theta = 24.997°	99.9 %	
Absorption correction	Semi-empirical from equivalents	
Max. and min. transmission	0.801 and 0.523	
Refinement method	Full-matrix least-squares on F^2	
Data / restraints / parameters	4984 / 0 / 390	
Goodness-of-fit on F^2	1.096	
Final R indices [I>2sigma(I)]	R1 = 0.0302, wR2 = 0.0607	
R indices (all data)	R1 = 0.0461, wR2 = 0.0669	
Extinction coefficient	n/a	
Largest diff. peak and hole	1.277 and -1.060 e.$Å^{-3}$	

Table S8. Atomic coordinates (x 10^4) and equivalent isotropic displacement parameters (Å^2 x 10^3) for **3**. U(eq) is defined as one third of the trace of the orthogonalized U^{ij} tensor.

	x	y	z	U(eq)
Au(1)	4231(1)	4274(1)	3333(1)	29(1)
Se(1)	5678(1)	4697(1)	3720(1)	34(1)
Co(1)	6719(1)	3785(1)	5640(1)	31(1)
P(1)	2987(1)	3798(1)	3197(1)	23(1)
C(1)	6203(5)	3019(3)	5414(7)	68(2)
C(2)	6976(6)	3020(3)	6192(6)	67(2)
C(3)	7667(5)	3193(3)	5924(6)	62(2)
C(4)	7308(5)	3304(3)	4959(5)	55(2)
C(5)	6400(5)	3200(3)	4644(5)	58(2)
C(6)	5733(4)	4315(2)	5599(4)	35(1)
C(7)	6358(4)	4260(3)	6517(4)	42(2)
C(8)	7200(4)	4429(2)	6506(4)	41(2)
C(9)	7089(4)	4582(2)	5588(4)	34(1)
C(10)	6168(3)	4519(2)	5006(4)	31(1)
C(101)	3276(3)	3079(2)	3435(3)	27(1)
C(102)	2840(4)	2739(2)	3851(4)	42(2)
C(103)	3067(5)	2191(3)	3994(5)	54(2)
C(104)	3725(5)	1981(3)	3730(5)	53(2)
C(105)	4158(4)	2309(3)	3317(5)	53(2)
C(106)	3938(4)	2860(2)	3167(4)	40(1)
C(107)	2076(3)	3815(2)	2082(3)	23(1)
C(108)	1985(3)	4262(2)	1500(3)	27(1)
C(109)	1276(3)	4287(2)	651(4)	33(1)
C(110)	666(4)	3867(2)	388(4)	37(1)
C(111)	740(4)	3426(2)	971(4)	38(1)
C(112)	1448(3)	3398(2)	1817(4)	32(1)
C(113)	2516(3)	3996(2)	4059(3)	24(1)
C(114)	1649(3)	4184(2)	3799(4)	30(1)
C(115)	1323(4)	4342(2)	4481(4)	36(1)
C(116)	1855(4)	4313(2)	5409(4)	42(2)
C(117)	2724(5)	4127(3)	5683(4)	45(2)
C(118)	3058(4)	3971(2)	5004(4)	34(1)
P(2)	10000	4351(1)	7500	34(1)
P(3)	5000	3032(1)	7500	46(1)
F(1)	10633(3)	4334(2)	6924(3)	84(2)
F(7)	3981(3)	3042(3)	7323(4)	121(2)
F(8)	4803(16)	2458(5)	7059(13)	158(10)
F(9)	5172(11)	3604(5)	7920(12)	128(8)
F(10)	4838(8)	3267(9)	6492(9)	109(6)
F(11)	5144(12)	2786(9)	8469(12)	136(7)
F(2)	9165(5)	4384(4)	6555(5)	59(2)
F(3)	10000	5004(4)	7500	104(5)
F(4)	10000	3733(3)	7500	94(3)

F(5)	10580(30)	4710(20)	8130(30)	170(20)
F(6)	9413(18)	3819(16)	6810(20)	115(12)

Table S9. Bond lengths [Å] and angles [°] for **3**.

Au(1)-P(1)	2.2673(13)
Au(1)-Se(1)	2.4249(6)
Se(1)-C(10)	1.891(5)
Co(1)-C(5)	2.019(6)
Co(1)-C(7)	2.019(6)
Co(1)-C(8)	2.021(6)
Co(1)-C(1)	2.027(7)
Co(1)-C(4)	2.027(7)
Co(1)-C(2)	2.030(7)
Co(1)-C(3)	2.035(7)
Co(1)-C(6)	2.037(5)
Co(1)-C(9)	2.046(6)
Co(1)-C(10)	2.079(6)
P(1)-C(107)	1.806(5)
P(1)-C(113)	1.817(5)
P(1)-C(101)	1.819(5)
C(1)-C(2)	1.378(11)
C(1)-C(5)	1.404(10)
C(1)-H(1)	0.9500
C(2)-C(3)	1.391(10)
C(2)-H(2)	0.9500
C(3)-C(4)	1.406(10)
C(3)-H(3)	0.9500
C(4)-C(5)	1.393(10)
C(4)-H(4)	0.9500
C(5)-H(5)	0.9500
C(6)-C(7)	1.410(8)
C(6)-C(10)	1.429(8)
C(6)-H(6)	0.9500
C(7)-C(8)	1.429(8)
C(7)-H(7)	0.9500
C(8)-C(9)	1.404(8)
C(8)-H(8)	0.9500
C(9)-C(10)	1.438(7)
C(9)-H(9)	0.9500
C(101)-C(106)	1.386(8)
C(101)-C(102)	1.388(8)
C(102)-C(103)	1.382(8)
C(102)-H(102)	0.9500
C(103)-C(104)	1.371(10)
C(103)-H(103)	0.9500
C(104)-C(105)	1.364(10)
C(104)-H(104)	0.9500
C(105)-C(106)	1.388(9)
C(105)-H(105)	0.9500
C(106)-H(106)	0.9500
C(107)-C(108)	1.384(7)
C(107)-C(112)	1.391(7)
C(108)-C(109)	1.387(7)
C(108)-H(108)	0.9500
C(109)-C(110)	1.378(8)
C(109)-H(109)	0.9500
C(110)-C(111)	1.378(8)
C(110)-H(110)	0.9500
C(111)-C(112)	1.384(7)
C(111)-H(111)	0.9500
C(112)-H(112)	0.9500
C(113)-C(114)	1.389(7)
C(113)-C(118)	1.396(7)
C(114)-C(115)	1.389(8)
C(114)-H(114)	0.9500
C(115)-C(116)	1.372(8)
C(115)-H(115)	0.9500
C(116)-C(117)	1.388(9)
C(116)-H(116)	0.9500
C(117)-C(118)	1.392(8)
C(117)-H(117)	0.9500
C(118)-H(118)	0.9500
P(2)-F(5)	1.38(2)
P(2)-F(5)#1	1.38(2)
P(2)-F(4)	1.510(8)
P(2)-F(1)	1.579(4)
P(2)-F(1)#1	1.579(4)
P(2)-F(2)	1.583(6)
P(2)-F(2)#1	1.583(6)
P(2)-F(3)	1.594(11)
P(2)-F(6)#1	1.72(2)
P(2)-F(6)	1.72(2)
P(3)-F(9)#2	1.521(11)
P(3)-F(9)	1.521(11)
P(3)-F(8)#2	1.538(13)
P(3)-F(8)	1.538(13)
P(3)-F(11)#2	1.540(13)
P(3)-F(11)	1.540(13)

P(3)-F(7)#2	1.569(4)
P(3)-F(7)	1.569(4)
P(3)-F(10)#2	1.580(11)
P(3)-F(10)	1.580(11)
F(8)-F(11)#2	1.16(2)
F(8)-F(8)#2	1.27(4)
F(9)-F(9)#2	1.20(3)
F(9)-F(10)#2	1.23(2)
F(10)-F(11)#2	1.17(2)
F(10)-F(9)#2	1.23(2)
F(11)-F(8)#2	1.16(2)
F(11)-F(10)#2	1.17(2)
P(1)-Au(1)-Se(1)	170.37(4)
C(10)-Se(1)-Au(1)	99.01(16)
C(5)-Co(1)-C(7)	148.9(3)
C(5)-Co(1)-C(8)	168.4(3)
C(7)-Co(1)-C(8)	41.4(2)
C(5)-Co(1)-C(1)	40.6(3)
C(7)-Co(1)-C(1)	116.3(3)
C(8)-Co(1)-C(1)	150.0(3)
C(5)-Co(1)-C(4)	40.3(3)
C(7)-Co(1)-C(4)	169.2(3)
C(8)-Co(1)-C(4)	130.5(3)
C(1)-Co(1)-C(4)	67.6(3)
C(5)-Co(1)-C(2)	67.7(3)
C(7)-Co(1)-C(2)	108.4(3)
C(8)-Co(1)-C(2)	118.1(3)
C(1)-Co(1)-C(2)	39.7(3)
C(4)-Co(1)-C(2)	67.6(3)
C(5)-Co(1)-C(3)	67.9(3)
C(7)-Co(1)-C(3)	130.1(3)
C(8)-Co(1)-C(3)	109.4(3)
C(1)-Co(1)-C(3)	67.2(3)
C(4)-Co(1)-C(3)	40.5(3)
C(2)-Co(1)-C(3)	40.0(3)
C(5)-Co(1)-C(6)	116.4(3)
C(7)-Co(1)-C(6)	40.7(2)
C(8)-Co(1)-C(6)	68.7(2)
C(1)-Co(1)-C(6)	108.1(3)
C(4)-Co(1)-C(6)	149.6(3)
C(2)-Co(1)-C(6)	129.4(3)
C(3)-Co(1)-C(6)	168.0(3)
C(5)-Co(1)-C(9)	129.9(3)
C(7)-Co(1)-C(9)	68.8(2)
C(8)-Co(1)-C(9)	40.4(2)
C(1)-Co(1)-C(9)	168.4(3)
C(4)-Co(1)-C(9)	109.4(2)
C(2)-Co(1)-C(9)	150.9(3)
C(3)-Co(1)-C(9)	118.4(3)
C(6)-Co(1)-C(9)	68.4(2)
C(5)-Co(1)-C(10)	108.0(3)
C(7)-Co(1)-C(10)	68.8(2)
C(8)-Co(1)-C(10)	68.5(2)
C(1)-Co(1)-C(10)	129.5(3)
C(4)-Co(1)-C(10)	117.4(3)
C(2)-Co(1)-C(10)	167.4(3)
C(3)-Co(1)-C(10)	150.8(3)
C(6)-Co(1)-C(10)	40.6(2)
C(9)-Co(1)-C(10)	40.8(2)
C(107)-P(1)-C(113)	105.7(2)
C(107)-P(1)-C(101)	105.9(2)
C(113)-P(1)-C(101)	104.9(2)
C(107)-P(1)-Au(1)	117.70(16)
C(113)-P(1)-Au(1)	113.00(16)
C(101)-P(1)-Au(1)	108.77(17)
C(2)-C(1)-C(5)	108.4(7)
C(2)-C(1)-Co(1)	70.3(4)
C(5)-C(1)-Co(1)	69.4(4)
C(2)-C(1)-H(1)	125.8
C(5)-C(1)-H(1)	125.8
Co(1)-C(1)-H(1)	126.1
C(1)-C(2)-C(3)	108.5(7)
C(1)-C(2)-Co(1)	70.0(4)
C(3)-C(2)-Co(1)	70.1(4)
C(1)-C(2)-H(2)	125.7
C(3)-C(2)-H(2)	125.7
Co(1)-C(2)-H(2)	125.7
C(2)-C(3)-C(4)	107.7(7)
C(2)-C(3)-Co(1)	69.8(4)
C(4)-C(3)-Co(1)	69.5(4)
C(2)-C(3)-H(3)	126.2
C(4)-C(3)-H(3)	126.2
Co(1)-C(3)-H(3)	126.1
C(5)-C(4)-C(3)	107.9(6)
C(5)-C(4)-Co(1)	69.5(4)
C(3)-C(4)-Co(1)	70.0(4)
C(5)-C(4)-H(4)	126.0
C(3)-C(4)-H(4)	126.0
Co(1)-C(4)-H(4)	126.0
C(4)-C(5)-C(1)	107.5(7)
C(4)-C(5)-Co(1)	70.2(4)
C(1)-C(5)-Co(1)	70.0(4)
C(4)-C(5)-H(5)	126.3
C(1)-C(5)-H(5)	126.3

Co(1)-C(5)-H(5)	125.1
C(7)-C(6)-C(10)	109.2(5)
C(7)-C(6)-Co(1)	69.0(3)
C(10)-C(6)-Co(1)	71.3(3)
C(7)-C(6)-H(6)	125.4
C(10)-C(6)-H(6)	125.4
Co(1)-C(6)-H(6)	125.9
C(6)-C(7)-C(8)	107.4(5)
C(6)-C(7)-Co(1)	70.3(3)
C(8)-C(7)-Co(1)	69.4(3)
C(6)-C(7)-H(7)	126.3
C(8)-C(7)-H(7)	126.3
Co(1)-C(7)-H(7)	125.6
C(9)-C(8)-C(7)	108.4(5)
C(9)-C(8)-Co(1)	70.8(3)
C(7)-C(8)-Co(1)	69.2(3)
C(9)-C(8)-H(8)	125.8
C(7)-C(8)-H(8)	125.8
Co(1)-C(8)-H(8)	125.8
C(8)-C(9)-C(10)	108.6(5)
C(8)-C(9)-Co(1)	68.8(3)
C(10)-C(9)-Co(1)	70.9(3)
C(8)-C(9)-H(9)	125.7
C(10)-C(9)-H(9)	125.7
Co(1)-C(9)-H(9)	126.2
C(6)-C(10)-C(9)	106.4(5)
C(6)-C(10)-Se(1)	129.0(4)
C(9)-C(10)-Se(1)	124.7(4)
C(6)-C(10)-Co(1)	68.1(3)
C(9)-C(10)-Co(1)	68.4(3)
Se(1)-C(10)-Co(1)	129.2(3)
C(106)-C(101)-C(102)	118.9(5)
C(106)-C(101)-P(1)	118.9(4)
C(102)-C(101)-P(1)	122.2(4)
C(103)-C(102)-C(101)	120.1(6)
C(103)-C(102)-H(102)	120.0
C(101)-C(102)-H(102)	120.0
C(104)-C(103)-C(102)	120.4(6)
C(104)-C(103)-H(103)	119.8
C(102)-C(103)-H(103)	119.8
C(105)-C(104)-C(103)	120.2(6)
C(105)-C(104)-H(104)	119.9
C(103)-C(104)-H(104)	119.9
C(104)-C(105)-C(106)	120.1(6)
C(104)-C(105)-H(105)	120.0
C(106)-C(105)-H(105)	120.0
C(101)-C(106)-C(105)	120.3(6)
C(101)-C(106)-H(106)	119.8
C(105)-C(106)-H(106)	119.8
C(108)-C(107)-C(112)	119.7(5)
C(108)-C(107)-P(1)	119.2(4)
C(112)-C(107)-P(1)	121.1(4)
C(107)-C(108)-C(109)	119.8(5)
C(107)-C(108)-H(108)	120.1
C(109)-C(108)-H(108)	120.1
C(110)-C(109)-C(108)	120.0(5)
C(110)-C(109)-H(109)	120.0
C(108)-C(109)-H(109)	120.0
C(109)-C(110)-C(111)	120.6(5)
C(109)-C(110)-H(110)	119.7
C(111)-C(110)-H(110)	119.7
C(110)-C(111)-C(112)	119.6(5)
C(110)-C(111)-H(111)	120.2
C(112)-C(111)-H(111)	120.2
C(111)-C(112)-C(107)	120.2(5)
C(111)-C(112)-H(112)	119.9
C(107)-C(112)-H(112)	119.9
C(114)-C(113)-C(118)	119.9(5)
C(114)-C(113)-P(1)	121.8(4)
C(118)-C(113)-P(1)	118.3(4)
C(115)-C(114)-C(113)	119.8(5)
C(115)-C(114)-H(114)	120.1
C(113)-C(114)-H(114)	120.1
C(116)-C(115)-C(114)	120.2(5)
C(116)-C(115)-H(115)	119.9
C(114)-C(115)-H(115)	119.9
C(115)-C(116)-C(117)	120.9(6)
C(115)-C(116)-H(116)	119.6
C(117)-C(116)-H(116)	119.6
C(116)-C(117)-C(118)	119.4(6)
C(116)-C(117)-H(117)	120.3
C(118)-C(117)-H(117)	120.3
C(117)-C(118)-C(113)	119.9(5)
C(117)-C(118)-H(118)	120.1
C(113)-C(118)-H(118)	120.1
F(5)-P(2)-F(5)#1	102(5)
F(5)-P(2)-F(1)	89.9(15)
F(5)#1-P(2)-F(1)	92.1(15)
F(4)-P(2)-F(1)	88.5(2)
F(5)-P(2)-F(1)#1	92.1(15)
F(5)#1-P(2)-F(1)#1	89.9(15)
F(4)-P(2)-F(1)#1	88.5(2)
F(1)-P(2)-F(1)#1	176.9(4)
F(4)-P(2)-F(2)	92.9(3)

F(1)-P(2)-F(2)	89.9(3)
F(1)#1-P(2)-F(2)	90.2(3)
F(4)-P(2)-F(2)#1	92.8(3)
F(1)-P(2)-F(2)#1	90.2(3)
F(1)#1-P(2)-F(2)#1	89.9(3)
F(2)-P(2)-F(2)#1	174.3(7)
F(4)-P(2)-F(3)	180.0
F(1)-P(2)-F(3)	91.5(2)
F(1)#1-P(2)-F(3)	91.5(2)
F(2)-P(2)-F(3)	87.1(3)
F(2)#1-P(2)-F(3)	87.2(3)
F(5)-P(2)-F(6)#1	88(3)
F(5)#1-P(2)-F(6)#1	170(3)
F(1)-P(2)-F(6)#1	89.8(8)
F(1)#1-P(2)-F(6)#1	87.9(8)
F(5)-P(2)-F(6)	170(3)
F(5)#1-P(2)-F(6)	88(3)
F(1)-P(2)-F(6)	87.9(8)
F(1)#1-P(2)-F(6)	89.8(8)
F(6)#1-P(2)-F(6)	82(3)
F(9)#2-P(3)-F(9)	46.6(13)
F(9)#2-P(3)-F(8)#2	178.6(12)
F(9)-P(3)-F(8)#2	132.3(9)
F(9)#2-P(3)-F(8)	132.3(9)
F(9)-P(3)-F(8)	178.6(11)
F(8)#2-P(3)-F(8)	48.7(15)
F(9)#2-P(3)-F(11)#2	90.5(10)
F(9)-P(3)-F(11)#2	135.0(13)
F(8)#2-P(3)-F(11)#2	90.2(14)
F(8)-P(3)-F(11)#2	44.4(8)
F(9)#2-P(3)-F(11)	135.0(13)
F(9)-P(3)-F(11)	90.5(10)
F(8)#2-P(3)-F(11)	44.4(8)
F(8)-P(3)-F(11)	90.2(14)
F(11)#2-P(3)-F(11)	134.1(18)
F(9)#2-P(3)-F(7)#2	94.4(7)
F(9)-P(3)-F(7)#2	84.0(7)
F(8)#2-P(3)-F(7)#2	84.4(9)
F(8)-P(3)-F(7)#2	97.2(9)
F(11)#2-P(3)-F(7)#2	87.2(7)
F(11)-P(3)-F(7)#2	93.4(7)
F(9)#2-P(3)-F(7)	84.0(7)
F(9)-P(3)-F(7)	94.4(7)
F(8)#2-P(3)-F(7)	97.2(9)
F(8)-P(3)-F(7)	84.4(9)
F(11)#2-P(3)-F(7)	93.4(7)
F(11)-P(3)-F(7)	87.2(7)
F(7)#2-P(3)-F(7)	178.3(6)
F(9)#2-P(3)-F(10)#2	91.3(14)
F(9)-P(3)-F(10)#2	46.6(8)
F(8)#2-P(3)-F(10)#2	88.0(10)
F(8)-P(3)-F(10)#2	134.0(12)
F(11)#2-P(3)-F(10)#2	178.2(16)
F(11)-P(3)-F(10)#2	44.2(8)
F(7)#2-P(3)-F(10)#2	92.1(5)
F(7)-P(3)-F(10)#2	87.2(5)
F(9)#2-P(3)-F(10)	46.6(8)
F(9)-P(3)-F(10)	91.3(14)
F(8)#2-P(3)-F(10)	134.0(12)
F(8)-P(3)-F(10)	88.0(10)
F(11)#2-P(3)-F(10)	44.2(8)
F(11)-P(3)-F(10)	178.2(16)
F(7)#2-P(3)-F(10)	87.2(5)
F(7)-P(3)-F(10)	92.1(5)
F(10)#2-P(3)-F(10)	137.6(18)
F(11)#2-F(8)-F(8)#2	127(2)
F(11)#2-F(8)-P(3)	67.9(10)
F(8)#2-F(8)-P(3)	65.6(7)
F(9)#2-F(9)-F(10)#2	131.5(14)
F(9)#2-F(9)-P(3)	66.7(7)
F(10)#2-F(9)-P(3)	69.2(9)
F(11)#2-F(10)-F(9)#2	129.7(15)
F(11)#2-F(10)-P(3)	66.1(9)
F(9)#2-F(10)-P(3)	64.2(8)
F(8)#2-F(11)-F(10)#2	135.8(16)
F(8)#2-F(11)-P(3)	67.7(10)
F(10)#2-F(11)-P(3)	69.7(10)

Symmetry transformations used to generate equivalent atoms:
#1 -x+2,y,-z+3/2 #2 -x+1,y,-z+3/2

Table S10. Anisotropic displacement parameters (Å^2 x 10^3) for **3**. The anisotropic displacement factor exponent takes the form: $-2\pi^2$ [h^2 a^{*2} U^{11} + ... + 2 h k a* b* U^{12}]

	U^{11}	U^{22}	U^{33}	U^{23}	U^{13}	U^{12}
Au(1)	18(1)	36(1)	32(1)	1(1)	6(1)	-4(1)
Se(1)	20(1)	49(1)	33(1)	3(1)	8(1)	-8(1)

Co(1)	24(1)	39(1)	30(1)	1(1)	10(1)	-5(1)
P(1)	18(1)	24(1)	27(1)	2(1)	7(1)	-2(1)
C(1)	58(5)	44(4)	117(7)	-19(4)	52(5)	-24(4)
C(2)	103(7)	48(4)	66(5)	18(4)	49(5)	-1(4)
C(3)	47(4)	49(4)	77(5)	5(4)	9(4)	16(3)
C(4)	76(5)	33(3)	79(5)	5(3)	55(4)	2(3)
C(5)	71(5)	45(4)	47(4)	-12(3)	10(4)	-1(4)
C(6)	22(3)	54(4)	32(3)	-7(3)	12(2)	1(3)
C(7)	43(4)	57(4)	29(3)	-10(3)	17(3)	-6(3)
C(8)	29(3)	48(4)	41(4)	-7(3)	6(3)	-6(3)
C(9)	25(3)	41(3)	32(3)	-5(3)	6(2)	-11(2)
C(10)	24(3)	36(3)	32(3)	-6(2)	8(2)	-5(2)
C(101)	23(3)	29(3)	24(3)	3(2)	3(2)	4(2)
C(102)	50(4)	28(3)	55(4)	9(3)	28(3)	9(3)
C(103)	70(5)	39(4)	54(4)	17(3)	25(4)	4(3)
C(104)	57(4)	33(3)	53(4)	3(3)	0(3)	15(3)
C(105)	40(4)	49(4)	67(5)	-9(4)	15(3)	16(3)
C(106)	30(3)	40(3)	51(4)	-4(3)	16(3)	0(3)
C(107)	15(2)	28(3)	27(3)	0(2)	9(2)	-1(2)
C(108)	21(3)	28(3)	30(3)	-1(2)	8(2)	-3(2)
C(109)	27(3)	39(3)	31(3)	12(3)	6(2)	5(3)
C(110)	26(3)	52(4)	27(3)	-2(3)	4(2)	4(3)
C(111)	23(3)	42(3)	40(3)	-2(3)	1(3)	-9(2)
C(112)	31(3)	29(3)	31(3)	2(2)	7(2)	-4(2)
C(113)	22(3)	21(3)	27(3)	-2(2)	7(2)	-7(2)
C(114)	26(3)	32(3)	31(3)	3(2)	10(2)	1(2)
C(115)	35(3)	32(3)	47(4)	2(3)	24(3)	0(3)
C(116)	62(4)	33(3)	45(4)	-4(3)	35(3)	-7(3)
C(117)	60(4)	48(4)	24(3)	1(3)	13(3)	-15(3)
C(118)	32(3)	41(3)	28(3)	-1(2)	9(2)	-10(3)
P(2)	23(1)	52(1)	26(1)	0	6(1)	0
P(3)	38(1)	51(2)	62(2)	0	33(1)	0
F(1)	38(2)	173(5)	47(2)	-16(3)	22(2)	-14(3)
F(7)	41(3)	214(7)	117(4)	-52(4)	42(3)	-39(3)
F(8)	220(20)	86(9)	230(30)	-74(11)	150(30)	-20(13)
F(9)	93(13)	77(7)	200(30)	-62(9)	34(15)	-22(8)
F(10)	59(7)	197(18)	64(8)	62(11)	16(6)	17(11)
F(11)	176(15)	172(17)	114(13)	66(13)	117(12)	50(15)
F(2)	30(3)	106(7)	32(3)	-9(4)	0(2)	-11(4)
F(3)	110(10)	44(5)	103(10)	0	-25(7)	0
F(4)	100(8)	37(5)	133(10)	0	29(8)	0
F(5)	160(50)	210(50)	180(50)	-180(40)	110(40)	-160(40)
F(6)	76(18)	180(30)	100(20)	-100(20)	43(15)	-100(20)

Table S11. Hydrogen coordinates (x 10^4) and isotropic displacement parameters (Å^2 x 10^3) for **3**.

	x	y	z	U(eq)
H(1)	5633	2914	5401	81
H(2)	7028	2919	6807	81
H(3)	8272	3230	6321	74
H(4)	7629	3429	4588	66
H(5)	5989	3243	4021	69
H(6)	5119	4229	5404	42
H(7)	6242	4135	7047	50
H(8)	7743	4435	7030	50
H(9)	7545	4707	5386	41
H(102)	2385	2883	4039	50
H(103)	2764	1960	4276	64
H(104)	3881	1605	3835	64
H(105)	4611	2161	3131	64
H(106)	4242	3087	2881	49
H(108)	2406	4551	1682	32
H(109)	1212	4594	251	40
H(110)	190	3882	-201	44
H(111)	307	3143	794	46
H(112)	1504	3092	2218	38
H(114)	1279	4204	3156	36
H(115)	730	4471	4304	43
H(116)	1626	4420	5871	51
H(117)	3088	4107	6328	54
H(118)	3654	3847	5183	41

Table S12. Torsion angles [°] for **3**.

C(5)-C(1)-C(2)-C(3)	-0.7(9)
Co(1)-C(1)-C(2)-C(3)	-59.8(5)
C(5)-C(1)-C(2)-Co(1)	59.1(5)
C(1)-C(2)-C(3)-C(4)	0.3(8)
Co(1)-C(2)-C(3)-C(4)	-59.4(5)
C(1)-C(2)-C(3)-Co(1)	59.7(5)
C(2)-C(3)-C(4)-C(5)	0.2(8)
Co(1)-C(3)-C(4)-C(5)	-59.4(5)
C(2)-C(3)-C(4)-Co(1)	59.6(5)
C(3)-C(4)-C(5)-C(1)	-0.6(8)
Co(1)-C(4)-C(5)-C(1)	-60.4(5)
C(3)-C(4)-C(5)-Co(1)	59.8(5)
C(2)-C(1)-C(5)-C(4)	0.8(8)
Co(1)-C(1)-C(5)-C(4)	60.5(5)
C(2)-C(1)-C(5)-Co(1)	-59.7(5)
C(10)-C(6)-C(7)-C(8)	-0.4(7)
Co(1)-C(6)-C(7)-C(8)	59.7(4)
C(10)-C(6)-C(7)-Co(1)	-60.1(4)
C(6)-C(7)-C(8)-C(9)	-0.2(7)
Co(1)-C(7)-C(8)-C(9)	60.2(4)
C(6)-C(7)-C(8)-Co(1)	-60.3(4)
C(7)-C(8)-C(9)-C(10)	0.7(7)
Co(1)-C(8)-C(9)-C(10)	59.9(4)
C(7)-C(8)-C(9)-Co(1)	-59.2(4)
C(7)-C(6)-C(10)-C(9)	0.8(6)
Co(1)-C(6)-C(10)-C(9)	-57.9(4)
C(7)-C(6)-C(10)-Se(1)	-177.9(4)
Co(1)-C(6)-C(10)-Se(1)	123.4(5)
C(7)-C(6)-C(10)-Co(1)	58.7(4)
C(8)-C(9)-C(10)-C(6)	-1.0(6)
Co(1)-C(9)-C(10)-C(6)	57.7(4)

C(8)-C(9)-C(10)-Se(1)	177.9(4)
Co(1)-C(9)-C(10)-Se(1)	-123.5(4)
C(8)-C(9)-C(10)-Co(1)	-58.7(4)
Au(1)-Se(1)-C(10)-C(6)	-13.0(5)
Au(1)-Se(1)-C(10)-C(9)	168.5(5)
Au(1)-Se(1)-C(10)-Co(1)	79.4(3)
C(107)-P(1)-C(101)-C(106)	93.2(5)
C(113)-P(1)-C(101)-C(106)	-155.4(4)
Au(1)-P(1)-C(101)-C(106)	-34.2(5)
C(107)-P(1)-C(101)-C(102)	-85.0(5)
C(113)-P(1)-C(101)-C(102)	26.5(5)
Au(1)-P(1)-C(101)-C(102)	147.6(4)
C(106)-C(101)-C(102)-C(103)	0.0(9)
P(1)-C(101)-C(102)-C(103)	178.2(5)
C(101)-C(102)-C(103)-C(104)	0.3(10)
C(102)-C(103)-C(104)-C(105)	-0.6(11)
C(103)-C(104)-C(105)-C(106)	0.5(10)
C(102)-C(101)-C(106)-C(105)	-0.1(9)
P(1)-C(101)-C(106)-C(105)	-178.3(5)
C(104)-C(105)-C(106)-C(101)	-0.1(10)
C(113)-P(1)-C(107)-C(108)	100.5(4)
C(101)-P(1)-C(107)-C(108)	-148.5(4)
Au(1)-P(1)-C(107)-C(108)	-26.7(5)
C(113)-P(1)-C(107)-C(112)	-76.9(5)
C(101)-P(1)-C(107)-C(112)	34.0(5)
Au(1)-P(1)-C(107)-C(112)	155.9(4)
C(112)-C(107)-C(108)-C(109)	-1.0(8)
P(1)-C(107)-C(108)-C(109)	-178.5(4)
C(107)-C(108)-C(109)-C(110)	-0.1(8)
C(108)-C(109)-C(110)-C(111)	1.5(9)
C(109)-C(110)-C(111)-C(112)	-1.8(9)
C(110)-C(111)-C(112)-C(107)	0.7(9)
C(108)-C(107)-C(112)-C(111)	0.7(8)
P(1)-C(107)-C(112)-C(111)	178.1(4)
C(107)-P(1)-C(113)-C(114)	-7.5(5)
C(101)-P(1)-C(113)-C(114)	-119.1(4)
Au(1)-P(1)-C(113)-C(114)	122.6(4)
C(107)-P(1)-C(113)-C(118)	174.3(4)
C(101)-P(1)-C(113)-C(118)	62.7(4)
Au(1)-P(1)-C(113)-C(118)	-55.6(4)
C(118)-C(113)-C(114)-C(115)	-0.4(8)
P(1)-C(113)-C(114)-C(115)	-178.6(4)
C(113)-C(114)-C(115)-C(116)	-0.1(8)
C(114)-C(115)-C(116)-C(117)	0.3(9)
C(115)-C(116)-C(117)-C(118)	0.2(9)
C(116)-C(117)-C(118)-C(113)	-0.7(8)
C(114)-C(113)-C(118)-C(117)	0.8(8)

P(1)-C(113)-C(118)-C(117)	179.1(4)
F(9)#2-P(3)-F(8)-F(11)#2	24(2)
F(8)#2-P(3)-F(8)-F(11)#2	-155(3)
F(11)-P(3)-F(8)-F(11)#2	-172.1(11)
F(7)#2-P(3)-F(8)-F(11)#2	-78.6(15)
F(7)-P(3)-F(8)-F(11)#2	100.7(15)
F(10)#2-P(3)-F(8)-F(11)#2	-178.6(13)
F(10)-P(3)-F(8)-F(11)#2	8.4(15)
F(9)#2-P(3)-F(8)-F(8)#2	178.7(15)
F(11)#2-P(3)-F(8)-F(8)#2	155(3)
F(11)-P(3)-F(8)-F(8)#2	-17(2)
F(7)#2-P(3)-F(8)-F(8)#2	76.0(18)
F(7)-P(3)-F(8)-F(8)#2	-104.7(18)
F(10)#2-P(3)-F(8)-F(8)#2	-24(3)
F(10)-P(3)-F(8)-F(8)#2	163.0(19)
F(8)#2-P(3)-F(9)-F(9)#2	178.7(15)
F(11)#2-P(3)-F(9)-F(9)#2	22(2)
F(11)-P(3)-F(9)-F(9)#2	-164.7(18)
F(7)#2-P(3)-F(9)-F(9)#2	101.9(17)
F(7)-P(3)-F(9)-F(9)#2	-77.4(17)
F(10)#2-P(3)-F(9)-F(9)#2	-159(2)
F(10)-P(3)-F(9)-F(9)#2	14.8(17)
F(9)#2-P(3)-F(9)-F(10)#2	159(2)
F(8)#2-P(3)-F(9)-F(10)#2	-21.9(18)
F(11)#2-P(3)-F(9)-F(10)#2	-178.7(13)
F(11)-P(3)-F(9)-F(10)#2	-5.3(12)
F(7)#2-P(3)-F(9)-F(10)#2	-98.7(10)
F(7)-P(3)-F(9)-F(10)#2	81.9(10)
F(10)-P(3)-F(9)-F(10)#2	174.2(7)
F(9)#2-P(3)-F(10)-F(11)#2	-172.4(17)
F(9)-P(3)-F(10)-F(11)#2	172.8(13)
F(8)#2-P(3)-F(10)-F(11)#2	9.4(18)
F(8)-P(3)-F(10)-F(11)#2	-8.4(15)
F(7)#2-P(3)-F(10)-F(11)#2	88.9(11)
F(7)-P(3)-F(10)-F(11)#2	-92.7(11)
F(10)#2-P(3)-F(10)-F(11)#2	179.0(12)
F(9)-P(3)-F(10)-F(9)#2	-14.8(17)
F(8)#2-P(3)-F(10)-F(9)#2	-178.2(16)
F(8)-P(3)-F(10)-F(9)#2	164.0(13)
F(11)#2-P(3)-F(10)-F(9)#2	172.4(17)
F(7)#2-P(3)-F(10)-F(9)#2	-98.8(11)
F(7)-P(3)-F(10)-F(9)#2	79.6(11)
F(10)#2-P(3)-F(10)-F(9)#2	-8.6(10)
F(9)#2-P(3)-F(11)-F(8)#2	-178.2(16)
F(9)-P(3)-F(11)-F(8)#2	-162.4(16)
F(8)-P(3)-F(11)-F(8)#2	19(2)
F(11)#2-P(3)-F(11)-F(8)#2	11.1(14)

F(7)#2-P(3)-F(11)-F(8)#2	-78.4(16)
F(7)-P(3)-F(11)-F(8)#2	103.2(16)
F(10)#2-P(3)-F(11)-F(8)#2	-168(2)
F(9)#2-P(3)-F(11)-F(10)#2	-10.2(19)
F(9)-P(3)-F(11)-F(10)#2	5.5(12)
F(8)#2-P(3)-F(11)-F(10)#2	168(2)
F(8)-P(3)-F(11)-F(10)#2	-173.2(13)
F(11)#2-P(3)-F(11)-F(10)#2	179.0(12)
F(7)#2-P(3)-F(11)-F(10)#2	89.5(10)
F(7)-P(3)-F(11)-F(10)#2	-88.9(10)

Symmetry transformations used to generate equivalent atoms:
#1 -x + 2, y, -z + 3/2 #2 -x + 1, y, -z + 3/2

4 Di(cobaltoceniumselenolate)gold(I) Hexafluoridophosphate (**4**)

A 100 ml Schlenk flask was charged with 40 mg sodiumselenide (2.4 eq., 0.320 mmol), 122 mg iodocobaltocenium hexafluoridophosphate[1] (2.0 eq., 0.267 mmol) and 40 ml dry THF. This mixture was stirred at room temperature for 24 hours under an argon atmosphere, then 66 mg chloro[tris(triphenylphosphine)]gold(I) (1.0 eq., 0.133 mmol) was added and the solution was stirred for 40 hours under ambient conditions. The black selenium residue was filtered off and washed with acetonitrile. The dark red solution was concentrated to 5 ml and the product was precipitated with 500 ml diethylether at -20°C. The dark red precipitant was filtered off and washed with diethylether to yield 77% (90 mg, 0.102 mmol) of crude product.

To obtain the pure product, 25.7 mg was dissolved in 60 ml methanol and 20 ml water. This mixture was extracted with 50 ml n-pentane. The aqueous phase was concentrated to 40 ml using a rotary evaporator and insoluble residues were filtered off and washed with water. The aqueous phase was extracted with three 50 ml portions of diethylether. The combined diethylether phases were then extracted with 3 x 20 ml water. The aqueous phases were combined, and the solvent removed using a rotary evaporator to yield 72% (18.7 mg, 0.021 mmol) of pure product (total yield 56%). Single crystals were obtained by diffusion−crystallization from acetonitrile/diethyl ether at 4 °C.

4.1 Analytical Data

^{1}H NMR (300 MHz, CD_3CN): δ 5.86 (pseudo-t,^3J = 1.9 Hz, 4H, Cp-Se), 5.67 (cobaltocenium), 5.58 (pseudo-t,^3J = 1.9 Hz, 4H, Cp-Se), 5.50 (s, 10H, CcSe), 2.18 (H_2O), 1.94 (CD_3CN) ppm.

^{13}C NMR (75 MHz, CD_3CN): δ 118.69 (CD_3CN), 88.61 (Cp-Se ipso), 88.20 (Cp-Se), 87.84 (Cp), 84.77 (Cp-Se), 1.71 (CD_3CN) ppm.

MS (ESI+): *m/z* calc. 732.8063 (M^+), found 732.8049 (M^+).

UV-Vis (CH_3CN): λ_{max} 258 nm (ε 20156 L mol^{-1} cm^{-1}), 288 nm (ε 13942 L mol^{-1} cm^{-1}), 475 nm (ε 7960 L mol^{-1} cm^{-1}).

IR (ATR): 3123 w, 2957 m / 2926 m / 2857 m ($\nu_{C–H}$), 1724 m, 1454 w, 1408 m / 1401 w ($\nu_{C–C}$), 1269 m, 1120 m / 1070 m (δ_{C-C}), 1019 m (δ_{C-H}), 869 m (CH_{oop}), 814 s ($\nu_{P–F}$), 554 s ($\nu_{P–F}$), 501 m ($\nu_{Co–C}$), 452 s (δ_{Co-C}) cm^{-1}.

Melting point: 134.7°C (dec.)

4.2 Spectra

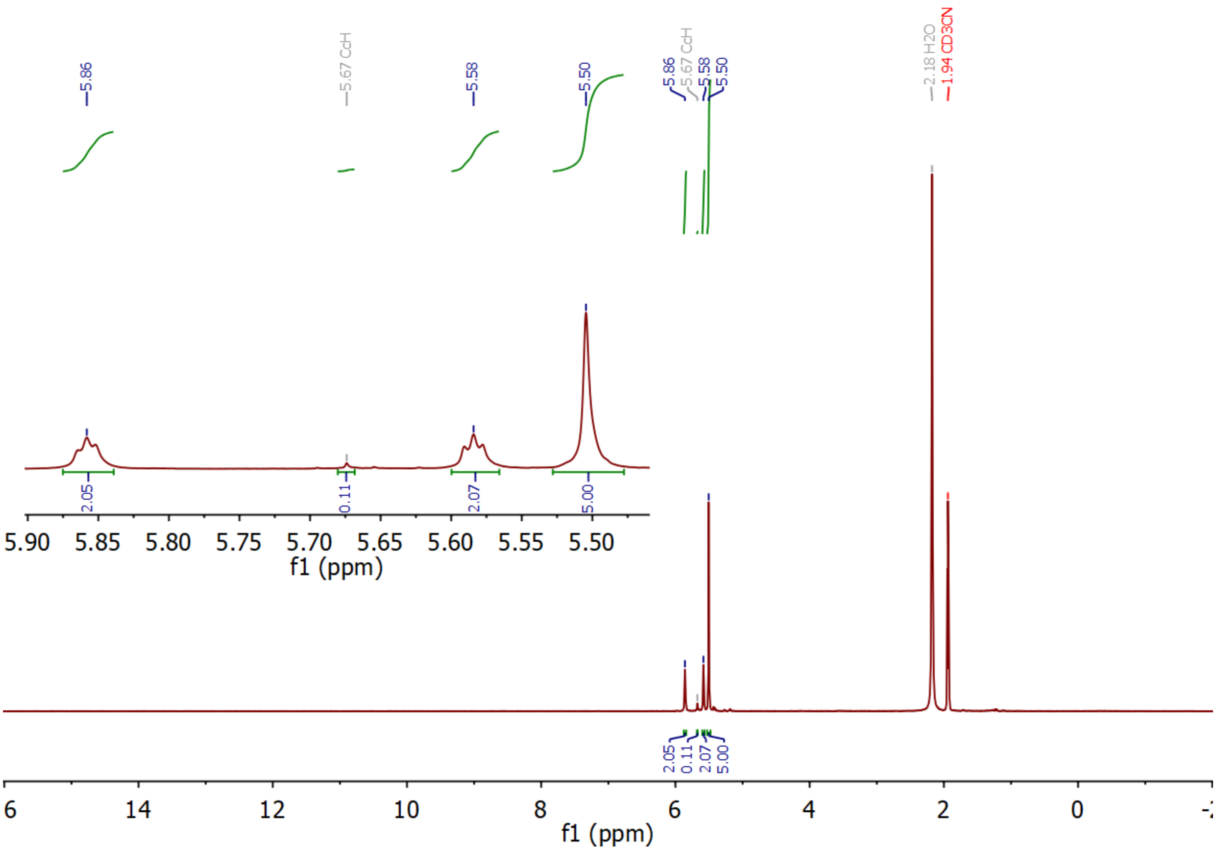

*Figure S15: ^{1}H NMR (300 MHz, CD_3CN) **4**.*

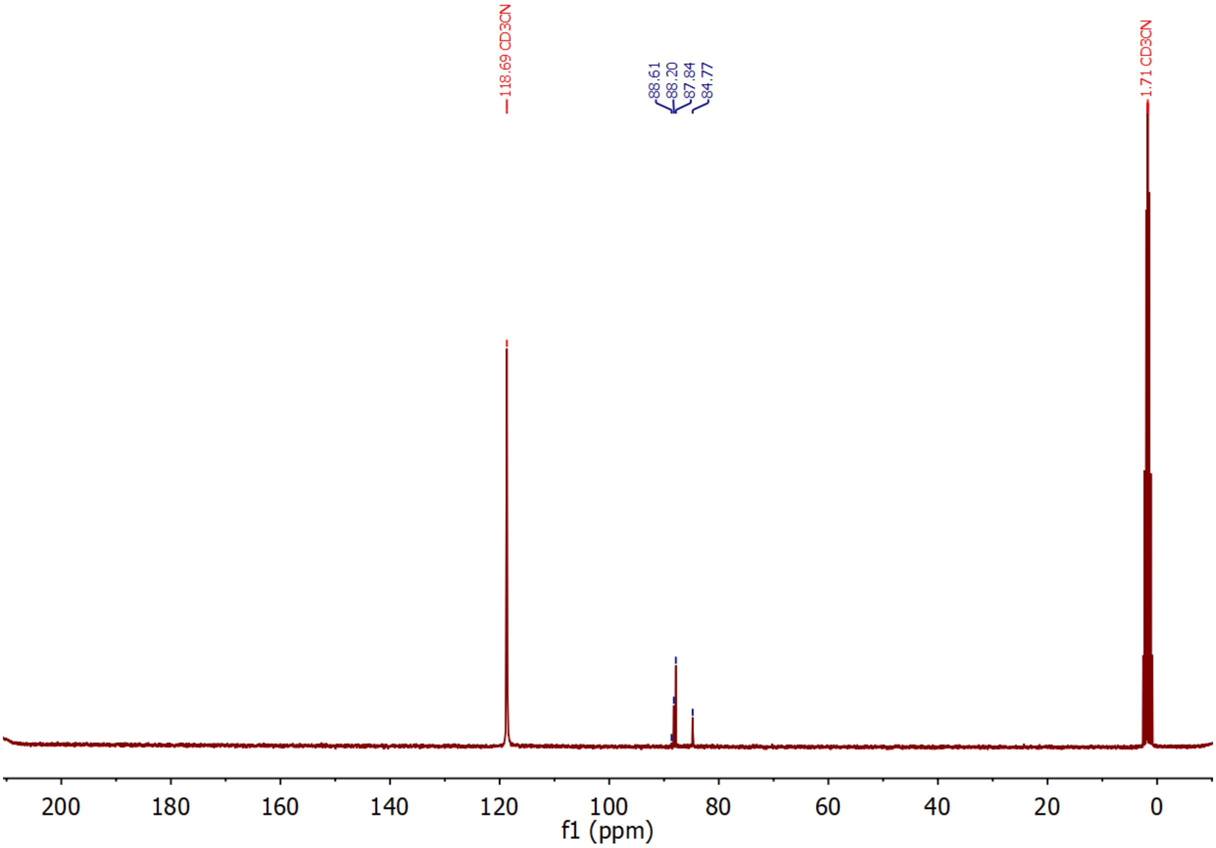

*Figure S16: ^{13}C NMR (75 MHz, CD_3CN) **4**.*

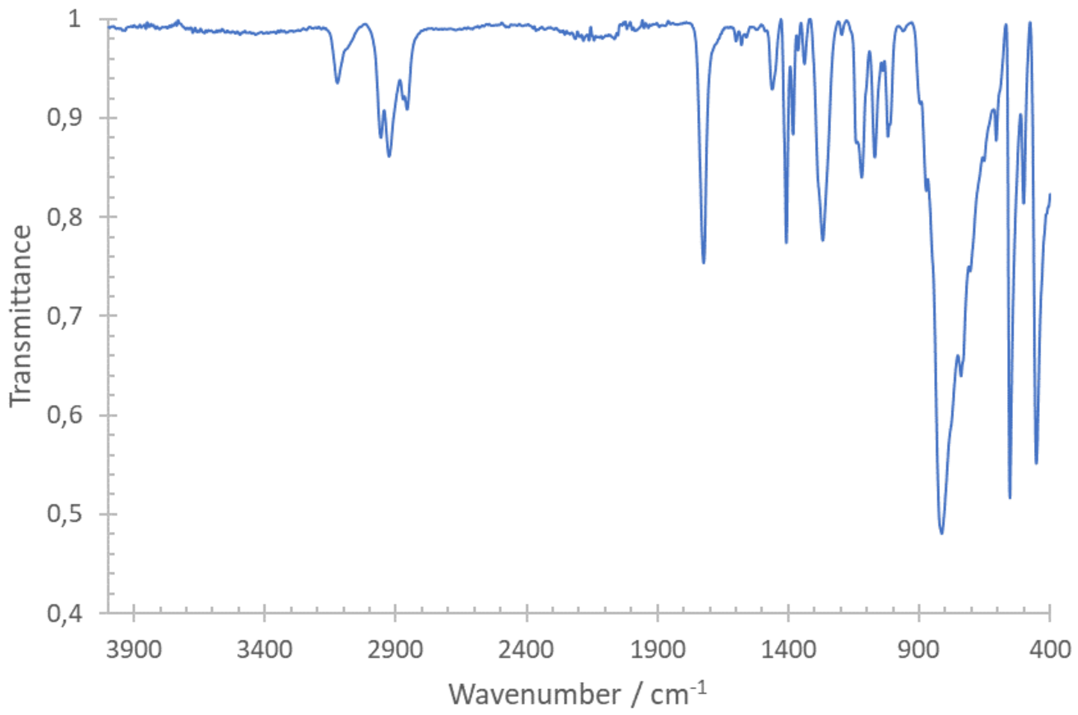

Figure S17: IR (ATR) ***4****.*

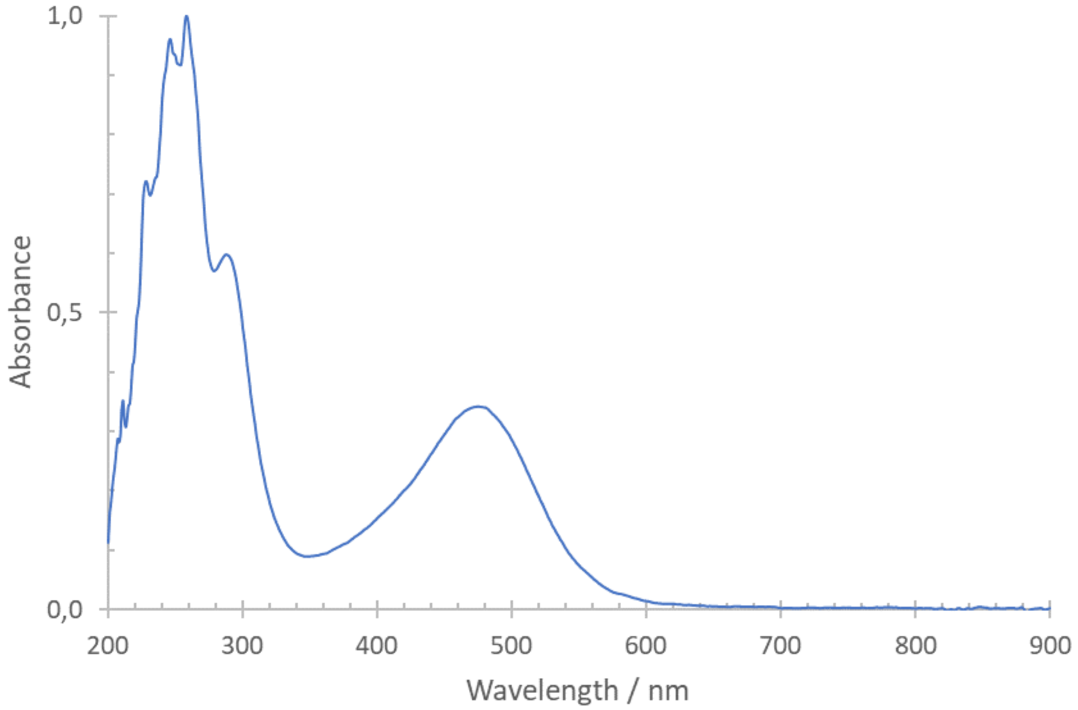

Figure S18: UV-Vis (CH_3CN) ***4****.*

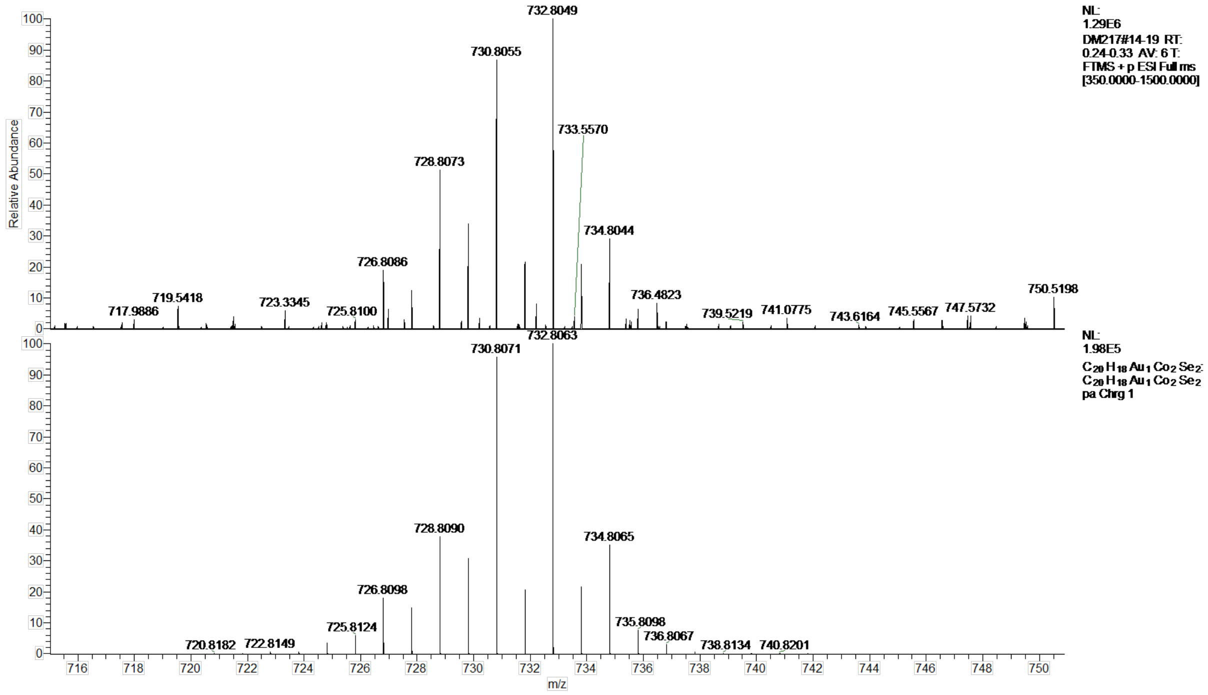

Figure S19: MS (ESI+) ***4****.*

4.3 Crystallographic Data

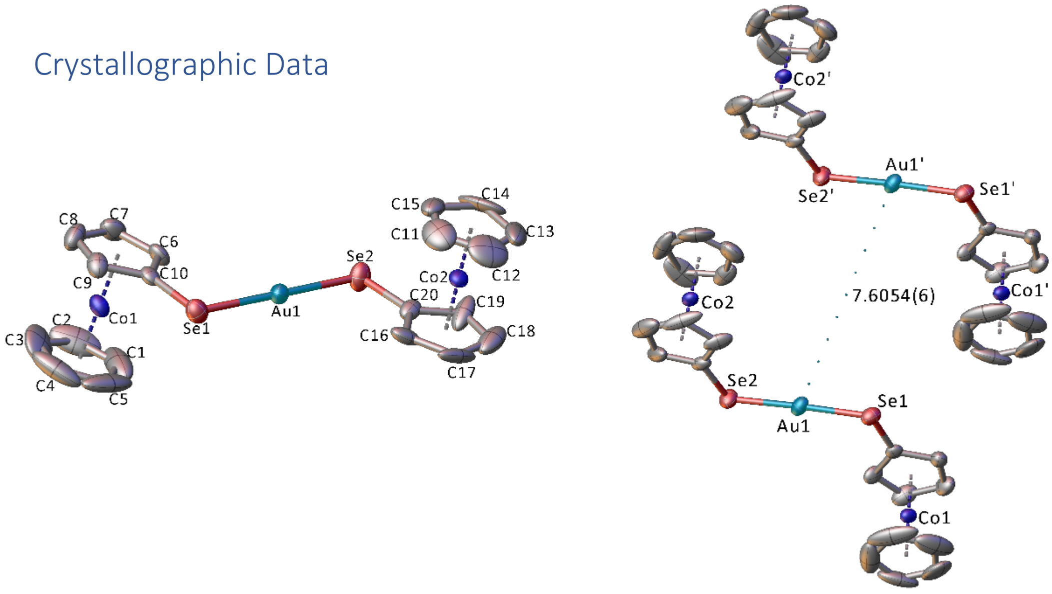

Counterion hexafluoridophosphate omitted for clarity.

Table S13. Crystal data and structure refinement for **4**.		
Empirical formula	$C_{20} H_{18} Au Co_2 F_6 Se_2$ x 0.85 CH_3CN	
Formula weight	910.96	
Temperature	173(2) K	
Wavelength	0.71073 Å	
Crystal system	Triclinic	
Space group	P-1 (no. 2)	
Unit cell dimensions	a = 7.6054(6) Å	α = 108.246(3)°
	b = 15.7412(13) Å	β = 91.428(3)°
	c = 22.889(2) Å	γ = 103.079(2)°
Volume	2521.4(4) Å3	
Z	4	
Density (calculated)	2.400 Mg/m^3	
Absorption coefficient	10.112 mm^{-1}	
F(000)	1707	
Crystal size	0.180 x 0.040 x 0.040 mm^3	
Theta range for data collection	2.657 to 24.999°	
Index ranges	$-9 \le h \le 9, -18 \le k \le 18, -27 \le l \le 27$	
Reflections collected	52090	
Independent reflections	8860 [R(int) = 0.0377]	
Completeness to theta = 24.999°	99.9 %	
Absorption correction	Semi-empirical from equivalents	
Max. and min. transmission	0.646 and 0.527	
Refinement method	Full-matrix least-squares on F^2	
Data / restraints / parameters	8860 / 0 / 638	
Goodness-of-fit on F^2	1.027	
Final R indices [I>2sigma(I)]	R1 = 0.0330, wR2 = 0.0760	
R indices (all data)	R1 = 0.0486, wR2 = 0.0816	
Extinction coefficient	n/a	
Largest diff. peak and hole	1.086 and -1.190 e.$Å^{-3}$	

Table S14. Atomic coordinates (x 10^4) and equivalent isotropic displacement parameters (Å^2 x 10^3) for **4**. U(eq) is defined as one third of the trace of the orthogonalized U_{ij} tensor.

	x	y	z	U(eq)
Au(1)	1057(1)	7042(1)	7321(1)	35(1)
Au(2)	6225(1)	7027(1)	7311(1)	37(1)
Co(1)	3641(1)	7001(1)	9284(1)	30(1)
Co(2)	-1457(1)	6994(1)	5346(1)	28(1)
Co(3)	7221(1)	4099(1)	7332(1)	26(1)
Co(4)	5129(1)	10022(1)	7461(1)	27(1)
Se(1)	678(1)	7696(1)	8393(1)	39(1)
Se(2)	1528(1)	6368(1)	6263(1)	44(1)
Se(3)	4495(1)	5457(1)	6964(1)	39(1)
Se(4)	7936(1)	8598(1)	7645(1)	54(1)
C(1)	6109(15)	7572(12)	9113(5)	104(5)
C(2)	6282(12)	7107(7)	9507(8)	97(4)
C(3)	5543(14)	7449(10)	10012(5)	95(4)
C(4)	4910(13)	8138(9)	9958(7)	105(5)
C(5)	5244(18)	8250(9)	9402(10)	126(7)
C(6)	2188(8)	6140(4)	8477(3)	35(2)
C(7)	2281(10)	5664(5)	8906(3)	50(2)
C(8)	1522(9)	6108(6)	9445(3)	50(2)
C(9)	931(8)	6834(5)	9345(3)	39(2)
C(10)	1301(7)	6862(4)	8741(3)	29(1)
C(11)	-3998(10)	6755(8)	5586(5)	72(3)
C(12)	-4014(12)	7016(8)	5086(6)	90(4)
C(13)	-3411(13)	6413(8)	4636(4)	79(3)
C(14)	-2943(12)	5730(6)	4873(5)	89(4)
C(15)	-3382(10)	6002(6)	5494(4)	54(2)
C(16)	204(9)	7924(5)	6086(4)	51(2)
C(17)	90(12)	8276(6)	5576(5)	79(4)
C(18)	669(11)	7711(8)	5055(5)	77(3)
C(19)	1190(9)	7039(7)	5229(4)	63(3)
C(20)	938(8)	7145(5)	5858(3)	36(2)
C(21)	9387(12)	4352(6)	6872(4)	65(3)
C(22)	9639(10)	3778(7)	7192(4)	63(2)
C(23)	8282(14)	2991(6)	7000(5)	70(3)
C(24)	7151(11)	3057(7)	6543(4)	75(3)
C(25)	7839(13)	3910(8)	6455(4)	67(3)
C(26)	7016(8)	5322(4)	7928(3)	31(1)
C(27)	7151(9)	4692(5)	8247(3)	40(2)
C(28)	5687(9)	3919(5)	8013(3)	41(2)
C(29)	4642(8)	4054(4)	7548(3)	32(1)
C(30)	5439(7)	4942(4)	7502(3)	25(1)
C(31)	3730(40)	9823(10)	8126(11)	211(15)
C(32)	2798(12)	10148(15)	7783(8)	169(10)
C(33)	3730(20)	10975(9)	7820(5)	95(4)

C(34)	5206(15)	11163(7)	8176(5)	88(4)
C(35)	5230(20)	10460(15)	8370(4)	144(8)
C(36)	5089(8)	8796(4)	6816(3)	32(1)
C(37)	4817(9)	9453(5)	6536(3)	39(2)
C(38)	6339(9)	10201(5)	6719(3)	45(2)
C(39)	7565(9)	10034(4)	7121(3)	42(2)
C(40)	6811(8)	9147(4)	7175(3)	29(1)
P(1)	448(3)	2920(1)	8806(1)	47(1)
P(2)	1585(2)	1358(1)	6239(1)	38(1)
F(1)	1438(9)	3481(4)	8398(3)	101(2)
F(2)	-507(12)	2354(4)	9208(2)	128(3)
F(3)	-346(10)	2087(5)	8223(3)	143(3)
F(4)	1410(9)	3714(5)	9410(3)	109(2)
F(5)	-1141(10)	3340(7)	8812(5)	170(4)
F(6)	2223(10)	2523(6)	8795(4)	139(3)
F(7)	3591(7)	1394(5)	6409(3)	102(2)
F(8)	-526(7)	1326(4)	6090(3)	95(2)
F(9)	2025(8)	2377(4)	6241(3)	92(2)
F(10)	1012(10)	360(4)	6293(4)	118(2)
F(11)	1319(9)	1723(4)	6945(2)	101(2)
F(12)	1632(12)	977(6)	5534(3)	139(3)
N(1)	-2147(12)	430(6)	9745(4)	87(3)
C(41)	-894(11)	435(5)	9493(4)	50(2)
C(42)	704(11)	424(6)	9169(4)	58(2)
N(2)	5480(20)	1413(15)	5040(8)	92(6)
C(43)	4960(20)	678(15)	5005(8)	56(4)
C(44)	4270(30)	-260(20)	4936(12)	94(9)
N(3)	6020(40)	4710(20)	9579(13)	108(9)
C(45)	5000	5000	10000	104(13)
C(46)	6020(40)	4710(20)	9579(13)	108(9)

Table S15. Bond lengths [Å] and angles [°] for **4**.

Au(1)-Se(2)	2.3912(8)
Au(1)-Se(1)	2.3979(7)
Au(2)-Se(4)	2.3883(8)
Au(2)-Se(3)	2.3909(8)
Co(1)-C(4)	1.982(9)
Co(1)-C(1)	1.994(9)
Co(1)-C(5)	1.998(10)
Co(1)-C(8)	2.009(7)
Co(1)-C(3)	2.013(8)
Co(1)-C(2)	2.016(8)
Co(1)-C(7)	2.019(7)
Co(1)-C(6)	2.030(6)
Co(1)-C(9)	2.032(6)
Co(1)-C(10)	2.075(6)
Co(2)-C(17)	1.992(7)
Co(2)-C(14)	1.994(7)
Co(2)-C(13)	2.005(7)
Co(2)-C(15)	2.005(7)
Co(2)-C(18)	2.008(8)
Co(2)-C(11)	2.008(8)
Co(2)-C(19)	2.026(7)
Co(2)-C(16)	2.027(6)
Co(2)-C(12)	2.030(8)
Co(2)-C(20)	2.067(6)
Co(3)-C(21)	2.008(7)
Co(3)-C(24)	2.014(7)
Co(3)-C(25)	2.017(7)
Co(3)-C(27)	2.018(6)

Co(3)-C(22)	2.022(7)
Co(3)-C(28)	2.023(6)
Co(3)-C(26)	2.024(6)
Co(3)-C(29)	2.027(6)
Co(3)-C(23)	2.027(7)
Co(3)-C(30)	2.065(5)
Co(4)-C(31)	1.944(11)
Co(4)-C(32)	1.964(9)
Co(4)-C(35)	1.970(10)
Co(4)-C(34)	2.005(8)
Co(4)-C(37)	2.007(6)
Co(4)-C(33)	2.014(8)
Co(4)-C(38)	2.017(7)
Co(4)-C(36)	2.022(6)
Co(4)-C(39)	2.025(6)
Co(4)-C(40)	2.059(6)
Se(1)-C(10)	1.872(6)
Se(2)-C(20)	1.870(6)
Se(3)-C(30)	1.880(6)
Se(4)-C(40)	1.873(6)
C(1)-C(2)	1.348(17)
C(1)-C(5)	1.381(18)
C(1)-H(1)	0.9500
C(2)-C(3)	1.312(16)
C(2)-H(2)	0.9500
C(3)-C(4)	1.320(17)
C(3)-H(3)	0.9500
C(4)-C(5)	1.360(19)
C(4)-H(4)	0.9500
C(5)-H(5)	0.9500
C(6)-C(7)	1.417(9)
C(6)-C(10)	1.431(8)
C(6)-H(6)	0.9500
C(7)-C(8)	1.423(11)
C(7)-H(7)	0.9500
C(8)-C(9)	1.398(10)
C(8)-H(8)	0.9500
C(9)-C(10)	1.431(9)
C(9)-H(9)	0.9500
C(11)-C(15)	1.330(12)
C(11)-C(12)	1.333(14)
C(11)-H(11)	0.9500
C(12)-C(13)	1.335(15)
C(12)-H(12)	0.9500
C(13)-C(14)	1.452(15)
C(13)-H(13)	0.9500
C(14)-C(15)	1.422(13)
C(14)-H(14)	0.9500
C(15)-H(15)	0.9500
C(16)-C(20)	1.417(10)
C(16)-C(17)	1.448(11)
C(16)-H(16)	0.9500
C(17)-C(18)	1.395(14)
C(17)-H(17)	0.9500
C(18)-C(19)	1.370(12)
C(18)-H(18)	0.9500
C(19)-C(20)	1.420(10)
C(19)-H(19)	0.9500
C(21)-C(22)	1.369(12)
C(21)-C(25)	1.391(12)
C(21)-H(21)	0.9500
C(22)-C(23)	1.361(12)
C(22)-H(22)	0.9500
C(23)-C(24)	1.381(13)
C(23)-H(23)	0.9500
C(24)-C(25)	1.405(13)
C(24)-H(24)	0.9500
C(25)-H(25)	0.9500
C(26)-C(30)	1.415(8)
C(26)-C(27)	1.422(9)
C(26)-H(26)	0.9500
C(27)-C(28)	1.399(10)
C(27)-H(27)	0.9500
C(28)-C(29)	1.408(9)
C(28)-H(28)	0.9500
C(29)-C(30)	1.431(8)
C(29)-H(29)	0.9500
C(31)-C(35)	1.31(3)
C(31)-C(32)	1.33(3)
C(31)-H(31)	0.9500
C(32)-C(33)	1.31(2)
C(32)-H(32)	0.9500
C(33)-C(34)	1.292(15)
C(33)-H(33)	0.9500
C(34)-C(35)	1.319(18)
C(34)-H(34)	0.9500
C(35)-H(35)	0.9500
C(36)-C(37)	1.424(9)
C(36)-C(40)	1.425(8)
C(36)-H(36)	0.9500
C(37)-C(38)	1.397(10)
C(37)-H(37)	0.9500
C(38)-C(39)	1.416(10)
C(38)-H(38)	0.9500

C(39)-C(40)	1.430(9)
C(39)-H(39)	0.9500
P(1)-F(5)	1.502(6)
P(1)-F(3)	1.540(6)
P(1)-F(2)	1.556(5)
P(1)-F(4)	1.570(6)
P(1)-F(1)	1.572(5)
P(1)-F(6)	1.607(7)
P(2)-F(12)	1.540(6)
P(2)-F(7)	1.550(5)
P(2)-F(9)	1.561(5)
P(2)-F(11)	1.572(5)
P(2)-F(10)	1.578(6)
P(2)-F(8)	1.619(5)
N(1)-C(41)	1.125(10)
C(41)-C(42)	1.440(11)
C(42)-H(42A)	0.9800
C(42)-H(42B)	0.9800
C(42)-H(42C)	0.9800
N(2)-C(43)	1.11(2)
C(43)-C(44)	1.41(4)
C(44)-H(44A)	0.9800
C(44)-H(44B)	0.9800
C(44)-H(44C)	0.9800
N(3)-C(45)	1.29(3)
C(45)-C(46)#1	1.29(3)
C(45)-N(3)#1	1.29(3)
Se(2)-Au(1)-Se(1)	177.81(2)
Se(4)-Au(2)-Se(3)	179.16(3)
C(4)-Co(1)-C(1)	66.4(5)
C(4)-Co(1)-C(5)	39.9(6)
C(1)-Co(1)-C(5)	40.5(6)
C(4)-Co(1)-C(8)	120.5(5)
C(1)-Co(1)-C(8)	162.3(7)
C(5)-Co(1)-C(8)	154.8(8)
C(4)-Co(1)-C(3)	38.6(5)
C(1)-Co(1)-C(3)	65.6(5)
C(5)-Co(1)-C(3)	66.3(5)
C(8)-Co(1)-C(3)	108.5(4)
C(4)-Co(1)-C(2)	64.5(4)
C(1)-Co(1)-C(2)	39.3(5)
C(5)-Co(1)-C(2)	66.3(5)
C(8)-Co(1)-C(2)	125.9(5)
C(3)-Co(1)-C(2)	38.0(5)
C(4)-Co(1)-C(7)	156.1(6)
C(1)-Co(1)-C(7)	126.1(6)

C(5)-Co(1)-C(7)	162.5(8)
C(8)-Co(1)-C(7)	41.4(3)
C(3)-Co(1)-C(7)	122.9(5)
C(2)-Co(1)-C(7)	110.7(4)
C(4)-Co(1)-C(6)	161.7(6)
C(1)-Co(1)-C(6)	109.3(3)
C(5)-Co(1)-C(6)	125.1(6)
C(8)-Co(1)-C(6)	69.3(3)
C(3)-Co(1)-C(6)	157.9(5)
C(2)-Co(1)-C(6)	124.4(4)
C(7)-Co(1)-C(6)	41.0(3)
C(4)-Co(1)-C(9)	107.7(4)
C(1)-Co(1)-C(9)	156.8(7)
C(5)-Co(1)-C(9)	120.4(6)
C(8)-Co(1)-C(9)	40.5(3)
C(3)-Co(1)-C(9)	124.8(4)
C(2)-Co(1)-C(9)	160.5(6)
C(7)-Co(1)-C(9)	68.6(3)
C(6)-Co(1)-C(9)	68.8(3)
C(4)-Co(1)-C(10)	125.0(4)
C(1)-Co(1)-C(10)	122.6(5)
C(5)-Co(1)-C(10)	107.5(3)
C(8)-Co(1)-C(10)	68.7(3)
C(3)-Co(1)-C(10)	160.6(5)
C(2)-Co(1)-C(10)	158.5(6)
C(7)-Co(1)-C(10)	68.6(3)
C(6)-Co(1)-C(10)	40.8(2)
C(9)-Co(1)-C(10)	40.8(2)
C(17)-Co(2)-C(14)	163.6(5)
C(17)-Co(2)-C(13)	124.8(4)
C(14)-Co(2)-C(13)	42.6(4)
C(17)-Co(2)-C(15)	153.1(5)
C(14)-Co(2)-C(15)	41.7(4)
C(13)-Co(2)-C(15)	68.4(3)
C(17)-Co(2)-C(18)	40.8(4)
C(14)-Co(2)-C(18)	125.8(5)
C(13)-Co(2)-C(18)	107.6(4)
C(15)-Co(2)-C(18)	165.4(5)
C(17)-Co(2)-C(11)	120.6(5)
C(14)-Co(2)-C(11)	67.2(4)
C(13)-Co(2)-C(11)	65.0(4)
C(15)-Co(2)-C(11)	38.7(4)
C(18)-Co(2)-C(11)	153.6(5)
C(17)-Co(2)-C(19)	67.0(4)
C(14)-Co(2)-C(19)	108.8(4)
C(13)-Co(2)-C(19)	122.0(4)
C(15)-Co(2)-C(19)	129.5(4)

C(18)-Co(2)-C(19)	39.7(4)
C(11)-Co(2)-C(19)	166.0(4)
C(17)-Co(2)-C(16)	42.2(3)
C(14)-Co(2)-C(16)	152.8(5)
C(13)-Co(2)-C(16)	162.2(5)
C(15)-Co(2)-C(16)	118.0(3)
C(18)-Co(2)-C(16)	70.3(4)
C(11)-Co(2)-C(16)	108.6(4)
C(19)-Co(2)-C(16)	68.3(4)
C(17)-Co(2)-C(12)	107.8(4)
C(14)-Co(2)-C(12)	68.6(5)
C(13)-Co(2)-C(12)	38.6(4)
C(15)-Co(2)-C(12)	66.6(4)
C(18)-Co(2)-C(12)	119.7(4)
C(11)-Co(2)-C(12)	38.5(4)
C(19)-Co(2)-C(12)	154.3(5)
C(16)-Co(2)-C(12)	126.0(4)
C(17)-Co(2)-C(20)	68.5(3)
C(14)-Co(2)-C(20)	119.5(4)
C(13)-Co(2)-C(20)	156.5(5)
C(15)-Co(2)-C(20)	108.9(3)
C(18)-Co(2)-C(20)	68.8(3)
C(11)-Co(2)-C(20)	128.4(4)
C(19)-Co(2)-C(20)	40.6(3)
C(16)-Co(2)-C(20)	40.5(3)
C(12)-Co(2)-C(20)	163.6(5)
C(21)-Co(3)-C(24)	67.7(4)
C(21)-Co(3)-C(25)	40.5(4)
C(24)-Co(3)-C(25)	40.8(4)
C(21)-Co(3)-C(27)	124.7(3)
C(24)-Co(3)-C(27)	156.2(4)
C(25)-Co(3)-C(27)	161.3(4)
C(21)-Co(3)-C(22)	39.7(4)
C(24)-Co(3)-C(22)	66.6(4)
C(25)-Co(3)-C(22)	67.2(3)
C(27)-Co(3)-C(22)	108.7(3)
C(21)-Co(3)-C(28)	161.3(4)
C(24)-Co(3)-C(28)	121.6(4)
C(25)-Co(3)-C(28)	156.8(4)
C(27)-Co(3)-C(28)	40.5(3)
C(22)-Co(3)-C(28)	125.6(3)
C(21)-Co(3)-C(26)	107.6(3)
C(24)-Co(3)-C(26)	161.6(4)
C(25)-Co(3)-C(26)	124.1(4)
C(27)-Co(3)-C(26)	41.2(2)
C(22)-Co(3)-C(26)	122.0(3)
C(28)-Co(3)-C(26)	68.6(3)
C(21)-Co(3)-C(29)	156.7(4)
C(24)-Co(3)-C(29)	108.2(3)
C(25)-Co(3)-C(29)	121.3(3)
C(27)-Co(3)-C(29)	68.5(3)
C(22)-Co(3)-C(29)	161.8(4)
C(28)-Co(3)-C(29)	40.7(3)
C(26)-Co(3)-C(29)	68.6(2)
C(21)-Co(3)-C(23)	67.1(4)
C(24)-Co(3)-C(23)	40.0(4)
C(25)-Co(3)-C(23)	67.8(4)
C(27)-Co(3)-C(23)	121.4(3)
C(22)-Co(3)-C(23)	39.3(4)
C(28)-Co(3)-C(23)	108.6(3)
C(26)-Co(3)-C(23)	156.5(4)
C(29)-Co(3)-C(23)	125.6(3)
C(21)-Co(3)-C(30)	121.2(3)
C(24)-Co(3)-C(30)	125.3(3)
C(25)-Co(3)-C(30)	107.4(3)
C(27)-Co(3)-C(30)	68.7(2)
C(22)-Co(3)-C(30)	156.5(3)
C(28)-Co(3)-C(30)	68.6(2)
C(26)-Co(3)-C(30)	40.5(2)
C(29)-Co(3)-C(30)	40.9(2)
C(23)-Co(3)-C(30)	162.2(4)
C(31)-Co(4)-C(32)	39.8(8)
C(31)-Co(4)-C(35)	39.1(8)
C(32)-Co(4)-C(35)	65.3(6)
C(31)-Co(4)-C(34)	64.9(6)
C(32)-Co(4)-C(34)	63.9(5)
C(35)-Co(4)-C(34)	38.7(5)
C(31)-Co(4)-C(37)	134.8(9)
C(32)-Co(4)-C(37)	111.1(5)
C(35)-Co(4)-C(37)	173.6(8)
C(34)-Co(4)-C(37)	145.5(5)
C(31)-Co(4)-C(33)	65.4(6)
C(32)-Co(4)-C(33)	38.4(6)
C(35)-Co(4)-C(33)	64.5(4)
C(34)-Co(4)-C(33)	37.5(4)
C(37)-Co(4)-C(33)	116.3(4)
C(31)-Co(4)-C(38)	174.2(11)
C(32)-Co(4)-C(38)	135.1(8)
C(35)-Co(4)-C(38)	145.6(7)
C(34)-Co(4)-C(38)	116.8(4)
C(37)-Co(4)-C(38)	40.6(3)
C(33)-Co(4)-C(38)	112.3(4)
C(31)-Co(4)-C(36)	109.9(5)
C(32)-Co(4)-C(36)	115.6(5)

C(35)-Co(4)-C(36)	134.4(6)
C(34)-Co(4)-C(36)	173.1(5)
C(37)-Co(4)-C(36)	41.4(2)
C(33)-Co(4)-C(36)	146.1(5)
C(38)-Co(4)-C(36)	68.8(3)
C(31)-Co(4)-C(39)	144.4(10)
C(32)-Co(4)-C(39)	173.6(8)
C(35)-Co(4)-C(39)	115.1(5)
C(34)-Co(4)-C(39)	112.1(4)
C(37)-Co(4)-C(39)	69.1(3)
C(33)-Co(4)-C(39)	135.5(5)
C(38)-Co(4)-C(39)	41.0(3)
C(36)-Co(4)-C(39)	69.0(3)
C(31)-Co(4)-C(40)	114.2(6)
C(32)-Co(4)-C(40)	145.4(8)
C(35)-Co(4)-C(40)	110.5(3)
C(34)-Co(4)-C(40)	135.6(4)
C(37)-Co(4)-C(40)	69.2(3)
C(33)-Co(4)-C(40)	173.1(5)
C(38)-Co(4)-C(40)	68.8(3)
C(36)-Co(4)-C(40)	40.8(2)
C(39)-Co(4)-C(40)	41.0(2)
C(10)-Se(1)-Au(1)	102.85(18)
C(20)-Se(2)-Au(1)	105.3(2)
C(30)-Se(3)-Au(2)	103.45(18)
C(40)-Se(4)-Au(2)	104.29(18)
C(2)-C(1)-C(5)	107.1(11)
C(2)-C(1)-Co(1)	71.2(6)
C(5)-C(1)-Co(1)	69.9(6)
C(2)-C(1)-H(1)	126.4
C(5)-C(1)-H(1)	126.4
Co(1)-C(1)-H(1)	124.1
C(3)-C(2)-C(1)	109.5(11)
C(3)-C(2)-Co(1)	70.9(5)
C(1)-C(2)-Co(1)	69.5(6)
C(3)-C(2)-H(2)	125.3
C(1)-C(2)-H(2)	125.3
Co(1)-C(2)-H(2)	126.0
C(2)-C(3)-C(4)	108.3(12)
C(2)-C(3)-Co(1)	71.1(6)
C(4)-C(3)-Co(1)	69.5(5)
C(2)-C(3)-H(3)	125.8
C(4)-C(3)-H(3)	125.8
Co(1)-C(3)-H(3)	125.2
C(3)-C(4)-C(5)	109.9(11)
C(3)-C(4)-Co(1)	71.9(6)
C(5)-C(4)-Co(1)	70.7(6)
C(3)-C(4)-H(4)	125.1
C(5)-C(4)-H(4)	125.1
Co(1)-C(4)-H(4)	123.9
C(4)-C(5)-C(1)	105.2(10)
C(4)-C(5)-Co(1)	69.4(6)
C(1)-C(5)-Co(1)	69.6(6)
C(4)-C(5)-H(5)	127.4
C(1)-C(5)-H(5)	127.4
Co(1)-C(5)-H(5)	125.2
C(7)-C(6)-C(10)	108.2(6)
C(7)-C(6)-Co(1)	69.1(4)
C(10)-C(6)-Co(1)	71.3(3)
C(7)-C(6)-H(6)	125.9
C(10)-C(6)-H(6)	125.9
Co(1)-C(6)-H(6)	125.3
C(6)-C(7)-C(8)	108.1(6)
C(6)-C(7)-Co(1)	69.9(4)
C(8)-C(7)-Co(1)	69.0(4)
C(6)-C(7)-H(7)	126.0
C(8)-C(7)-H(7)	126.0
Co(1)-C(7)-H(7)	126.7
C(9)-C(8)-C(7)	108.0(6)
C(9)-C(8)-Co(1)	70.6(4)
C(7)-C(8)-Co(1)	69.7(4)
C(9)-C(8)-H(8)	126.0
C(7)-C(8)-H(8)	126.0
Co(1)-C(8)-H(8)	125.3
C(8)-C(9)-C(10)	109.2(6)
C(8)-C(9)-Co(1)	68.9(4)
C(10)-C(9)-Co(1)	71.3(3)
C(8)-C(9)-H(9)	125.4
C(10)-C(9)-H(9)	125.4
Co(1)-C(9)-H(9)	126.0
C(9)-C(10)-C(6)	106.6(6)
C(9)-C(10)-Se(1)	124.5(5)
C(6)-C(10)-Se(1)	129.0(5)
C(9)-C(10)-Co(1)	68.0(3)
C(6)-C(10)-Co(1)	67.9(3)
Se(1)-C(10)-Co(1)	129.3(3)
C(15)-C(11)-C(12)	112.7(10)
C(15)-C(11)-Co(2)	70.5(5)
C(12)-C(11)-Co(2)	71.6(5)
C(15)-C(11)-H(11)	123.7
C(12)-C(11)-H(11)	123.7
Co(2)-C(11)-H(11)	125.8
C(11)-C(12)-C(13)	107.8(10)
C(11)-C(12)-Co(2)	69.8(5)

C(13)-C(12)-Co(2)	69.6(5)		C(16)-C(20)-Se(2)	129.7(5)
C(11)-C(12)-H(12)	126.1		C(19)-C(20)-Se(2)	123.6(6)
C(13)-C(12)-H(12)	126.1		C(16)-C(20)-Co(2)	68.3(3)
Co(2)-C(12)-H(12)	126.0		C(19)-C(20)-Co(2)	68.1(4)
C(12)-C(13)-C(14)	108.9(9)		Se(2)-C(20)-Co(2)	128.7(3)
C(12)-C(13)-Co(2)	71.7(5)		C(22)-C(21)-C(25)	108.0(8)
C(14)-C(13)-Co(2)	68.3(5)		C(22)-C(21)-Co(3)	70.7(4)
C(12)-C(13)-H(13)	125.5		C(25)-C(21)-Co(3)	70.1(4)
C(14)-C(13)-H(13)	125.5		C(22)-C(21)-H(21)	126.0
Co(2)-C(13)-H(13)	126.0		C(25)-C(21)-H(21)	126.0
C(15)-C(14)-C(13)	103.3(8)		Co(3)-C(21)-H(21)	124.8
C(15)-C(14)-Co(2)	69.6(4)		C(23)-C(22)-C(21)	109.5(8)
C(13)-C(14)-Co(2)	69.1(4)		C(23)-C(22)-Co(3)	70.6(4)
C(15)-C(14)-H(14)	128.3		C(21)-C(22)-Co(3)	69.6(4)
C(13)-C(14)-H(14)	128.3		C(23)-C(22)-H(22)	125.2
Co(2)-C(14)-H(14)	124.7		C(21)-C(22)-H(22)	125.2
C(11)-C(15)-C(14)	107.2(8)		Co(3)-C(22)-H(22)	126.2
C(11)-C(15)-Co(2)	70.8(5)		C(22)-C(23)-C(24)	107.8(8)
C(14)-C(15)-Co(2)	68.8(4)		C(22)-C(23)-Co(3)	70.2(4)
C(11)-C(15)-H(15)	126.4		C(24)-C(23)-Co(3)	69.5(5)
C(14)-C(15)-H(15)	126.4		C(22)-C(23)-H(23)	126.1
Co(2)-C(15)-H(15)	125.7		C(24)-C(23)-H(23)	126.1
C(20)-C(16)-C(17)	105.7(8)		Co(3)-C(23)-H(23)	125.8
C(20)-C(16)-Co(2)	71.3(4)		C(23)-C(24)-C(25)	108.1(8)
C(17)-C(16)-Co(2)	67.6(4)		C(23)-C(24)-Co(3)	70.5(5)
C(20)-C(16)-H(16)	127.1		C(25)-C(24)-Co(3)	69.7(4)
C(17)-C(16)-H(16)	127.1		C(23)-C(24)-H(24)	126.0
Co(2)-C(16)-H(16)	125.6		C(25)-C(24)-H(24)	126.0
C(18)-C(17)-C(16)	109.6(8)		Co(3)-C(24)-H(24)	125.4
C(18)-C(17)-Co(2)	70.2(5)		C(21)-C(25)-C(24)	106.5(8)
C(16)-C(17)-Co(2)	70.2(4)		C(21)-C(25)-Co(3)	69.4(4)
C(18)-C(17)-H(17)	125.2		C(24)-C(25)-Co(3)	69.5(5)
C(16)-C(17)-H(17)	125.2		C(21)-C(25)-H(25)	126.7
Co(2)-C(17)-H(17)	126.0		C(24)-C(25)-H(25)	126.7
C(19)-C(18)-C(17)	106.7(8)		Co(3)-C(25)-H(25)	125.9
C(19)-C(18)-Co(2)	70.9(4)		C(30)-C(26)-C(27)	108.6(6)
C(17)-C(18)-Co(2)	69.0(5)		C(30)-C(26)-Co(3)	71.3(3)
C(19)-C(18)-H(18)	126.6		C(27)-C(26)-Co(3)	69.2(4)
C(17)-C(18)-H(18)	126.6		C(30)-C(26)-H(26)	125.7
Co(2)-C(18)-H(18)	125.1		C(27)-C(26)-H(26)	125.7
C(18)-C(19)-C(20)	111.2(9)		Co(3)-C(26)-H(26)	125.3
C(18)-C(19)-Co(2)	69.4(5)		C(28)-C(27)-C(26)	107.9(6)
C(20)-C(19)-Co(2)	71.3(4)		C(28)-C(27)-Co(3)	69.9(4)
C(18)-C(19)-H(19)	124.4		C(26)-C(27)-Co(3)	69.6(4)
C(20)-C(19)-H(19)	124.4		C(28)-C(27)-H(27)	126.0
Co(2)-C(19)-H(19)	126.5		C(26)-C(27)-H(27)	126.0
C(16)-C(20)-C(19)	106.7(6)		Co(3)-C(27)-H(27)	126.0

C(27)-C(28)-C(29)	108.4(6)
C(27)-C(28)-Co(3)	69.6(4)
C(29)-C(28)-Co(3)	69.8(4)
C(27)-C(28)-H(28)	125.8
C(29)-C(28)-H(28)	125.8
Co(3)-C(28)-H(28)	126.4
C(28)-C(29)-C(30)	108.4(6)
C(28)-C(29)-Co(3)	69.5(4)
C(30)-C(29)-Co(3)	71.0(3)
C(28)-C(29)-H(29)	125.8
C(30)-C(29)-H(29)	125.8
Co(3)-C(29)-H(29)	125.3
C(26)-C(30)-C(29)	106.6(5)
C(26)-C(30)-Se(3)	128.7(4)
C(29)-C(30)-Se(3)	124.6(4)
C(26)-C(30)-Co(3)	68.2(3)
C(29)-C(30)-Co(3)	68.1(3)
Se(3)-C(30)-Co(3)	128.4(3)
C(35)-C(31)-C(32)	107.2(12)
C(35)-C(31)-Co(4)	71.6(7)
C(32)-C(31)-Co(4)	71.0(8)
C(35)-C(31)-H(31)	126.4
C(32)-C(31)-H(31)	126.4
Co(4)-C(31)-H(31)	122.8
C(33)-C(32)-C(31)	108.3(13)
C(33)-C(32)-Co(4)	72.9(6)
C(31)-C(32)-Co(4)	69.3(7)
C(33)-C(32)-H(32)	125.8
C(31)-C(32)-H(32)	125.8
Co(4)-C(32)-H(32)	123.6
C(34)-C(33)-C(32)	107.7(12)
C(34)-C(33)-Co(4)	70.9(5)
C(32)-C(33)-Co(4)	68.8(6)
C(34)-C(33)-H(33)	126.1
C(32)-C(33)-H(33)	126.1
Co(4)-C(33)-H(33)	125.8
C(33)-C(34)-C(35)	109.2(13)
C(33)-C(34)-Co(4)	71.6(5)
C(35)-C(34)-Co(4)	69.2(6)
C(33)-C(34)-H(34)	125.4
C(35)-C(34)-H(34)	125.4
Co(4)-C(34)-H(34)	125.3
C(31)-C(35)-C(34)	107.6(13)
C(31)-C(35)-Co(4)	69.4(8)
C(34)-C(35)-Co(4)	72.0(6)
C(31)-C(35)-H(35)	126.2
C(34)-C(35)-H(35)	126.2

Co(4)-C(35)-H(35)	124.0
C(37)-C(36)-C(40)	108.3(5)
C(37)-C(36)-Co(4)	68.7(4)
C(40)-C(36)-Co(4)	71.0(3)
C(37)-C(36)-H(36)	125.9
C(40)-C(36)-H(36)	125.9
Co(4)-C(36)-H(36)	126.0
C(38)-C(37)-C(36)	108.0(6)
C(38)-C(37)-Co(4)	70.1(4)
C(36)-C(37)-Co(4)	69.9(4)
C(38)-C(37)-H(37)	126.0
C(36)-C(37)-H(37)	126.0
Co(4)-C(37)-H(37)	125.7
C(37)-C(38)-C(39)	108.7(6)
C(37)-C(38)-Co(4)	69.3(4)
C(39)-C(38)-Co(4)	69.8(4)
C(37)-C(38)-H(38)	125.6
C(39)-C(38)-H(38)	125.6
Co(4)-C(38)-H(38)	126.8
C(38)-C(39)-C(40)	108.1(6)
C(38)-C(39)-Co(4)	69.2(4)
C(40)-C(39)-Co(4)	70.8(3)
C(38)-C(39)-H(39)	126.0
C(40)-C(39)-H(39)	126.0
Co(4)-C(39)-H(39)	125.7
C(36)-C(40)-C(39)	106.8(5)
C(36)-C(40)-Se(4)	128.5(5)
C(39)-C(40)-Se(4)	124.7(5)
C(36)-C(40)-Co(4)	68.2(3)
C(39)-C(40)-Co(4)	68.3(3)
Se(4)-C(40)-Co(4)	127.8(3)
F(5)-P(1)-F(3)	94.5(6)
F(5)-P(1)-F(2)	89.8(5)
F(3)-P(1)-F(2)	89.1(3)
F(5)-P(1)-F(4)	91.4(5)
F(3)-P(1)-F(4)	174.0(5)
F(2)-P(1)-F(4)	89.7(4)
F(5)-P(1)-F(1)	91.2(4)
F(3)-P(1)-F(1)	90.6(3)
F(2)-P(1)-F(1)	179.0(4)
F(4)-P(1)-F(1)	90.5(3)
F(5)-P(1)-F(6)	176.7(5)
F(3)-P(1)-F(6)	87.1(5)
F(2)-P(1)-F(6)	93.2(5)
F(4)-P(1)-F(6)	87.1(4)
F(1)-P(1)-F(6)	85.8(4)
F(12)-P(2)-F(7)	95.7(4)

F(12)-P(2)-F(9)	92.4(4)
F(7)-P(2)-F(9)	93.6(3)
F(12)-P(2)-F(11)	174.1(4)
F(7)-P(2)-F(11)	90.0(4)
F(9)-P(2)-F(11)	88.5(3)
F(12)-P(2)-F(10)	92.5(4)
F(7)-P(2)-F(10)	89.3(3)
F(9)-P(2)-F(10)	174.0(4)
F(11)-P(2)-F(10)	86.3(4)
F(12)-P(2)-F(8)	86.5(4)
F(7)-P(2)-F(8)	177.7(3)
F(9)-P(2)-F(8)	86.8(3)
F(11)-P(2)-F(8)	87.8(3)
F(10)-P(2)-F(8)	90.1(3)
N(1)-C(41)-C(42)	179.0(9)
C(41)-C(42)-H(42A)	109.5
C(41)-C(42)-H(42B)	109.5
H(42A)-C(42)-H(42B)	109.5
C(41)-C(42)-H(42C)	109.5
H(42A)-C(42)-H(42C)	109.5
H(42B)-C(42)-H(42C)	109.5
N(2)-C(43)-C(44)	177(3)
C(43)-C(44)-H(44A)	109.5
C(43)-C(44)-H(44B)	109.5
H(44A)-C(44)-H(44B)	109.5
C(43)-C(44)-H(44C)	109.5
H(44A)-C(44)-H(44C)	109.5
H(44B)-C(44)-H(44C)	109.5
C(46)#1-C(45)-N(3)#1	0.0
C(46)#1-C(45)-N(3)	180.0
N(3)#1-C(45)-N(3)	180(2)

Symmetry transformations used to generate equivalent atoms:
#1 -x + 1, -y + 1, -z + 2

Table S16. Anisotropic displacement parameters (Å^2 x 10^3) for **4**. The anisotropic displacement factor exponent takes the form: $-2\pi^2$ [h^2 a^{*2} U^{11} + ... + 2 h k a* b* U^{12}]

	U^{11}	U^{22}	U^{33}	U^{23}	U^{13}	U^{12}
Au(1)	43(1)	33(1)	29(1)	13(1)	-2(1)	3(1)
Au(2)	55(1)	27(1)	32(1)	7(1)	-3(1)	21(1)
Co(1)	26(1)	35(1)	25(1)	3(1)	-1(1)	11(1)
Co(2)	22(1)	30(1)	28(1)	10(1)	-3(1)	0(1)
Co(3)	20(1)	22(1)	35(1)	7(1)	2(1)	8(1)
Co(4)	21(1)	25(1)	36(1)	11(1)	3(1)	6(1)
Se(1)	43(1)	39(1)	36(1)	10(1)	-2(1)	15(1)
Se(2)	51(1)	54(1)	38(1)	22(1)	7(1)	27(1)
Se(3)	39(1)	38(1)	39(1)	9(1)	-6(1)	17(1)
Se(4)	47(1)	38(1)	72(1)	8(1)	-21(1)	18(1)
C(1)	57(7)	155(13)	47(6)	11(7)	6(5)	-49(7)
C(2)	30(5)	69(7)	164(13)	-7(8)	-17(6)	23(5)
C(3)	59(6)	146(12)	68(7)	43(8)	-40(5)	-5(7)
C(4)	46(6)	83(9)	122(10)	-54(8)	-27(6)	18(6)
C(5)	80(9)	64(8)	223(18)	84(11)	-97(11)	-42(6)
C(6)	44(4)	26(3)	26(3)	-4(3)	-6(3)	11(3)
C(7)	64(5)	29(4)	53(5)	12(3)	-14(4)	9(3)
C(8)	45(4)	68(5)	48(4)	38(4)	2(3)	6(4)
C(9)	27(3)	57(5)	34(4)	16(3)	3(3)	9(3)
C(10)	22(3)	32(3)	27(3)	4(3)	-6(2)	1(2)
C(11)	31(4)	96(8)	79(7)	19(6)	15(4)	7(5)
C(12)	41(5)	96(8)	124(10)	32(8)	-38(6)	9(5)
C(13)	70(6)	93(8)	42(5)	29(5)	-27(4)	-47(6)
C(14)	58(6)	31(5)	120(9)	-36(5)	12(5)	-16(4)
C(15)	46(4)	53(5)	52(5)	28(4)	-15(4)	-23(4)

C(16)	49(4)	26(4)	62(5)	7(3)	-33(4)	-5(3)
C(17)	70(6)	33(5)	124(9)	46(5)	-70(6)	-27(4)
C(18)	39(5)	110(9)	90(7)	74(7)	-18(5)	-24(5)
C(19)	25(4)	124(8)	65(5)	69(6)	9(3)	13(4)
C(20)	22(3)	47(4)	43(4)	26(3)	-5(3)	-1(3)
C(21)	60(5)	47(5)	82(6)	12(5)	46(5)	10(4)
C(22)	38(4)	83(7)	72(6)	15(5)	10(4)	39(5)
C(23)	99(7)	39(5)	91(7)	24(5)	46(6)	45(5)
C(24)	42(5)	66(6)	69(6)	-36(5)	13(4)	3(4)
C(25)	78(6)	113(8)	38(4)	29(5)	24(4)	72(6)
C(26)	35(3)	26(3)	29(3)	4(3)	-3(3)	9(3)
C(27)	57(4)	35(4)	31(3)	10(3)	-3(3)	22(3)
C(28)	54(4)	33(4)	46(4)	20(3)	20(3)	18(3)
C(29)	23(3)	30(4)	44(4)	11(3)	13(3)	7(3)
C(30)	24(3)	22(3)	30(3)	5(3)	6(2)	13(2)
C(31)	370(30)	74(10)	250(20)	89(13)	290(30)	90(15)
C(32)	14(4)	203(19)	163(15)	-100(13)	25(6)	1(7)
C(33)	155(12)	113(10)	52(6)	20(6)	18(7)	113(10)
C(34)	83(7)	69(7)	63(7)	-37(6)	22(6)	3(6)
C(35)	217(17)	270(20)	33(5)	53(9)	39(8)	219(17)
C(36)	35(3)	25(3)	31(3)	9(3)	0(3)	3(3)
C(37)	48(4)	36(4)	35(4)	15(3)	-2(3)	7(3)
C(38)	50(4)	44(4)	46(4)	23(4)	14(3)	11(3)
C(39)	28(3)	28(4)	65(5)	11(3)	19(3)	3(3)
C(40)	26(3)	25(3)	35(3)	3(3)	3(2)	10(2)
P(1)	66(1)	35(1)	38(1)	16(1)	-3(1)	4(1)
P(2)	49(1)	30(1)	34(1)	9(1)	-3(1)	10(1)
F(1)	124(5)	83(4)	77(4)	34(3)	17(3)	-26(3)
F(2)	251(9)	67(4)	53(3)	29(3)	45(4)	-4(4)
F(3)	165(6)	119(6)	55(3)	-19(3)	26(4)	-79(5)
F(4)	114(5)	110(5)	61(4)	-5(3)	-14(3)	-6(4)
F(5)	114(6)	234(10)	270(11)	179(9)	64(6)	114(6)
F(6)	124(6)	162(7)	140(6)	30(5)	7(5)	83(5)
F(7)	54(3)	150(6)	145(6)	102(5)	9(3)	30(3)
F(8)	63(3)	101(4)	126(5)	49(4)	-14(3)	13(3)
F(9)	104(4)	60(3)	118(5)	57(3)	-24(3)	-2(3)
F(10)	144(6)	43(3)	159(6)	33(4)	-31(5)	13(3)
F(11)	161(6)	125(5)	56(3)	44(3)	33(3)	88(5)
F(12)	207(8)	194(8)	44(3)	18(4)	22(4)	129(7)
N(1)	89(6)	89(6)	113(7)	52(5)	50(5)	49(5)
C(41)	60(5)	45(5)	53(5)	21(4)	10(4)	21(4)
C(42)	59(5)	53(5)	58(5)	13(4)	11(4)	10(4)
N(2)	79(12)	95(14)	90(13)	24(11)	46(10)	1(10)
C(43)	52(11)	64(13)	36(9)	3(9)	20(7)	1(11)
C(44)	110(20)	110(30)	69(15)	24(16)	28(18)	60(20)

Table S17. Hydrogen coordinates (x 10^4) and isotropic displacement parameters ($Å^2$ x 10^3) for **4**.

	x	y	z	U(eq)
H(1)	6507	7455	8712	125
H(2)	6850	6610	9431	117
H(3)	5476	7236	10358	114
H(4)	4311	8502	10261	126
H(5)	4946	8699	9247	151
H(6)	2637	6003	8084	42
H(7)	2766	5140	8844	60
H(8)	1433	5940	9810	60
H(9)	373	7245	9633	47
H(11)	-4384	7072	5967	86
H(12)	-4388	7539	5056	108
H(13)	-3305	6429	4227	95
H(14)	-2460	5223	4664	107
H(15)	-3259	5701	5789	65
H(16)	-142	8164	6489	61
H(17)	-317	8811	5594	95
H(18)	698	7778	4657	93
H(19)	1658	6564	4963	76
H(21)	10138	4947	6925	78
H(22)	10611	3910	7501	75
H(23)	8138	2486	7152	84
H(24)	6092	2604	6326	90
H(25)	7345	4140	6168	81
H(26)	7846	5901	7991	37
H(27)	8073	4781	8564	47
H(28)	5440	3393	8145	49
H(29)	3586	3627	7307	39
H(31)	3376	9238	8183	253
H(32)	1653	9834	7552	203
H(33)	3378	11366	7621	114
H(34)	6132	11721	8282	106
H(35)	6159	10422	8638	173
H(36)	4263	8223	6771	38
H(37)	3782	9394	6272	47
H(38)	6521	10734	6594	54
H(39)	8692	10441	7321	50
H(42A)	1758	528	9460	88
H(42B)	901	912	8980	88
H(42C)	541	-177	8845	88
H(44A)	2954	-429	4820	140
H(44B)	4513	-362	5327	140
H(44C)	4848	-638	4612	140

Table S18. Torsion angles [°] for **4**.

C(5)-C(1)-C(2)-C(3)	1.3(12)
Co(1)-C(1)-C(2)-C(3)	-59.8(8)
C(5)-C(1)-C(2)-Co(1)	61.0(7)
C(1)-C(2)-C(3)-C(4)	-0.8(12)
Co(1)-C(2)-C(3)-C(4)	-59.7(7)
C(1)-C(2)-C(3)-Co(1)	58.9(7)
C(2)-C(3)-C(4)-C(5)	0.0(12)
Co(1)-C(3)-C(4)-C(5)	-60.8(7)
C(2)-C(3)-C(4)-Co(1)	60.8(8)
C(3)-C(4)-C(5)-C(1)	0.8(12)
Co(1)-C(4)-C(5)-C(1)	-60.8(7)
C(3)-C(4)-C(5)-Co(1)	61.6(8)
C(2)-C(1)-C(5)-C(4)	-1.2(11)
Co(1)-C(1)-C(5)-C(4)	60.7(7)
C(2)-C(1)-C(5)-Co(1)	-61.9(7)
C(10)-C(6)-C(7)-C(8)	-2.3(8)
Co(1)-C(6)-C(7)-C(8)	58.6(5)
C(10)-C(6)-C(7)-Co(1)	-60.9(4)
C(6)-C(7)-C(8)-C(9)	1.3(8)
Co(1)-C(7)-C(8)-C(9)	60.5(5)
C(6)-C(7)-C(8)-Co(1)	-59.2(5)
C(7)-C(8)-C(9)-C(10)	0.2(8)
Co(1)-C(8)-C(9)-C(10)	60.1(4)
C(7)-C(8)-C(9)-Co(1)	-59.9(5)
C(8)-C(9)-C(10)-C(6)	-1.5(7)
Co(1)-C(9)-C(10)-C(6)	57.1(4)
C(8)-C(9)-C(10)-Se(1)	177.9(5)
Co(1)-C(9)-C(10)-Se(1)	-123.4(4)
C(8)-C(9)-C(10)-Co(1)	-58.6(5)
C(7)-C(6)-C(10)-C(9)	2.3(7)
Co(1)-C(6)-C(10)-C(9)	-57.1(4)
C(7)-C(6)-C(10)-Se(1)	-177.1(5)
Co(1)-C(6)-C(10)-Se(1)	123.4(5)
C(7)-C(6)-C(10)-Co(1)	59.5(4)
Au(1)-Se(1)-C(10)-C(9)	-166.1(5)
Au(1)-Se(1)-C(10)-C(6)	13.2(6)
Au(1)-Se(1)-C(10)-Co(1)	105.4(3)
C(15)-C(11)-C(12)-C(13)	0.8(11)
Co(2)-C(11)-C(12)-C(13)	59.5(7)
C(15)-C(11)-C(12)-Co(2)	-58.7(6)
C(11)-C(12)-C(13)-C(14)	-1.2(10)
Co(2)-C(12)-C(13)-C(14)	58.4(6)
C(11)-C(12)-C(13)-Co(2)	-59.6(6)
C(12)-C(13)-C(14)-C(15)	1.2(9)
Co(2)-C(13)-C(14)-C(15)	61.7(5)
C(12)-C(13)-C(14)-Co(2)	-60.5(6)
C(12)-C(11)-C(15)-C(14)	0.0(10)
Co(2)-C(11)-C(15)-C(14)	-59.4(5)
C(12)-C(11)-C(15)-Co(2)	59.3(7)
C(13)-C(14)-C(15)-C(11)	-0.7(9)
Co(2)-C(14)-C(15)-C(11)	60.6(6)
C(13)-C(14)-C(15)-Co(2)	-61.3(5)
C(20)-C(16)-C(17)-C(18)	2.2(8)
Co(2)-C(16)-C(17)-C(18)	-59.3(5)
C(20)-C(16)-C(17)-Co(2)	61.5(5)
C(16)-C(17)-C(18)-C(19)	-1.8(9)
Co(2)-C(17)-C(18)-C(19)	-61.2(6)
C(16)-C(17)-C(18)-Co(2)	59.3(5)
C(17)-C(18)-C(19)-C(20)	0.8(9)
Co(2)-C(18)-C(19)-C(20)	-59.1(5)
C(17)-C(18)-C(19)-Co(2)	59.9(6)
C(17)-C(16)-C(20)-C(19)	-1.6(7)
Co(2)-C(16)-C(20)-C(19)	57.4(5)
C(17)-C(16)-C(20)-Se(2)	178.0(5)
Co(2)-C(16)-C(20)-Se(2)	-122.9(5)
C(17)-C(16)-C(20)-Co(2)	-59.0(4)
C(18)-C(19)-C(20)-C(16)	0.5(8)
Co(2)-C(19)-C(20)-C(16)	-57.5(5)
C(18)-C(19)-C(20)-Se(2)	-179.1(5)
Co(2)-C(19)-C(20)-Se(2)	122.8(5)
C(18)-C(19)-C(20)-Co(2)	58.0(5)
Au(1)-Se(2)-C(20)-C(16)	-5.5(6)
Au(1)-Se(2)-C(20)-C(19)	174.1(5)
Au(1)-Se(2)-C(20)-Co(2)	-98.3(4)
C(25)-C(21)-C(22)-C(23)	-1.0(9)
Co(3)-C(21)-C(22)-C(23)	59.4(6)
C(25)-C(21)-C(22)-Co(3)	-60.5(5)
C(21)-C(22)-C(23)-C(24)	0.7(9)
Co(3)-C(22)-C(23)-C(24)	59.5(6)
C(21)-C(22)-C(23)-Co(3)	-58.8(6)
C(22)-C(23)-C(24)-C(25)	-0.1(9)
Co(3)-C(23)-C(24)-C(25)	59.9(5)
C(22)-C(23)-C(24)-Co(3)	-59.9(6)
C(22)-C(21)-C(25)-C(24)	1.0(8)
Co(3)-C(21)-C(25)-C(24)	-59.9(5)
C(22)-C(21)-C(25)-Co(3)	60.8(5)
C(23)-C(24)-C(25)-C(21)	-0.5(9)
Co(3)-C(24)-C(25)-C(21)	59.8(5)
C(23)-C(24)-C(25)-Co(3)	-60.4(6)
C(30)-C(26)-C(27)-C(28)	1.0(7)
Co(3)-C(26)-C(27)-C(28)	-59.7(4)

C(30)-C(26)-C(27)-Co(3)	60.7(4)
C(26)-C(27)-C(28)-C(29)	0.3(7)
Co(3)-C(27)-C(28)-C(29)	-59.2(4)
C(26)-C(27)-C(28)-Co(3)	59.5(4)
C(27)-C(28)-C(29)-C(30)	-1.6(7)
Co(3)-C(28)-C(29)-C(30)	-60.6(4)
C(27)-C(28)-C(29)-Co(3)	59.0(5)
C(27)-C(26)-C(30)-C(29)	-2.0(6)
Co(3)-C(26)-C(30)-C(29)	57.4(4)
C(27)-C(26)-C(30)-Se(3)	178.1(4)
Co(3)-C(26)-C(30)-Se(3)	-122.5(5)
C(27)-C(26)-C(30)-Co(3)	-59.4(4)
C(28)-C(29)-C(30)-C(26)	2.2(6)
Co(3)-C(29)-C(30)-C(26)	-57.5(4)
C(28)-C(29)-C(30)-Se(3)	-177.9(4)
Co(3)-C(29)-C(30)-Se(3)	122.4(4)
C(28)-C(29)-C(30)-Co(3)	59.7(4)
Au(2)-Se(3)-C(30)-C(26)	-11.2(5)
Au(2)-Se(3)-C(30)-C(29)	168.9(4)
Au(2)-Se(3)-C(30)-Co(3)	-102.8(3)
C(35)-C(31)-C(32)-C(33)	0.0(16)
Co(4)-C(31)-C(32)-C(33)	-62.8(9)
C(35)-C(31)-C(32)-Co(4)	62.8(9)
C(31)-C(32)-C(33)-C(34)	0.1(14)
Co(4)-C(32)-C(33)-C(34)	-60.4(8)
C(31)-C(32)-C(33)-Co(4)	60.5(9)
C(32)-C(33)-C(34)-C(35)	-0.1(12)
Co(4)-C(33)-C(34)-C(35)	-59.3(7)
C(32)-C(33)-C(34)-Co(4)	59.1(8)
C(32)-C(31)-C(35)-C(34)	-0.1(16)
Co(4)-C(31)-C(35)-C(34)	62.3(8)
C(32)-C(31)-C(35)-Co(4)	-62.4(10)
C(33)-C(34)-C(35)-C(31)	0.2(13)
Co(4)-C(34)-C(35)-C(31)	-60.6(9)
C(33)-C(34)-C(35)-Co(4)	60.7(7)
C(40)-C(36)-C(37)-C(38)	-0.2(7)
Co(4)-C(36)-C(37)-C(38)	59.9(5)
C(40)-C(36)-C(37)-Co(4)	-60.2(4)
C(36)-C(37)-C(38)-C(39)	-1.0(8)
Co(4)-C(37)-C(38)-C(39)	58.8(5)
C(36)-C(37)-C(38)-Co(4)	-59.8(5)
C(37)-C(38)-C(39)-C(40)	1.8(8)
Co(4)-C(38)-C(39)-C(40)	60.4(4)
C(37)-C(38)-C(39)-Co(4)	-58.5(5)
C(37)-C(36)-C(40)-C(39)	1.3(7)
Co(4)-C(36)-C(40)-C(39)	-57.4(4)
C(37)-C(36)-C(40)-Se(4)	-179.5(5)
Co(4)-C(36)-C(40)-Se(4)	121.7(5)
C(37)-C(36)-C(40)-Co(4)	58.8(4)
C(38)-C(39)-C(40)-C(36)	-1.9(7)
Co(4)-C(39)-C(40)-C(36)	57.4(4)
C(38)-C(39)-C(40)-Se(4)	178.9(5)
Co(4)-C(39)-C(40)-Se(4)	-121.8(4)
C(38)-C(39)-C(40)-Co(4)	-59.4(4)
Au(2)-Se(4)-C(40)-C(36)	14.1(6)
Au(2)-Se(4)-C(40)-C(39)	-166.9(5)
Au(2)-Se(4)-C(40)-Co(4)	105.0(3)

Symmetry transformations used to generate equivalent atoms:
#1 -x + 1, -y + 1, -z + 2

5 Cell culture and Cytotoxicity

A-549, HT-29 and MDA-MB-231 cells were obtained from DSMZ (German Collection of Microorganisms and Cell Cultures GmbH, Braunschweig, Germany) and maintained by routine procedures. The cytotoxicity of the complexes was determined according to previously applied procedures.[3] In short: The test compounds were prepared as stock solutions in dimethylformamide and diluted with cell culture media to graded concentrations. The cells were exposed to the compound containing cell culture media for a period of 72 h (A-549, HT-29) or 96 h (MDA-MB-231), after which the cell biomass was determined by crystal violet staining. The IC_{50} values were calculated as the concentrations required to reduce cell proliferation by 50 % compared to the untreated control.

6 References

[1] S. Vanicek, H. Kopacka, K. Wurst, T. Müller, C. Hassenrück, R. F. Winter, B. Bildstein, *Organometallics* **2016**, *35*, *12*, 2101–2109

[2] S. Vanicek, M. Podewitz, C. Hassenrück, M. Pittracher, H. Kopacka, K. Wurst, T. Müller, K. R. Liedl, R. F. Winter, B. Bildstein, *Chem. Eur. J.* **2018**, *24*, 3165.

[3] C. Schmidt, L. Albrecht, S. Balasupramaniam, R. Misgeld, B. Karge, M. Brönstrup, A. Prokop, K. Baumann, S. Reichl, I. Ott, *Metallomics* **2019**, *11*, 533.

7 List of Figures

Figure S1: ^{1}H NMR (300 MHz, CD_3CN) **2a**. 3

Figure S2: ^{13}C NMR (75 MHz, CD_3OD) **2a**. 3

Figure S3: ^{77}Se NMR (57 MHz, CD_3OD) **2a**. 4

Figure S4: IR (ATR) **2a**. 4

Figure S5: UV-Vis (CH_3CN) **2a**. 5

Figure S6: MS (ESI+) **2a**. 5

Figure S7: ^{1}H NMR (300 MHz, CD_3CN) **2b**. 7

Figure S8: ^{1}H NMR (300 MHz, CD_3CN) **3**. 23

Figure S9: ^{13}C NMR (75 MHz, CD_3CN) **3**. 23

Figure S10: ^{77}Se NMR (57 MHz, CD_3CN) **3**. 24

Figure S11: ^{31}P NMR (121 MHz, CD_3CN) **3**. 24

Figure S12: IR (ATR) **3**. 25

Figure S13: UV-Vis (CH3CN) **3**. 25

Figure S14: MS (ESI+) **3**. 26

Figure S15: ^{1}H NMR (300 MHz, CD_3CN) **4**. 38

Figure S16: ^{13}C NMR (75 MHz, CD_3CN) **4**. 38

Figure S17: IR (ATR) **4**. 39

Figure S18: UV-Vis (CH_3CN) **4**. 39

Figure S19: MS (ESI+) **4**. 40

8 List of Tables

Table S1. Crystal data and structure refinement for **2b**. 8

Table S2. Atomic coordinates and equivalent isotropic displacement parameters for for **2b**. 9

Table S3. Bond lengths and angles for **2b**. 10

Table S4. Anisotropic displacement parameters for **2b**. 16

Table S5. Hydrogen coordinates and isotropic displacement parameters for **2b**. 18

Table S6. Torsion angles for **2b**. 19

Table S7. Crystal data and structure refinement for **3**. 27

Table S8. Atomic coordinates and equivalent isotropic displacement parameters for **3**. 28
Table S9. Bond lengths and angles for **3**. 29
Table S10. Anisotropic displacement parameters for **3**. 32
Table S11. Hydrogen coordinates and isotropic displacement parameters for **3**. 34
Table S12. Torsion angles for **3**. 34
Table S13. Crystal data and structure refinement for **4**. 41
Table S14. Atomic coordinates and equivalent isotropic displacement parameters for **4**. 42
Table S15. Bond lengths and angles for **4**. 43
Table S16. Anisotropic displacement parameters for **4**. 50
Table S17. Hydrogen coordinates and isotropic displacement parameters for **4**. 52
Table S18. Torsion angles for **4**. 53
